# Unraveling the Potential of Vitamin B_3_-Derived Salts with a Salicylate Anion as Dermal Active Agents for Acne Treatment

**DOI:** 10.1021/acs.molpharmaceut.4c00543

**Published:** 2024-08-14

**Authors:** Adriana Olejniczak, Witold Stachowiak, Daniel Ziental, Jolanta Długaszewska, Tomasz Rzemieniecki, Marcin Wysokowski, Teofil Jesionowski, Michał Niemczak

**Affiliations:** † Faculty of Chemical Technology, Poznan University of Technology, Berdychowo 4, Poznan 60-965, Poland; ‡ Chair and Department of Inorganic and Analytical Chemistry, Poznan University of Medical Sciences, Rokietnicka 3, Poznan 60-806, Poland; § Chair and Department of Genetics and Pharmaceutical Microbiology, Poznan University of Medical Sciences, Rokietnicka 3, Poznan 60-806, Poland

**Keywords:** nicotinamide, salicylic acid, acne vulgaris, active pharmaceutical ingredients, API, antimicrobial activity

## Abstract

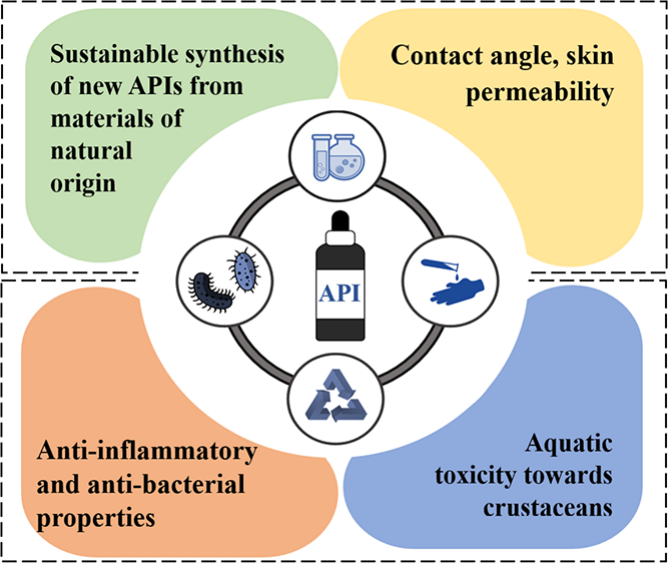

This study is focused on the utilization of naturally occurring salicylic acid and nicotinamide (vitamin B_3_) in the development of novel sustainable Active Pharmaceutical Ingredients (APIs) with significant potential for treating acne vulgaris. The study highlights how the chemical structure of the cation significantly influences surface activity, lipophilicity, and solubility in aqueous media. Furthermore, the new ionic forms of APIs, the synthesis of which was assessed with *Green Chemistry* metrics, exhibited very good antibacterial properties against common pathogens that contribute to the development of acne, resulting in remarkable enhancement of biological activity ranging from 200 to as much as 2000 times when compared to salicylic acid alone. The molecular docking studies also revealed the excellent anti-inflammatory activity of *N*-alkylnicotinamide salicylates comparable to commonly used drugs (indomethacin, ibuprofen, and acetylsalicylic acid) and were even characterized by better IC_50_ values than common anti-inflammatory drugs in some cases. The derivative, featuring a decyl substituent in the pyridinium ring of nicotinamide, exhibited efficacy against *Cutibacterium acnes* while displaying favorable water solubility and improved wettability on hydrophobic surfaces, marking it as particularly promising. To investigate the impact of the APIs on the biosphere, the EC_50_ parameter was determined against a model representative of crustaceans—*Artemia franciscana*. The majority of compounds (with the exception of the salt containing the dodecyl substituent) could be classified as “Relatively Harmless” or “Practically Nontoxic”, indicating their potential low environmental impact, which is essential in the context of modern drug development.

## 1 Introduction

In the dynamic landscape of pharmaceutical industry, the search for effective and versatile active ingredients remains of the greatest importance.^1−3^ Among the countless compounds being investigated for their therapeutic potential, nicotinamide (**NA**) and salicylic acid (**SAL**) stand out as remarkable entities, offering numerous benefits and applications in the healthcare field, including well-known antiacne properties.

Acne vulgaris is a chronic inflammatory disease that mainly appears in the teenage years and persists into adulthood, affecting about 85–90% of the world’s population, at some stage in life. Its clinical characteristics include seborrhea (excess grease), various forms of inflammatory lesions, and scarring. In practice, treating acne involves two key aspects: removing the root cause by combating pathogenic microorganisms to prevent future skin lesions and managing symptoms by reducing inflammation, redness, and swelling to improve skin comfort and appearance. Therefore, the great importance of treating acne vulgaris is emphasized not only by its impact on physical appearance but also on the mood and self-confidence of those affected. Given its common nature, there is still a significant need for new innovative therapies.^4−8^

**NA**, also known as niacinamide or vitamin B_3_, is a multifaceted compound with a diverse array of biological activities, beneficial effects on skin health, and neuroprotection. Its ability to strengthen the skin’s barrier function and reduce inflammatory reactions has made it an important active ingredient in the treatment of dermatological conditions such as acne vulgaris and rosacea.^9−11^**SAL** has been recognized for its strong anti-inflammatory and keratolytic properties. Well-known advantages of both **NA** and **SAL** underline the importance of implementing them in therapeutic formulations to support overall systemic health.^12−15^

The individual potency of **NA** and **SAL** in the treatment of various dermatological conditions has long been known, but it is their synergistic potential that really captures the imagination both of researchers and clinicians recently.^16,17^ Preliminary studies suggest that **NA** augments the anti-inflammatory and barrier-stabilizing effects of **SAL**, thus enhancing its therapeutic effectiveness under dermatological conditions characterized by inflammation and impaired barrier function. Fascinatingly, their combination may offer a multifaceted approach to addressing complex dermatological conditions, targeting multiple pathogenic pathways simultaneously.^18,19^

Currently, there are numerous reports that focus on the derivatization of **NA** that is widely known as an attractive resource for chemical syntheses because of not only its rich range of properties but also economic viability. In the past, various attempts were made to modify its structure by attaching alkyl substituents to the nitrogen in its pyridine ring.^20−24^ The most important findings in this field are summarized in the Figure S1 (in the Supporting Information). Ionic derivatives of **NA** formed by exchange of a halide anion for an ion showing biological activity are also known.^25^ The salt with a quaternary nicotinamide cation and salicylate anion has been described previously, however only for one specific length of the alkyl chain—hexyl.^18^ It should also be noted that its synthesis method and spectral data have not been provided. Therefore, it justifies the need for development of the knowledge in this area and fills the scientific gap regarding a homologous series of new Active Pharmaceutical Ingredients (APIs) obtained from **NA** and **SAL**.

In this study, we focused on exploring multiple aspects associated with the use of **NA** and **SAL** as different forms of APIs. We aimed to elucidate their individual contributions to dermatological health and unravel their potential depending on an applied form (cocrystal vs. organic salt). Furthermore, modification of the nicotinamide structure by quaternization of the nitrogen atom in the aromatic ring offers the potential to adjust the physicochemical properties of the APIs, such as water solubility or hydrophobicity, and also their biological activity. By delving into the interaction between both APIs, we aim to pave the way for the development of new therapeutic approaches to meet current medical needs and improve the treatment of common diseases.

## 2 Materials and Methods

### 2.1 Materials

Bromoethane (98%), 1-bromobutane (99%), 1-bromohexane, (99%), 1-bromooctane (99%), 1-bromodecane (98%), 1-bromododecane (97%), 1-bromotetradecane (97%), octan-1-ol (99%), nicotinamide (98%), and salicylic acid (99%) were purchased from Sigma-Aldrich (Saint Louis, MO, USA). All solvents (methanol (99.8%), ethanol (96%), acetonitrile (99%), acetone (99%)), *n*-propanol (99.5%), and potassium hydroxide (99%)) were obtained from Avantor (Gliwice, Poland) and used without further purification. Phosphate suffer saline (PBS) solution was prepared by dissolving 8.0 g of NaCl, 0.2 g of KCl, 1.42 g of Na_2_HPO_4_, and 0.24 g of KH_2_PO_4_ in 1 L of deionized water. Its pH was adjusted to 7.4 using HCl solution. Artificial seawater was prepared by dissolving 26.4 g of NaCl, 0.84 g of KCl, 1.67 g of CaCl_2_·2H_2_O, 4.60 g of MgCl_2_·6H_2_O, 5.58 g of MgSO_4_·7H_2_O, 0.17 g of NaHCO_3_, and 0.03 g of H_3_BO_3_ in 1 L of deionized water. Deionized water with a conductivity of <0.1 μS cm^–1^, from a Hydrolab HLP Smart 1000 demineralizer (Straszyn, Poland), was used.

### 2.2 General

^1^H NMR spectra were recorded on a Varian VNMR-S 400 MHz spectrometer (Palo Alto, USA) with TMS as the internal standard. ^13^C NMR spectra were obtained with the same instrument at 100 MHz. The FTIR spectra were collected by using an EasyMax 102 semiautomated system (METTLER TOLEDO, Switzerland) connected to a ReactIR iC15 (METTLER TOLEDO, Switzerland) probe equipped with an MCT detector and 9.5 mm AgX probe with a diamond tip. The data were sampled from 3000 to 650 cm^–1^ with 8 cm^–1^ resolution and processed by iCIR 4.3 software. The water content in all obtained products was measured with a TitroLine 7500 KF trace apparatus (SI Analytics, Germany) using the Karl Fischer titration method. First, each compound was dissolved in dehydrated methanol. The water content was determined in pure methanol as well as in the obtained methanolic solutions. On the basis of the collected results, the water content in pure products was calculated. The residual concentration of bromide ions was performed based on the method described in the literature.^25^ First, 1 ± 0.0001 g of synthesized products was introduced into a 100 mL measuring flask, which was then filled with deionized water. Subsequently, the obtained solution was mixed in a beaker with 1 mL of 5% potassium chromate (K_2_CrO_4_) solution. Next, the titration process was carried out using a 0.1 mol·L^–1^ silver nitrate (AgNO_3_) solution, accompanied by vigorous stirring. The titration continued until a consistent brown-red suspension was achieved.

### 2.3 Synthesis

#### 2.3.1 Preparation of *N*-Alkylnicotinamide Salicylates (**1**–**7**)

Initially, salicylic acid (**SAL**) was neutralized with stoichiometric amounts of potassium hydroxide in methanol using an EasyMax reactor (METTLER TOLEDO, Switzerland) equipped with a pH-meter. Then, the solvent was evaporated using a rotary vacuum evaporator. The obtained potassium salicylate (**[K][SAL]**) was dried in a vacuum oven at 40 °C for 48 h. Next, *N*-alkylnicotinamide bromides (**B1**–**B7**) were obtained according to the methodology described by Stachowiak et al.^26^

**Figure 1:**
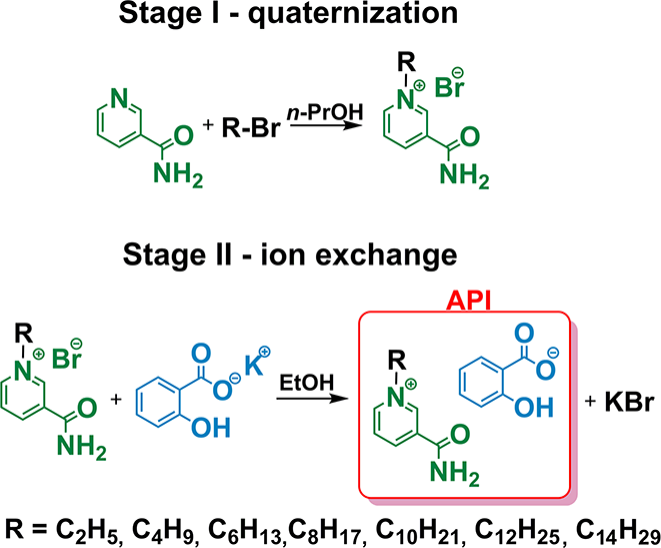
Synthesis of *N*-alkylnicotinamide salicylates (**1**–**7**).

All metathesis reactions were conducted using an EasyMax reactor (METTLER TOLEDO, Switzerland) equipped with a SevenMulti pH-meter connected to an InLab 1022 pH electrode, due to the fact that, in a basic environment, the nicotinamide moiety undergoes decomposition toward red-brown impurities. Consequently, the 0.01 mol of appropriate *N*-alkylnicotinamide bromide (**B1**–**B7**) was dissolved in 10 mL of ethanol in a round-bottomed flask equipped with a mechanical stirrer and pH electrode. Next, **[K][SAL]** dissolved in 10 mL of ethanol was added with a 2% of molar excess (0.0102 mol) to perform the ion exchange reaction (Figure 1). The reaction mixture was stirred at 40 °C for 15 min and then cooled to 0 °C. As a result of anion exchange, a sediment of potassium bromide precipitated from the postreaction mixture.

Subsequently, the inorganic salt was filtered off, and the solvent was evaporated from the filtrate. The obtained products were additionally purified by the addition of a small portion (10–15 mL) of acetone, which allowed us to isolate the residues of inorganic impurities and the excess reactant through vacuum filtration. Following the evaporation of solvents, the obtained products were dried at 50 °C for 24 h under reduced pressure (1–2 mbar). All synthesized salts were stored in a vacuum desiccator with a drying agent (P_4_O_10_).

#### 2.3.2 Preparation of Nicotinamide and Salicylic Acid Cocrystal ([NA][SAL])

**Figure 2:**
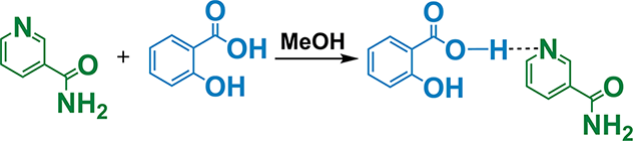
Synthesis of nicotinamide-salicylic acid cocrystal **[NA][SAL]**.

The preparation of nicotinamide and salicylic acid cocrystals was accomplished by mixing the appropriate amount of nicotinamide (**NA**) with the equimolar amount of salicylic acid (**SAL**), dissolved in methanol, and stirred for 2 h at room temperature (Figure 2). The remaining solvent was removed using a rotary evaporator.

### 2.4 Melting Point

The melting points of the compounds obtained were analyzed via an MP90 Melting Point System (Mettler Toledo, Switzerland). The precision of the measurements was ensured by the calibration of the apparatus using certified reference substances.

### 2.5 Octanol–Water Partition Coefficient

The octanol–water partition coefficients (*K*_OW_) of the synthesized products were estimated by the shake-flask method according to the OECD Test No. 107 guidelines (Partition Coefficient *n*-Octanol/Water: Shake-Flask Method). Measurements were performed using mutually saturated distilled water and 1-octanol in a glass vial containing a magnetic stir bar. First, the synthesized products were dissolved in distilled water (or 1-octanol) at a chosen concentration (0.01 mol per 1 L of octanol or water), and then proper amounts of the second solvent (octanol or water) were added. Subsequently, two duplicate runs were carried out with different solvent ratios: 4 mL of 1-octanol and 2 mL of water (2:1), 4 mL of 1-octanol and 4 mL of water (1:1), and 4 mL of 1-octanol and 8 mL of water (2:1). All vials were shaken at a constant temperature of 25 °C. After 24 h, all samples were centrifuged and the aqueous and octanolic phases were collected with a syringe. The concentrations of compounds in water were determined spectrophotometrically using a Rayleigh UV-1601 spectrophotometer (Beijing Beifen-Ruili Analytical, China) based on calibration curves made previously (at λ_max_ = 262 nm) vs concentration for each substance. Two repetitions of each measurement were performed in a specific solvent ratio (1:1, 1:2, and 2:1). Finally, the log *K*_OW_ was calculated as the average of six results collected for each compound.

### 2.6 Skin Permeability Coefficient

The skin permeation coefficient (Kp) was determined using a Skin Permeation Calculator provided by the National Institute for Occupational Safety & Health (NIOSH).^27^ This calculator estimates the Kp value from a water vehicle using three different models: Frasch, Potts and Guy, and Modified Robinson.

### 2.7 Solubility in PBS

The solubility in PBS solution was determined by placing 1 g (±0.0001) of tested compound in a round-bottom flask and adding specific parts of the PBS solution in portions until full dissolution. The samples were vigorously stirred for at least 5 min before the addition of the next portion of PBS, and the systems were thermostated at 25 °C. Three repetitions of the measurement were performed for each of the compounds.

### 2.8 Contact Angle Measurements

The contact angle (CA) measurements were carried out using a DSA100S analyzer (Krüss, Germany, accuracy ±0.1°), at 25 °C. The determination of the contact angle was based on the sessile drop method, i.e., drops of the analyzed solution were deposited on a solid surface (paraffin). The images of the drops were taken with a CCD camera, digitized, and evaluated using Young–Laplace fitting. The CA was determined as the slope of the tangent line at the contact point between the 3 phases (solution, paraffin surface, and air).

### 2.9 Microorganisms and Culture Media

The antimicrobial activities of the compounds were tested against clinical strains of an aerobic Gram-negative bacterium—*Pseudomonas aeruginosa* (*P. aeruginosa*), facultative anaerobic Gram-positive bacteria—*Staphylococcus aureus* (*S. aureus*) and *Staphylococcus epidermidis* (*S. epidermidis*), anaerobic Gram-positive bacterium—*Cutibacterium acnes* (*C. acnes*), and yeast-like fungus—*Candida albicans* (*C. albicans*).

Aerobic and facultative anaerobic bacteria cultures were grown in Brain Heart Infusion broth (BHI, bioMerieux, France) at 35 °C ± 1 °C for 18 h, *C. acnes* cultures were grown in thioglycolate broth with resazurin (TGB, bioMerieux, France) at 35 ± 1 °C for 48 h, and yeast cultures were grown in Sabouraud dextrose broth (SDB, Merck, Germany) at 35 ± 1 °C for 24 h. After incubation, each culture was diluted in a suitable liquid medium: *P. aeruginosa*, *S. aureus*, and *S. epidermidis*—Mueller–Hinton broth (MHB; Oxoid, UK); *C. acnes*—TGB; and *C. albicans*—SDB, to obtain a final suspension containing about 10^6^ CFU/mL.

### 2.10 MIC and MBC Determination

The microdilution method was employed to determine the compounds’ MIC (Minimal Inhibitory Concentration) and MBC/MFC (Minimal Bactericidal Concentration/Minimal Fungicidal Concentration) against the tested bacterial and fungal strains.

The compounds were serially diluted (2-fold) in the respective media (*P. aeruginosa*, *S. aureus*, and *S. epidermidis*—MHB, *C. acnes*—TGB, and *C. albicans*—SDB in 96-well plates), and the microbial suspensions were added. The compounds were tested in the final concentration range of 4 096 to 0.125 μg/mL, and the final microbial inoculum was approximately 5 ×10^5^ CFU/mL. The tests were incubated at 35 °C ± 1 °C for 18 h (*P. aeruginosa*, *S. aureus*, and *S. epidermidis*) or 24 h (*C. albicans*) or 48 h (*C. acnes*). *C. acnes* was incubated in an anaerobic atmosphere using a GENbag anaer (bioMerieux, France). Media (without the strains) added to the different concentrations of tested compounds and media inoculated with microbial suspension were used as a negative control and growth control, respectively.

The MIC was defined as the lowest concentration at which visible growth was inhibited. MBC and MFC concentrations were determined as an extension of the MIC test. After performing the MIC test and recording the MIC end point, every well that demonstrated no growth (concentration equal to and greater than MIC) was subcultured onto an agar medium: Typcase Soy Agar (TSA; bioMerieux)—*P. aeruginosa*, *S. aureus*, and *S. epidermidis*, Columbia Agar with sheep blood (CA; Thermo Scientific, UK)—*C. acnes*, and Sabouraud dextrose agar (SDA; Merck, Germany)—yeast. The plates were incubated at 35 ± 1 °C for 18–72 h. *C. acnes* was incubated in an anaerobic atmosphere. The MBC/MFC was defined as the lowest concentration at which no growth was observed. All tests were performed in duplicate.

### 2.11 Molecular Docking

Docking studies were performed using the AutoDock4 program in AutoDockTools 1.5.7.^28^ The structures for ligands (reference substances and *N*-alkylnicotinamide cations) were optimized for energy minimization using the MMFF94 force field in Avogadro 1.2.0 with the steepest descent algorithm and subsequently converted to a PDBQT format using OpenBabelGUI. The three-dimensional structure of target cyclooxygenase-2 (Protein Data Bank ID: 1CX2) was retrieved from Protein Data Bank (http://www.pdb.org) with a resolution of 3.00 Å. The protein molecule 1CX2 was prepared using AutoDockTools version 1.5.7. The preparation process involved checking for missing atoms, repairing any missing atoms, adding polar hydrogens and Kollman charges, and distributing charges over all of the atoms on residues. All cocrystallized compounds present in the 1CX2 structure (including water molecules and the ligand) were removed prior to the calculations. After the preparation of the receptor, the Lamarckian Genetic Algorithm (LGA) was utilized to search for conformations using the following docking parameters: a population size of 300 dockings, a maximum number of generations of 27,000, a maximum energy evaluation of 2.5 million, and 50 docking runs.^29^ Other parameters were used in default mode. The grid box was set with center coordinates *x* = 20.99; *y* = 23.20; and *z* = 17.48 and size *x* = 40; *y* = 40; and *z* = 40 centered on the predicted cavities with a spacing of 0.500 Å. The Autogrid4 program was used to generate grid maps. The conformation with the least binding energy was considered to be the most favorable docking pose. The interaction between the ligand and receptor and hydrogen bond lengths were analyzed using AutoDockTools and PLIP (Protein Ligand Interaction Profiler) developed by Biotechnology Center TU Dresden (BIOTEC).

### 2.12 Ecotoxicity toward Aquatic Life

To determine the EC_50_ parameter for compounds, tests were carried out on marine crustaceans—*Artemia franciscana* (*A. franciscana*). The methodology proposed in the Artoxkit M test (MicroBioTests Inc., Gent, Belgium) was developed according to the ASTM E1440-91 standard.

The hatching process was started first. For this purpose, 50 mg of cysts was transferred to the Petri dishes attached to the Artoxkit M set, which were then immersed in 10 mL of artificial seawater with a salinity of 35‰ (a medium for the development of the tested organisms, as well as a solvent for preparing solutions of the tested compounds) and placed at a temperature of 25 °C, with access to light (6000–10,000 lux) for 30 h. Then, 2 h before placing them on the Artoxkit M plates, 20 mg of spirulina was added to cultured artemias. In addition, 1 h before the analysis, the used seawater was oxygenated by passing a stream of air through the solution. To each of the four cells in a given row of a plate from the Artoxkit M kit, 1 mL of solution at the specified concentrations (or the appropriate medium in the case of a control) were introduced. In the first column of cells (a control sample and five selected concentrations), no less than 30 artemias were placed from previously prepared Petri dishes. Then, from the first cell in the first column, artemias were collected at the selected concentration and added to the next three cells in the selected row, 10 organisms per cell. After the organisms were introduced into the appropriate cells, the plates were covered with Parafilm and then closed with a plastic cap. The prepared kit was incubated at 25 °C and protected from light. Number of motionless organisms were counted after 24 and 48 h. Immobilization was then calculated (eq 1) in relation to the number of organisms at the start of the test:
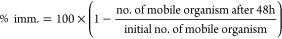
1

Next, the dependence between the effect and concentration of the tested compounds was plotted, and on this basis, an EC_50_ value was determined.

## 3 Results and Discussion

### 3.1 Synthesis of New APIs

The simple mixing of nicotinamide and salicylic acid results in a formation of an API that is already known in the literature and has been recognized as cocrystal—a solid substance made up of neutral molecules,^30^ in which hydrogen bonds form between the nitrogen atom in the pyridine ring and the hydrogen atom of the carboxyl group of salicylic acid. Thus, there is no characteristic proton transfer leading to formation of an ionic bond and allowing classification of the obtained cocrystal (**[NA][SAL]**) as an organic salt. It is worth mentioning that nicotinamide (**NA**) has been used to form other cocrystals previously, e.g., a cocrystal of **NA** with celecoxibe, a commercially available drug that exhibits excellent bioavailability when administered in a suspension form,^31^ or a cocrystal with ferulic acid, where the presence of **NA** facilitates the solubility of the acid and allows it to be implemented in topical formulations.^32^

Nowadays, the development of new pharmaceuticals requires not only coping with problems such as polymorphism or low stability but also consciously designing them from raw materials that are safe and readily accessible while keeping in mind the economic viability for their potential mass production.^33,34^ In this context, the strategy proposed by Rogers et al. that is based on transformation of API into organic salt containing ionic bond, preferably ionic liquids (ILs), can become an even more beneficial approach compared to the formation of API-based cocrystals.^3^ Following this strategy, properly designed organic salts originating from **NA** and **SAL** both of which are APIs used individually for therapeutic purposes can be successfully synthesized. Although this approach requires insertion of an alkyl chain into the nicotinamide moiety, it should be stressed that this action should contribute to enhancing biological (e.g., bactericidal) activity as confirmed for many quaternary ammonium salts of the amphiphilic structure, which encourages us to seek an optimal alkyl chain length in the homologous series of such APIs. Consequently, the successful combination of the *N*-alkylnicotinamide cation with the salicylate anion may lead to new multifunctional APIs, representing quaternary ammonium salts (QASs), with increased efficacy or a substantially broader spectrum of activity than starting constituents.

**Figure 3:**
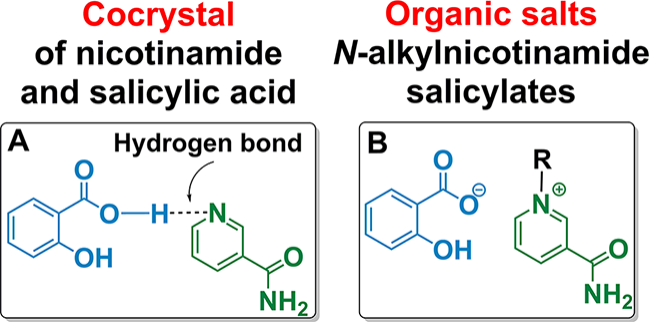
Tested forms of APIs originate from nicotinamide and salicylic acid.

To investigate the above-mentioned hypothesis and seek for potential benefits from utilization of the selected APIs (Figure 3), a homologous series of new compounds, *N*-alkylnicotinamide salicylates wherein alkyl varies from ethyl to tetradecyl (**1**–**7**), was obtained in a sustainable two-step synthesis that comply with *Green Chemistry* principles. Subsequently, their potential as effective novel APIs was assessed in comparison to parent compounds (**NA** and **SAL**) as well as the obtained cocrystal ([**NA**][**SAL**]).

A salt with six carbon atoms in the alkyl chain, designated number **3** among the compounds tested, has already been described by Dobler et al.,^18^ although the method of obtaining it has not been elucidated. Therefore, it will be important to describe it, adding to the state of knowledge on the subject. The synthesis of **1**–**7** involved a quaternization with appropriate alkyl bromide, followed by an ion exchange reaction carried out in ethanol, where the bromide anion was substituted with the salicylate ion. Total yields of both stages were high, reaching 90% (Table 1). The content of bromide ions in the obtained salicylates, determined using Mohr titration, varied from approximately 3000 to 8500 ppm (see Table S1 in the Supporting Information). Collected results show that ion exchange leads to a substantial decrease salicylates’ (**1**–**7**) melting points compared to the respective bromides (**B1**–**B7**). In effect, four salicylates (**2**–**4** and **6**) exhibit melting below 100 °C and can be classified as ionic liquids.^35,36^ The most significant melting point depression (by as much as 124 °C) was observed for **6**, while the least notable reduction amounting to 36 °C was observed for **1**.

**Table 1:** Melting Point of *N*-Alkylnicotinamide Salicylates (**1**–**7**) and *N*-Alkylnicotinamide Bromides (**B1**–**B7**)

	bromides			salicylates			
no.	**R**	**melting point [°C]**	**no.**	**R**	**melting point [°C]**	yield [%]	melting point depression^a^ [°C]
B1	**C**_**2**_**H**_**5**_	decay at 146	**1**	**C**_**2**_**H**_**5**_	111	90	35^b^
B2	**C**_**4**_**H**_**9**_	153	**2**	**C**_**4**_**H**_**9**_	78	85	75
B3	**C**_**6**_**H**_**13**_	175	**3**	**C**_**6**_**H**_**13**_	98	86	77
B4	**C**_**8**_**H**_**17**_	185	**4**	**C**_**8**_**H**_**17**_	99	88	86
B5	**C**_**10**_**H**_**21**_	decay at 205	**5**	**C**_**10**_**H**_**21**_	117	78	88^b^
B6	**C**_**12**_**H**_**25**_	decay at 207	**6**	**C**_**12**_**H**_**25**_	84	82	123^b^
B7	**C**_**14**_**H**_**29**_	decay at 206	**7**	**C**_**14**_**H**_**29**_	108	87	98^b^

a Between bromides and salicylates.

b Due to the decomposition of the compound before melting, the value of the beginning of the decomposition was taken for the calculations.

Structures of the synthesized salts **1**–**7** were confirmed with the use of various spectroscopic methods, such as UV, FTIR, and ^1^H and ^13^C NMR spectroscopies. All spectra are provided in the Supporting Information (Figures S2–S41). The characteristic differences in the location of peaks at the ^1^H NMR spectra between substrates (**[NA]** and **[SAL]**), cocrystal **[NA][SAL]**, and one representative product with decyl chain (**5**) are demonstrated in Figure S42 in the Supporting Information. It clearly illustrates the structural disparity between the ionic substance and cocrystal, in which its components interact only through hydrogen bonding and therefore its peaks are not shifted and remain in the region corresponding to the starting substrates.

Examination of the water content in the obtained salts **1**–**7** showed that they contain approximately 0.5–2.6% water after drying (see Table S1 in the Supporting Information). The water content determined for [**NA**][**SAL**] (approximately 1.1%) exceeded the values recorded for the individual substrates (0.78% for **NA**, 0.26% for **SAL**), which may indicate the presence of specific interactions in it, affecting its hydrophilic properties. However, compounds **1**–**7** synthesized in the framework of this study exhibited minimal hygroscopicity when exposed to air, leading to the conclusion that the moisture content is not a result of water molecules binding to the crystal lattice of the obtained compounds.

**Figure 4:**
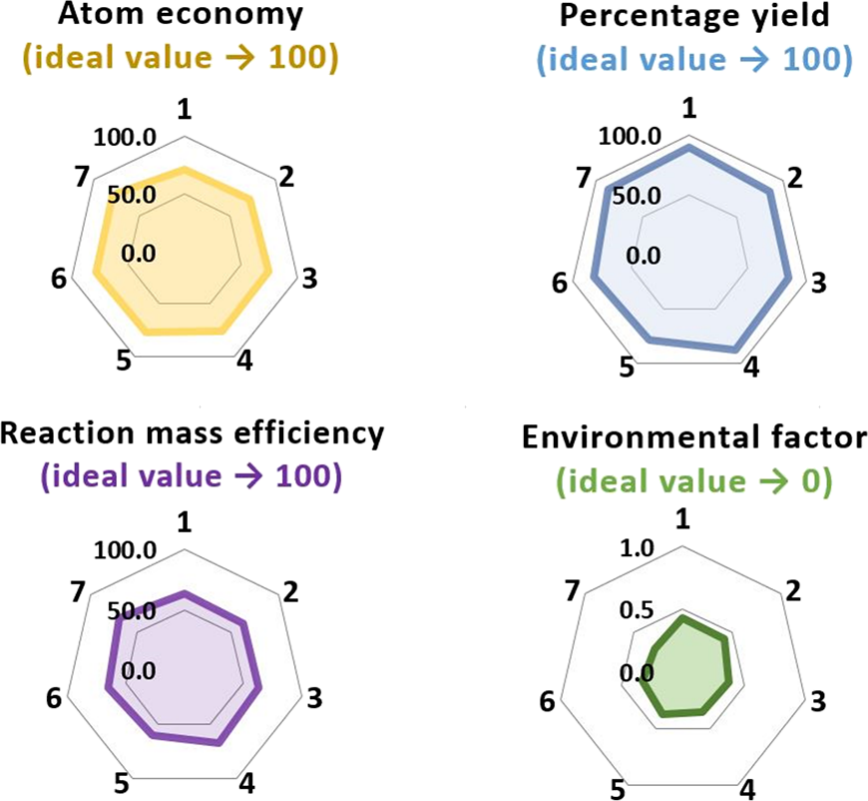
Radar plot of *Green Chemistry* metrics determined for *N*-alkylnicotinamide salicylates (**1**–**7**).

It should be noted that maintaining the pH of all reaction mixtures slightly below neutral value was crucial, given that the nicotinamide moiety is susceptible to rapid decomposition in a basic environment.^37^ It is important to emphasize the implementation of ethanol as an anion exchange environment. The introduction of this solvent instead of the commonly used methanol, even a small amount of which could prove toxic to the human body, offsets the need for prolonged drying to remove it from the final product. Moreover, the presence of ethanol residues in the synthesized product will not pose any danger (as in the case of methanol) since ethanol is often applied as a permeation enhancer in commercially available topical formulations.^38^ In addition, potassium bromide, a byproduct in the proposed methodology that may be considered a waste, may easily be purified and utilized, e.g., as a medication to assist in the treatment of epilepsy in dogs.^39^ This makes the presented synthetic route promising not only due to its simplicity, safety, or cost-effectiveness but also because it does not generate waste that would later have to be properly managed, generating unnecessary production streams. Moreover, increasing environmental concerns are forcing researchers to carefully analyze the syntheses they perform with the key parameters in mind. Therefore, to assess the efficiency and overall environmental safety of the *N*-alkylnicotinamide salicylates, *Green Chemistry* metrics were determined and their values are provided in Figure 4 (specific data are shown in Table S2 in the Supporting Information).^40^

The environmental factor (E-factor, ideal value = 0) for compounds **1**–**7** takes values from 0.29 to 0.43, while values typical for the pharmaceutical industry are in the range of 25–100. Thus, we can conclude that the methodology developed for the *N*-alkylnicotinamide salicylates synthesis allows for up to a 300-fold reduction in a negative environmental impact compared to average APIs synthesis processes.^41^ No such advantages were noted when comparing the calculated values of the other parameters with those typically achieved in the pharmaceutical industry. The least favorable values can be noted for reaction mass efficiency (from 60.44 for **5** to 69.19% for **7**), and percentage yield (<90.00%) in the case of all compounds except **1**.

### 3.2 Physicochemical Parameters of APIs Determining Their Susceptibility for Dermal Application

#### 3.2.1 Molecular Mass and Melting Point

The unique attributes of the human skin include its role as a physicochemical barrier with the ability to effectively impede the penetration of many molecules. *The 500 Da Rule* states that for a molecule to effectively penetrate into the skin layers and therefore be successfully applied dermally, its molecular weight (MW) must be below the 500 Da (Da) threshold. This thesis is supported by the fact that most of the commonly used pharmacological agents in topical dermatotherapy meet the above criterion.^42^ Moreover, the relevance of the described idea is supplemented by the fact that compounds such as cyclosporine (1202 Da), a promising drug used to treat psoriasis,^43^ have demonstrated no efficacy when employed in topical dermatological treatments. Interestingly, when injected intramuscularly, it manifests as a proficient therapeutic agent, highlighting the influence of molecular mass of the active ingredient on its ability to permeate through the skin.^44,45^ It should be emphasized that **NA** and **SAL** had the lowest molecular mass values (122 and 138 Da, respectively), prompting consideration of their widespread in commercial cosmetic products. Given that relatively small molecules can penetrate deeply into the skin and readily enter the bloodstream, it highlights how crucial it is to develop appropriate formulations to ensure retention of the APIs on the outer layers of the skin, where the therapeutic effect should occur. Intriguingly, all of the *N*-alkylnicotinamide salicylates (**1**–**7**) meet the requirement of the *The 500 Da Rule*, ranging from 288 Da (**1**) to 457 Da (**7**, see Table S3 in the Supporting Information), thus presenting auspicious prospects for dermal application. Cocrystal ([**NA**][**SAL**]) also can be considered a potential topical agent, as its mass (260 Da) is only slightly higher compared to that of, e.g., acyclovir (225 Da), whose commercial forms are often intended for topical application.

The relationship between melting point and skin permeability is inversely correlated.^46^ Compounds with high melting points, characterized by limited solubility in both water and fat, typically pose challenges in dermal drug delivery,^47^ while low-melting substances are more often readily soluble within the stratum corneum.

In light of recent reports that suggest the use of numerous ionic liquids as topical and transdermal drug delivery systems due to their enhanced skin permeability, it is worth noting that four of the synthesized salts (**2**–**4** and **6**) melt at temperatures below 100 °C and can therefore be classified as ionic liquids.^3,35,48^

**Figure 5:**
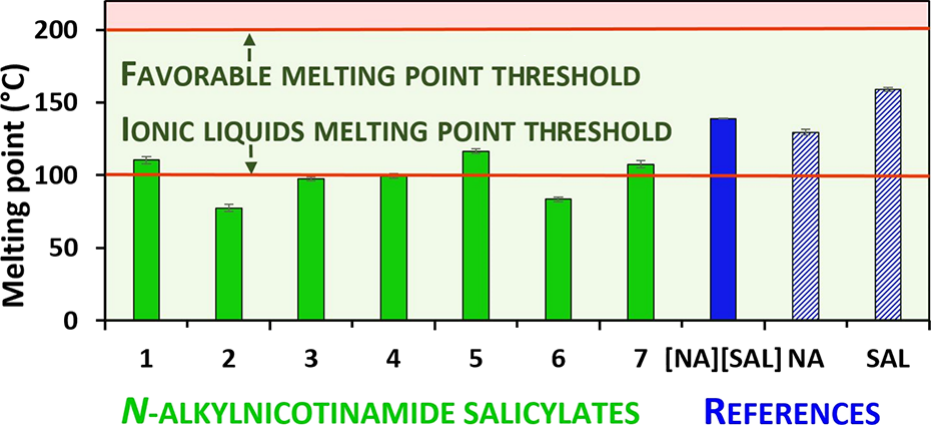
Melting point of *N*-alkylnicotinamide salicylates (**1**–**7**) and reference substances: cocrystal of nicotinamide and salicylic acid ([**NA**][**SAL**]), nicotinamide (**NA**), and salicylic acid (**SAL**).

As shown in Figure 5, all of the synthesized products (**1**–**7**) exhibit melting point values required for dermal drug delivery (below 200 °C),^49^ which enables further consideration of their application as topical APIs.

### 3.3 Octanol–Water Partition Coefficient

**Figure 6:**
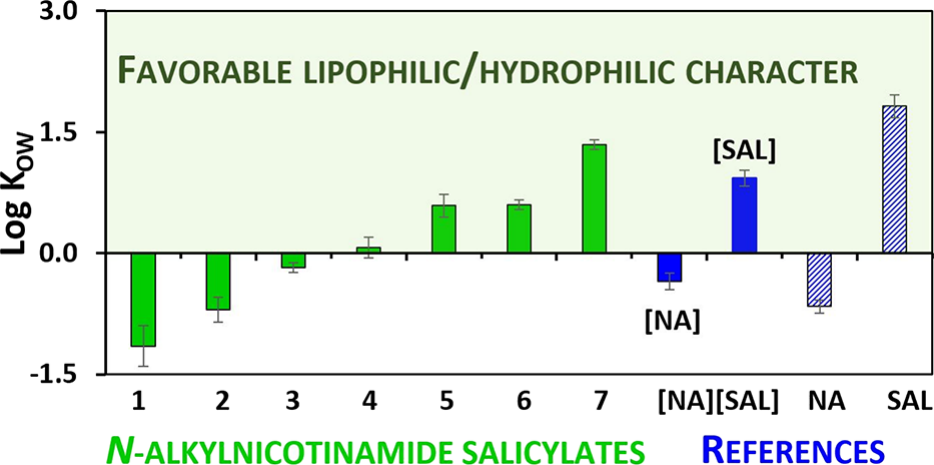
Octanol–water partition coefficient for *N*-alkylnicotinamide salicylates and reference substances.

The outermost layer of the skin (stratum corneum) contains around 40% water, a relatively low amount compared to the rest of the human body. Given the hydrophobic nature of 1-octanol, it can be used as a simplified model for the stratum corneum.^50^ The octanol–water partition coefficient (*K*_OW_) value represents the proportion between the concentrations of a substance in both liquid phases of a system composed of water and 1-octanol. When log *K*_OW_ is equal to 0, it signifies that the compound has equal affinity to both organic and aqueous environments, albeit the desirable log *K*_OW_ values for substances administered dermally are in the range between 1 and 3.^46,51^ The log *K*_OW_ values are given in Figure 6 (exact values are provided in Table S4, Supporting Information).

Given the determined lipophilicity of *N*-alkylnicotinamide salicylates, it can be concluded that three compounds with the shortest alkyl substituents (**1**–**3**) may not be suitable candidates for topical formulations. The negative log *K*_OW_ values indicate that they are more soluble in aqueous media; therefore, when applied to skin, they may not be able to penetrate the lipophilic stratum corneum effectively. However, it should be noted that a similar characteristic can also be attributed to **NA**, which is a widely used ingredient in skin care products. An attention should also be paid to an interesting phenomenon occurring in the case of [**NA**][**SAL**], where each of its components exhibits different affinities for octanol and water due to the lack of ionic bonds in its structure. This observation points to the possibility of using cocrystals as innovative medicinal ingredients with versatile action profiles. Among the most frequently used topical acne agents are clindamycin, azelaic acid, and also **SA**, analyzed in the course of this research.^52−54^ The values of their log *K*_OW_ are 1.60, 1.42, and 1.82, respectively, being within the desired range (1–3) and thus proving its validity.

### 3.4 Skin Permeability Coefficient

The development of mathematical models to describe and predict skin permeability has been an area of active research. They are a key tool in the study of pharmacokinetics, facilitating the design of drugs and cosmetics. These models can be categorized into quantitative structure–permeability relationships (QSPRs), expressions based on diffusion mechanisms, or a combination of both. They can be a useful tool for preliminary studies of dermally applied drugs and as an alternative to the oft-used Franz chamber or studies on living organisms, which can be time-consuming and financially demanding.^55^ Three mathematical models were used in the course of this study that correlate skin permeability coefficient (Kp) of a drug in aqueous solution with solute molecular weight (MW) and logarithm of octanol–water partition coefficient (log *K*_OW_), namely, Frasch, Potts and Guy, and modified Robinson model.^56−58^

Gathered data (presented in Table S5 in the Supporting Information) show that, as the alkyl chain length increases in *N*-alkylnicotinamide salicylates (**1**–**7**), there is a corresponding rise in the skin permeation rate, indicating that increasing hydrophobicity leads to enhanced permeability through biological membranes. However, these values differ dramatically from the ones obtained for the reference compounds by several (**NA**) or even several thousand-fold, as can be seen most evidently by comparing them with the data for **SAL**. Given that two octanol–water partition coefficient values were obtained for [**NA**][**SAL**], two separate Kp values were also determined, revealing a similar discrepancy in values. It is notable, however, that **SAL** contained in the cocrystal significantly decreased its intensity of permeation through the skin layers, varying in this aspect much more than **NA**. The desired skin permeability coefficient for dermal formulations can vary depending on the specific drug or active ingredient being delivered, as well as the desired therapeutic effect. In general, however, the most desirable is Kp > 5 × 10^–3^. This requirement is not met by any of the compounds tested, but the closest to the desired value was noted for pure **SAL**.^59^

**Figure 7:**
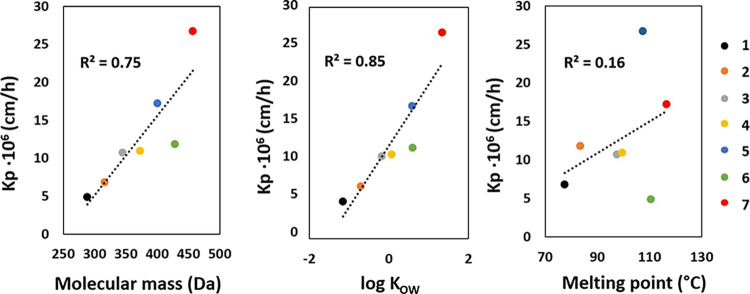
Correlation between skin permeability coefficient (Kp) and molecular mass, assessed log *K*_OW_, and melting point for *N*-alkylnicotinamide salicylates (**1**–**7**).

Correlation of the physicochemical parameters with the skin permeation coefficient values, determined with the Potts and Guy model, as it is the most popular and accurate mathematical model, showed that the biggest detereminating factor is the octanol–water permeation coefficient with *R*^*2*^ = 0.85 (Figure 7). The molar mass still has a significant effect (*R*^*2*^ = 0.75), while the melting point apparently does not determine the rate of skin penetration in any way (*R*^*2*^ = 0.16). These data support the utilization of log *K*_OW_ values as a key parameter in predicting the permeation through the skin and simultaneously reduce the relevance of other factors, such as the temperature of melting during development of new active substances administered epidermally.

### 3.5 Solubility in PBS

**Figure 8:**
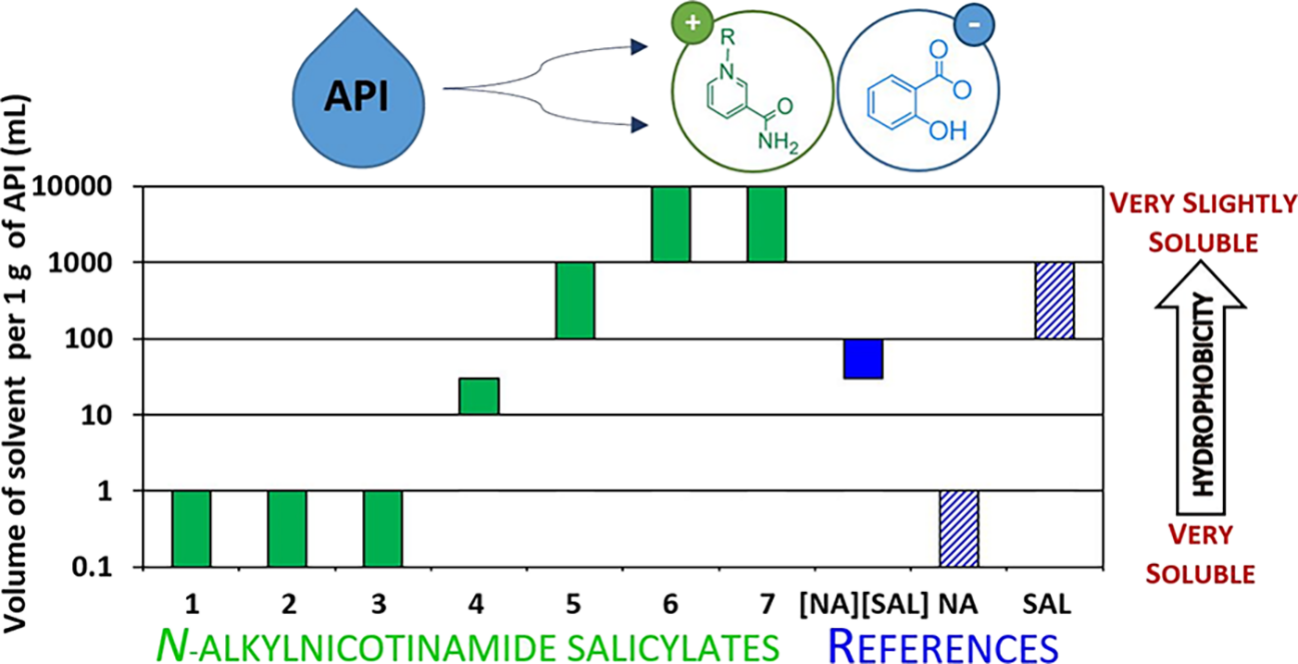
Solubility in PBS for *N*-alkylnicotinamide salicylates (**1**–**7**) and reference substances.

Ensuring that the active ingredients are in a form that allows them to penetrate the epidermal layer and reach the tissue, where they can exert their therapeutic effect, is one of the key challenges in the development of novel topical drugs. Most studies on the solubility of novel APIs are carried out with pure water, aqueous PBS solutions were much more preferred due to their higher similarity to intra- and intercellular media in epidermis, while envisaging their use in transdermal delivery and skin care applications.^60^ The solubility of the obtained APIs was characterized using the method recommended by the Pharmacopeia,^61^ which distinguishes individual solubility classes from “Very Soluble” (<1 mL g^–1^) to “Practically Insoluble” (>10,000 mL g^–1^). The collected data are presented in Figure 8 (as well as in Table S6 in the Supporting Information).

As expected, the alkyl chain extension from ethyl (**1**) to tetradecyl (**7**) contributed to a significant reduction of products’ affinity to PBS. Thus, only **1**–**3** could be classified as “Very Soluble”, whereas **4** and **5** were established to be “Soluble” and “Slightly Soluble”, respectively. Salts with the longest chains (**6** and **7**) were assessed as “Very Slightly Soluble”, which means that their solubility in PBS was even lower in comparison to all applied references. Intriguingly, while **NA** and **SAL** possessed diverse affinity to PBS and were classified as “Very Soluble” and “Slightly Soluble”, respectively, their cocrystal [**NA**][**SAL**] was found to be “Sparingly Soluble”. This means that, in the case of this cocrystal, some specific interactions between both APIs must occur, and in effect, its hydrophobicity is similar to salt **4** comprising an octyl chain. The collected results revealed that absorption in the deeper layers of epidermis will plausibly be highest for **1**–**3** (at the **NA** level); however, the more hydrophobic compounds (**5**–**7**) should have greater affinity to the outer layers of the seborrheic skin. Thus, within the homologous series, it is possible to select the optimum length of the alkyl chain in terms of API penetration, ultimately leading to enhancement of the therapeutic effect.

### 3.6 Antimicrobial Activity

To assess the effectiveness of the analyzed compounds in treating acne vulgaris, their activity against various bacterial strains was evaluated. Studies have shown that both *C. acnes* and *S. epidermidis* occur within acne lesions in large quantities.^62^*C. albicans* is the most commonly responsible for symptomatic skin infections. Among other things, it causes hyperkeratosis and erythema, often associated with acne lesions.^63^*P. aeruginosa* can lead to folliculitis, exhibiting symptoms similar to acne that can appear anywhere on the body.^64^*S. aureus*, the most abundant component of the skin microbiota, can act as a pathogen in a plethora of skin infections. It should be also noted that its presence alongside other microbes in acne lesions has been documented.^65^ However, particular attention should be paid to *C. acnes*, which is recognized as a main pathogenic factor in acne vulgaris.^66^

The obtained minimum inhibitory concentration (MIC) and minimal bactericidal/fungicidal concentration (MBC/MFC) values are listed in Table 2. It was revealed that only three compounds with the longest alkyl chains (**5**–**7**) as well as one of the reference compounds—**SAL** possess high potential in inhibiting development of the *C. acnes* strain. Noteworthy, the salts with shorter chains (**1**–**4**) showed biological activity only against *S. epidermidis* and *S. aureus*, although they proved to exhibit only bacteriostatic properties, and the MBC value could not be determined for them at concentrations reaching even 0.4% (4096 mg/L).

**Table 2:** Antimicrobial Activity for *N*-Alkylnicotinamide Salicylates (**1**–**7**) and Reference Substances: [**NA**][**SAL**], **NA**, and **SAL**, Calculated as MIC and MBC/MFC

	**strain**									
	C. acnes		S. epidermidis		C. albicans		P. aeruginosa		S. aureus	
**no**	**MIC**^a^	**MBC**^a^	**MIC**^a^	**MBC**^a^	**MIC**^a^	**MFC**^a^	**MIC**^a^	**MBC**^a^	**MIC**^a^	**MBC**^a^
**1**	b	b	4096	b	b	b	b	b	1024	b
**2**	b	b	2048	b	b	b	b	b	1024	b
**3**	b	b	4096	b	b	b	b	b	2048	b
**4**	b	b	2048	b	b	b	b	b	1024	b
**5**	1024	1024	256	256	b	b	1024	b	256	256
**6**	64	64	256	256	256	256	256	256	8	8
**7**	4	4	4	4	16	16	64	64	0.5	0.5
**[NA][SAL]**	b	b	2048	2048	2048	b	2048	b	2048	2048
**NA**	b	b	b	b	b	b	b	b	b	b
**SAL**	1024	4096	1024	1024	2048	2048	2048	4096	1024	1024

a In mg/L.

b MIC/MBC/MFC out of range.

Interestingly, the cocrystal [**NA**][**SAL**] did not show bactericidal activity against *C. acnes*, but its influence on the development of other microorganisms may be observed. However, this activity seems to originate only from the salicylic moiety (most MIC/MBC results are twice as high). Intriguingly, **NA** turned out to be ineffective toward each of the strains used in the study at concentrations of 0.4%. These results stay in contradiction to the previous reports where **NA** was described as effective in inhibiting not only the growth of acne-causing bacteria species but also other tested strains.^67,68^ On the contrary, **SAL** is recommended at doses reaching 2%, which, according to gathered data, are sufficient to achieve the therapeutic effect.

Generally, the greater length of the alkyl chain was found to improve the biological activity.^69,70^ It is consistent with the available literature, although it may also indicate that the presence of the alkyl chain of the appropriate length in QASs is more important than the used building blocks (**NA** and **SAL**). Nevertheless, it should be emphasized that many synthetic QASs are known for their high toxicity and may be detrimental to the human body. Namely, didecyldimethylammonium chloride was found to be dermal irritant and can be acutely toxic. Therefore, their analogs obtained from raw materials of natural origin constitute a great alternative for the pharmaceutical industry.^71^ Fascinatingly, simple structural modification of APIs by introducing an alkyl chain to the cation can lead to a broadening of the spectrum of their biological activity and improving it by 200 to even 2000 times, compared to that of **SAL** alone. This means that it is possible to significantly reduce the dose of API while simultaneously increasing the effectiveness of therapy. In addition, the results of biological activity studies are also consistent with the results of surface activity and longer alkyl chain length improves both parameters. Consequently, compounds with the longest alkyl chains exhibit better wetting properties, which facilitates contact of the active substance with the skin surface as well as the cell membranes of targeted bacteria.

### 3.7 Contact Angle of Aqueous Solution of API

A larger contact area between the drug and skin is crucial in acne therapy as it allows more effective penetration of the active ingredients, which can accelerate the reduction of acne lesions. In the case of seborrheic skin, which occurs among many people affected by acne, the epidermis is covered by a hydrophobic lipid layer, which impedes the penetration of active substances, especially those that are well soluble in water. In addition, ensuring proper drug absorption through the skin can also reduce the risk of side effects and increase the effectiveness of therapy.^72^ The addition of surfactants, which improve the wettability of hydrophobic surfaces, can be a solution to this problem.^73,74^ Keeping this in mind, instead of adding a surfactant, we adopted the strategy of introducing an alkyl chain into the **NA** molecule, which resulted in the occurrence of the desired surface-active properties in the obtained APIs. Wettability analyses of aqueous solutions of the new APIs were carried out on paraffin, whose hydrophobicity is similar to that of the human skin.^75^ Experiments were performed at concentrations corresponding to common **NA** and **SAL** contents in commercial formulations (0.25–1.00%).^67,76,77^ Due to low solubilities of **5**–**7**, tests for these salts were performed at concentrations of 0.25, 0.03, and 0.01%, respectively (please see Figure S43 in the Supporting Information). The concentrations for **6** and **7** were selected on the basis of their effective dose estimated after analysis of MIC/MBC tests (please see data in Table 2).

**Figure 9:**
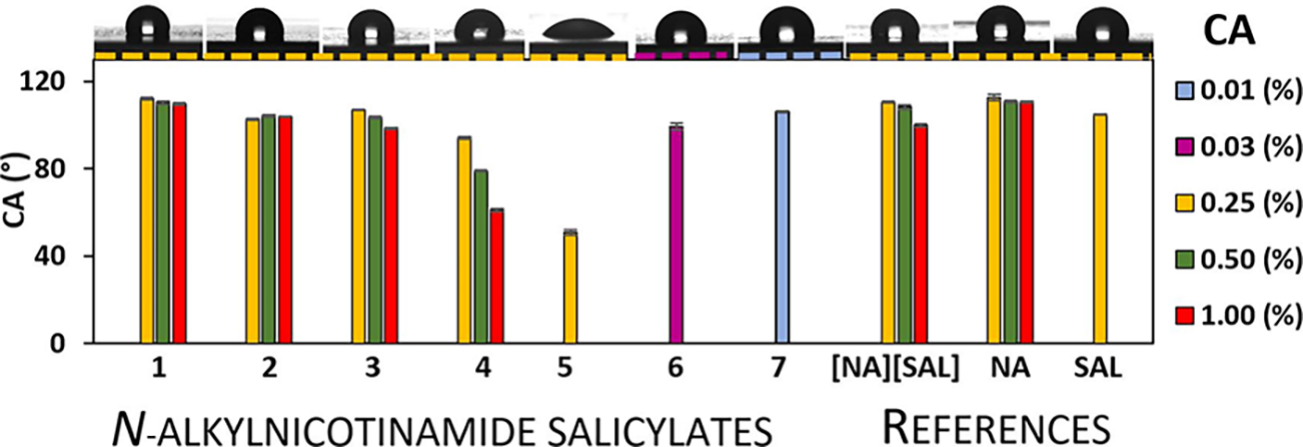
Contact angle (CA) of aqueous solutions of *N*-alkylnicotinamide salicylates (**1**–**7**) and reference substances. Shapes of droplets are presented at the lowest available concentrations for each compound.

It is evident from the data in Figure 9 (and in Table S7 in the Supporting Information) that both the reference substances (**NA**, **SAL**, and [**NA**][**SAL**]) and products **1**–**3** containing the shortest alkyl chains (from C_2_ to C_6_) do not show surface activities. As a result, the contact angle (CA) of their solutions at concentrations ranging from 0.25 to even 1.00% is in the range of 99–112° and is close to that of pure water (113°).^78^ Interestingly, **SAL** itself did not solubilize at concentrations higher than 0.25%, but its cocrystal solubilized even at a concentration of 1.00%, for which the wetting angle was equal to 100°. As expected, the increase in the alkyl chain length resulted in an enhancement of the amphiphilic character of the salts, resulting in an increase in their surface activity as well. Thus, an increase in concentration for **4** from 0.25 to 1.00% resulted in a decrease in CA from 94 to 61°. For compound **5**, containing a decyl substituent, the highest CA value (51°) was reached at a concentration of 0.25%. It should also be noted that compounds **6** and **7** at significantly lower concentrations (<0.05%) show improved wettability of hydrophobic surfaces compared to **NA** even at 1.00%. This means that compounds **5**–**7** may significantly improve the contact area between the drug and inflamed skin, which is likely to be a substantial factor in accelerating the therapeutic effect.

### 3.8 Molecular Docking

Acne treatment primarily addresses two contexts. The first is to eliminate the main cause of skin lesions, which stems from pathogenic microorganisms. Consequently, the primary goal of these drugs is to minimize or prevent the formation of future skin lesions. The second aspect of acne therapy involves alleviating symptoms associated with acne, such as painful inflammation that reduces the quality of life. Therefore, anti-inflammatory activity is crucial in acne treatment to alleviate these symptoms and enhance the overall well-being for those affected. To assess the potential anti-inflammatory effects of the synthesized products, molecular docking simulations have been performed.

**Figure 10:**
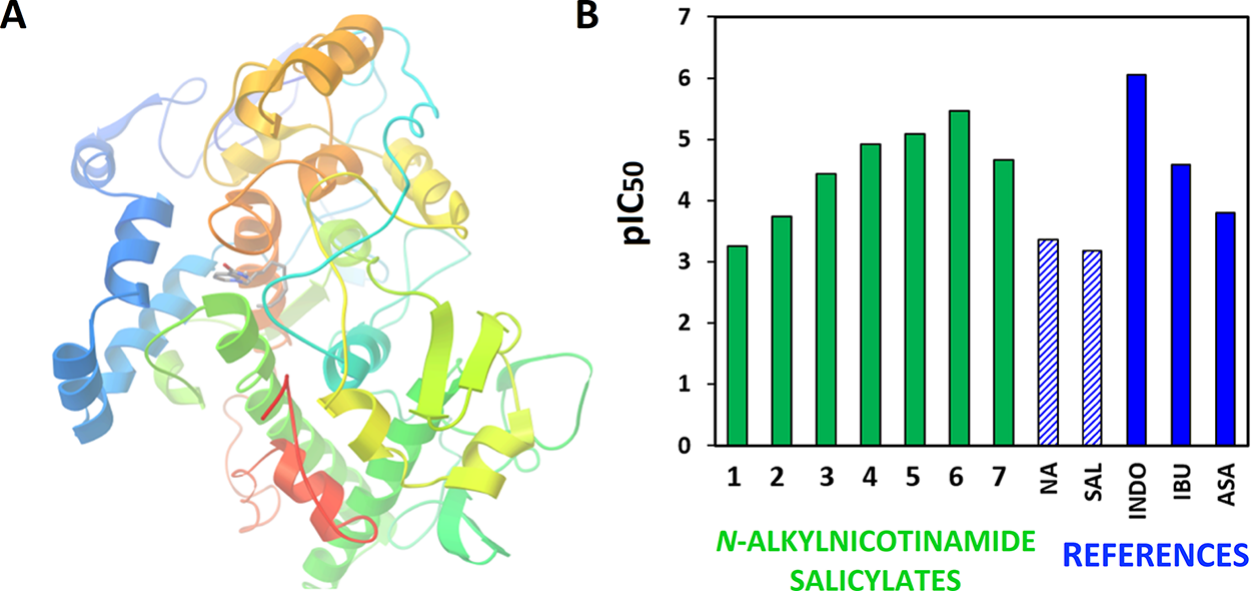
Structure of the *N*-decylnicotinamide cation (**5**) and the cyclooxygenase-2 (PDB ID: 1CX2) enzyme complex (A) and the relationship between the ligand structure and activity expressed as an inhibition constant of examined ligands, for *N*-alkylnicotinamide salicylates (**1**–**7**), nicotinamide (**NA**), salicylic acid (**SAL**), indometacine (**INDO**), ibuprofen (**IBU**), and acetylsalicylic acid (**ASA**) (B).

Structure-based drug design (SBDD) stands as a predominant approach in drug discovery when the precise structure of the biological target is known.^79^ As shown in Figure 10A, docking simulations were conducted on the binding pocket of cyclooxygenase-2 (COX-2, PDB ID: 1CX2). The cyclooxygenases are involved in the conversion of arachidonic acid to prostaglandins, which plays a key role in the generation of the inflammatory response.^80^ Therefore, COX-1 and COX-2 are the pharmacological targets of nonsteroidal anti-inflammatory drugs (NSAIDs) and have been the subject of many molecular docking investigations.^81−83^ As reference compounds, ligands with established anti-inflammatory activity (indomethacin, ibuprofen, aspirin, and **SAL**) were selected and compared with the results obtained from docking simulations of **NA** and its alkylated derivatives—cations of salts **1**–**7**.

While **NA** and **SAL** have been reported to display anti-inflammatory activity,^84,85^ it remains uncertain whether alkylation of nicotinamide enhances or diminishes these properties. Analysis of the docking simulations, provided in Figure 10B (and in Table S8 in the Supporting Information), led to the conclusion that, initially, the activity slightly decreases with IC_50_ values of **NA** and its ethylated analog **1** being 428 and 559 μM, respectively (corresponding DS −4.59 and −4.44 kcal mol^–1^). However, as the alkyl length increases, the activity begins to rise, ultimately reaching an optimum for the cation with a dodecyl alkyl chain (**6**, IC_50_ = 3.39 μM, DS = −7.46 kcal mol^–1^), which is 125 times more potent than **NA** itself. It is noteworthy that, according to calculations, cations **4**–**6** exhibited greater potency than commonly used NSAID drugs such as aspirin (IC_50_ = 160 μM, DS = −5.18 kcal mol^–1^) and ibuprofen (IC_50_ = 25.5 μM, DS = −6.27 kcal mol^–1^), although they were less potent than indomethacin (IC_50_ = 0.892 μM, DS = −8.25 kcal mol^–1^), which has commonly been used for decades to relieve pain, swelling, and joint stiffness.

These arguments are consistent with recent findings from *in silico* studies of amphiphilic compounds with alkyl chains of 5–13 carbon atoms (medium chain monoglycerides),^86^ considered as possible anti-inflammatory agents and docked to the COX-2 protein (with binding affinities ranging from −7.58 to −7.02 kcal mol^–1^). Interestingly, these compounds were also compared to aspirin and ibuprofen as reference drugs (with binding affinities of −7.14 and −6.76 kcal mol^–1^, respectively). The conclusions drawn from these investigations support our findings, suggesting that ionic compounds with alkyl chains providing amphiphilic properties can act as COX-2 inhibitors on a level comparable to that of common NSAID drugs. The observed discrepancies between the obtained results and those of the referenced study are reasonable, considering differences in the crystal structure of the protein and algorithms used.

**Figure 11:**
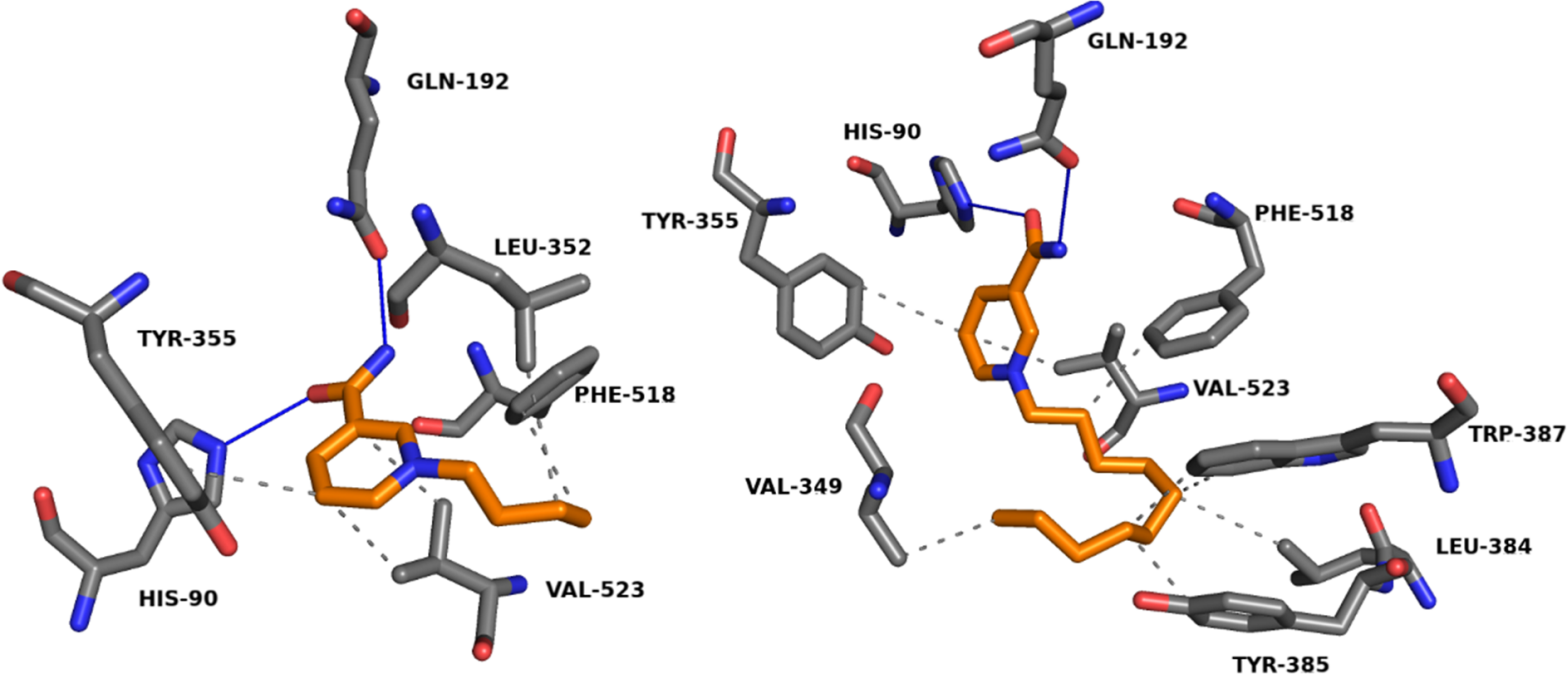
Docked poses of *N*-butylnicotinamide (**2**, binding energy −5.10 kcal mol^–1^) and *N*-decylnicotinamide (**5**, binding energy −6.95 kcal mol^–1^) cations with the active site region of the cyclooxygenase-2 (PDB ID: 1CX2) enzyme. Hydrogen bonds are indicated by blue lines; hydrophobic interactions are indicated by gray dotted lines. The ligands are highlighted in orange.

Hydrophobic interactions and hydrogen bonds were observed in the case of all ligands and COX-2 complexes during analysis by PLIP. Examples of formed complexes for **2** and **5** are demonstrated in Figure 11 (all 3D docking poses are available Figures S44–S56 in the Supporting Information). In the case of **NA** and its derivatives, hydrogen bonds were formed exclusively by the amide group (−CONH_2_), while pyridinium and *N*-alkylpyridinium moieties were responsible for the hydrophobic interactions. Therefore, modification of alkyl chain can be used to improve binding of the ligand to the receptor. However, the beneficial effect of such change depends on the specificity of the molecular pocket and mutual interactions. All complexes were analyzed via PLIP, and the possible interaction with the receptor is listed and visualized in Tables S9–S21 in the Supporting Information.

### 3.9 Ecotoxicity toward Aquatic Life

*A. franciscana* is a model representative of crustaceans representing marine zooplankton. Its significance within the biosphere is essential, as it ensures the proper functioning of aqueous ecosystems. Many organizations such as the OECD, US EPA, and ONZ consider the EC_50_ parameter assessed on crustaceans as a characteristic value, corresponding to their potential impact on the hydrosphere. In recent years, scientists have drawn attention to the fact that, through improper disposal or uncontrolled release from wastewater, many active substances can easily enter the environment. Unfortunately, the majority of such chemicals are known to disrupt the biological balance in aquatic and soil ecosystems. As a result of water or soil pollution, there is a potential risk of changes in the reproduction and development of various food chain representatives caused by biologically active substances improperly released into the environment.

It has been evidenced that parasiticides show harmful effects on nontarget organisms, e.g., on dung insects, aquatic invertebrates, protozoa, worms in soil, and surface water. Some antibiotics were assessed to have harmful effects on algae and plants.^87^ Therefore, there is an urgent need to control and reduce the impact of pharmaceuticals on the biosphere. It should also be noted that toxicity to aquatic organisms is often used as an indicator of potential toxicity to humans, as many toxic mechanisms are common across different species. However, differences in metabolism, exposure pathways, and resistance among species can affect the degree of toxicity. Therefore, results from tests on aquatic organisms are typically a preliminary step in assessing human risk and require further studies of higher organisms. In this context, toxicity analyses for newly obtained APIs are recommended by different governments and regulatory agencies worldwide as early as the preclinical research stage.^88^ Ecotoxicity data toward *A. franciscana* for products **1**–**7** as well as reference substances ([**NA**][**SAL**], **NA**, and **SAL**) are listed in the Table 3.

**Table 3:**
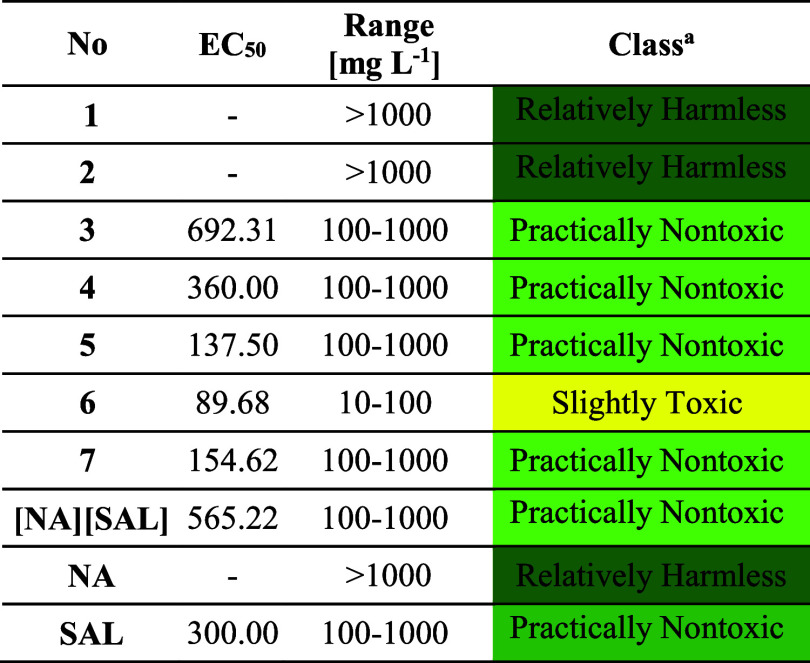
Ecotoxicity toward *A. franciscana* for *N*-Alkylnicotinamide Salicylates (**1**–**7**), **[NA][SAL]**, **NA**, and **SAL**^a^

a According to the acute toxicity rating scale by Fish and Wildlife Service.^88^

The performed ecotoxicity studies revealed that the synthesized forms of **NA** and **SAL** do not pose a severe threat to the environment, regardless of the length of the alkyl chain in the cation. Thus, products **1** and **2** along with **NA** were classified as “Relatively Harmless” with toxicity range above 1000 mg L^–1^. Interestingly, the other products were established as “Practically Nontoxic”, except **6** (with dodecyl chain) that was assessed only as “Slightly Toxic” toward *A. franciscana*. Therefore, one can conclude that, after achieving the maximum toxicity, there is a “cutoff effect” caused by a significant rise in hydrophobicity of a substance due to alkyl elongation.^69^ It is noteworthy that a majority of other QASs have been recognized as substantially toxic to aquatic life, which is considered as their greatest weakness in the case of commercial use. For example, salts with popular cations like cholinium or 1-alkyl-3-methylimidazolium may be responsible for the “Moderately Toxic” or even “Highly Toxic” level, with EC_50_ in the range of 1–10 and 0.1–1 mg L^–1^, respectively.^89,90^

## 4 Conclusions

In this research, salicylic acid and nicotinamide (vitamin B_3_) were used for the development of new Active Pharmaceutical Ingredients (APIs) with attractive application potential in the treatment of acne vulgaris. Thus, an efficient and sustainable approach using well-known, cost-effective, and commercially available reagents derived from materials of natural origin was elaborated, which allowed for a significant reduction in the environmental impact according to the calculated *Green Chemistry* metrics. Among *N*-alkylnicotinamide salicylates, compounds with longer alkyl substituents (**4**–**7**) are suitable candidates as novel APIs for topical formulations according to the assessed octanol–water partition coefficients. The solubility tests revealed that the more hydrophobic compounds (**5**–**7**) had a greater ability to penetrate the stratum corneum of the seborrheic skin, while **1**–**3** as well as nicotinamide were characterized by facilitated penetration through the deeper layers of epidermis. These observations are consistent with the assessed contact angles of their aqueous solutions, indicating favorable wettability of hydrophobic surfaces by compounds **5**–**7** demonstrating significant improvement of the contact area between the drug and inflamed skin. Interestingly, designed new APIs exhibited excellent antimicrobial activity toward pathogens associated with acne (e.g., *C. acnes* and *S. epidermidis*). The activity of compounds **5**–**7** against the tested microorganisms was higher by as much as up to 2000 times compared to nicotinamide or salicylic acid alone. This means that the conscious design of new drugs according to our strategy paves a path to a significant reduction of their effective dose. According to the performed molecular docking studies, the elongation of the alkyl chain can improve binding to the receptor. In effect, **4**–**7** exhibited greater potency than commonly used NSAID drugs such as aspirin. In addition, the conducted ecotoxicity studies showed that, regardless of the length of the alkyl, none of the products poses a serious threat to the environment. Thus, gathered data indicate that the strategy of developing new QASs through appropriate modification and combining ionic APIs has proven to be an effective tool for creating more potent and safer drugs that adhere to the concept of sustainable pharmaceutical development and biodiversity protection.

## Supporting Information Available

Nicotinamide quaternization reactions, NMR, UV, FTIR spectra, water and bromide ions content, Green Chemistry Metrics, molecular masses of products, octanol–water partition coefficient values, skin permeability coefficient values, solubility in PBS, contact angle studies, binding energies and inhibition constant of ligands, docked poses, and interactions with residues according to PLIP (PDF)
