# Supplementary material for: Unraveling the Potential of Vitamin B3-Derived Salts with a Salicylate Anion as Dermal Active Agents for Acne Treatment
Source: Mol Pharm. 2024 Aug 14;21(9):4634–47. doi: 10.1021/acs.molpharmaceut.4c00543 (PMC11372839; doi:10.1021/acs.molpharmaceut.4c00543)
Supplement: Supplementary file 1 — mp4c00543_si_001.pdf [file mp4c00543_si_001.pdf]

ELECTRONIC SUPPORTING INFORMATION

Unraveling the potential of vitamin B3-derived salts with salicylate anion as dermal active agents for acne treatment

Adriana Olejniczak[1], Witold Stachowiak[1], Daniel Ziental[2], Jolanta Długaszewska[3], Tomasz Rzemieniecki[1], Marcin Wysokowski[1], Teofil Jesionowski[1], Michał Niemczak[1]*

[1]Faculty of Chemical Technology, Poznan University of Technology, Berdychowo 4, Poznan 60-965, Poland

[2]Chair and Department of Inorganic and Analytical Chemistry, Poznan University of Medical Sciences, Rokietnicka 3, Poznan 60-806, Poland

[3]Chair and Department of Genetics and Pharmaceutical Microbiology, Poznan University of Medical Sciences, Rokietnicka 3, 60-806 Poznan, Poland

*Corresponding author at: Michał Niemczak, Poznan University of Technology, Berdychowo 4, Poznan 60-965, Poland; orcid.org/0000-0002-4364-8267; Phone: +48 616653681; Email: michal.niemczak@put.poznan.pl

Figure S1. **Summary of nicotinamide quaternization reactions with alkyl halides.**

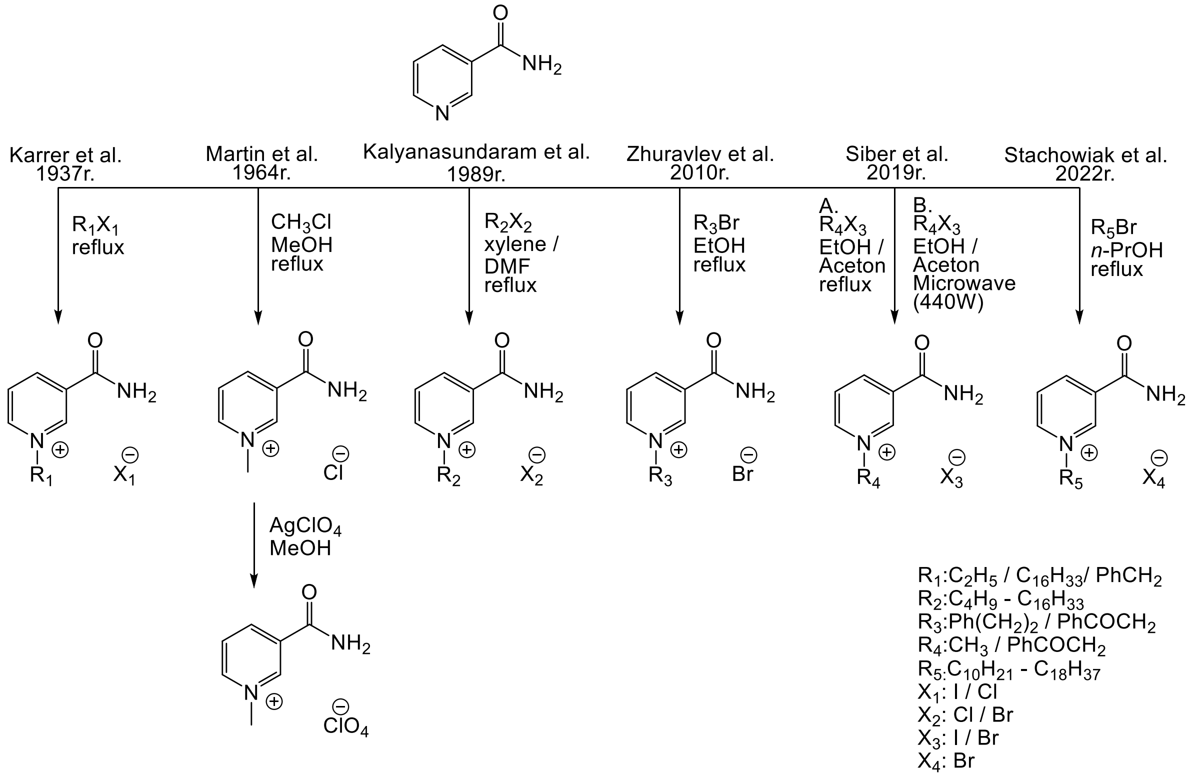

Figure S2. **^{1}H NMR spectrum of *N*-ethylonicotinamide salicylate (1).**

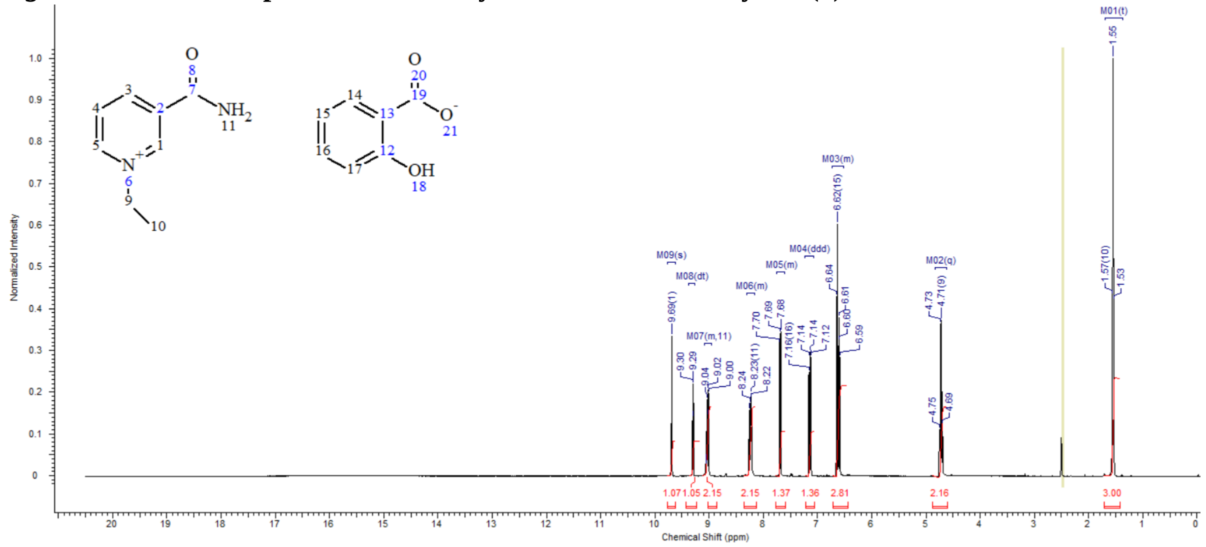

Figure S3. **^{13}C NMR spectrum of *N*-ethylonicotinamide salicylate (1).**

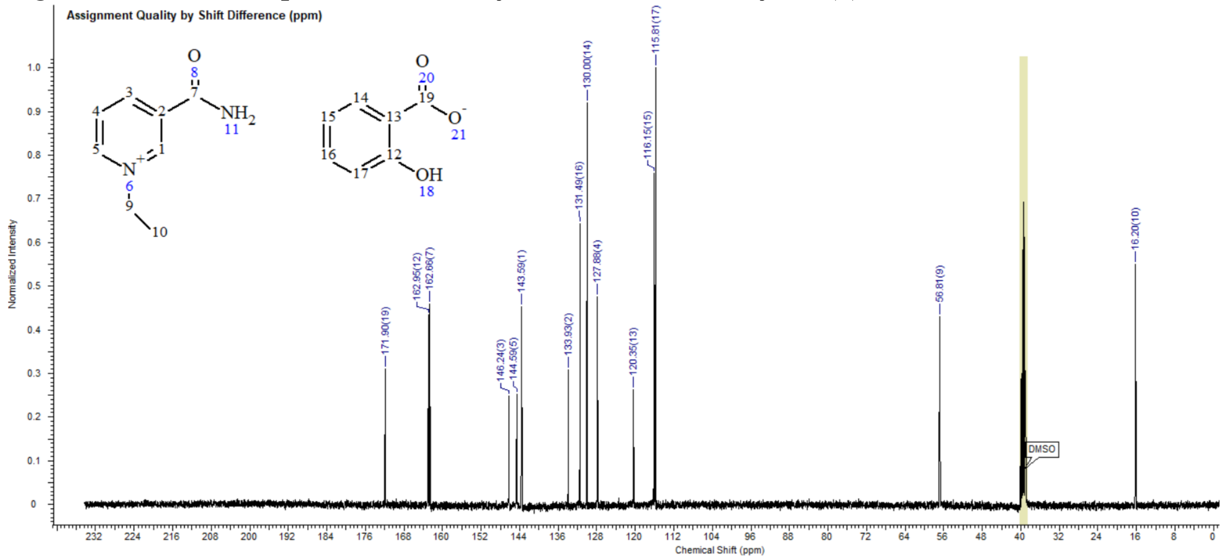

Figure S4. **UV spectrum of *N*-ethylonicotinamide salicylate (1).**

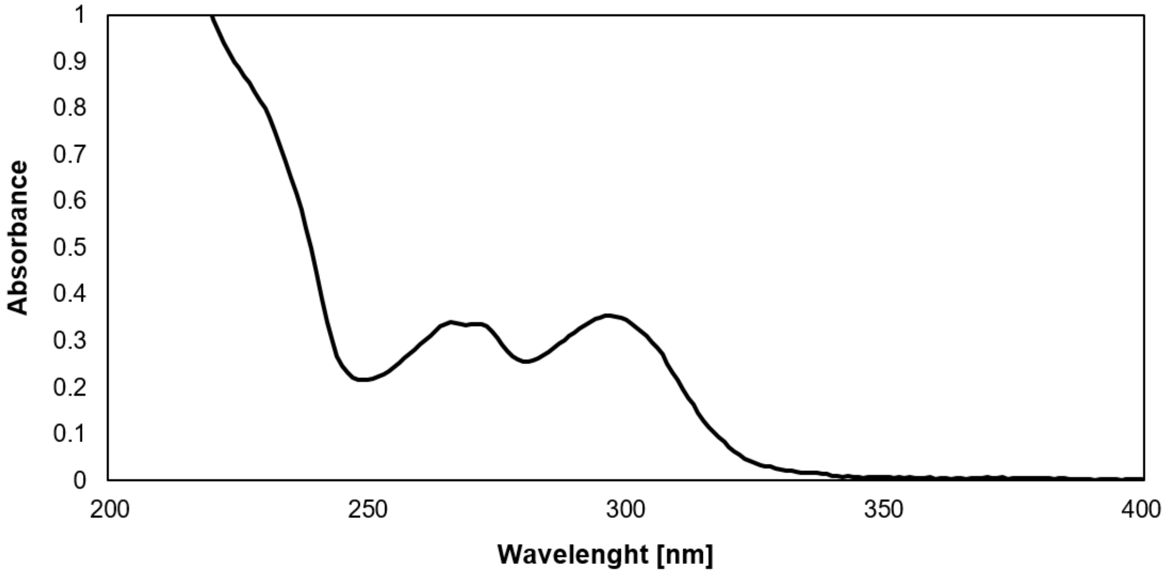

Figure S5. **FT-IR spectrum of *N*-ethylonicotinamide salicylate (1).**

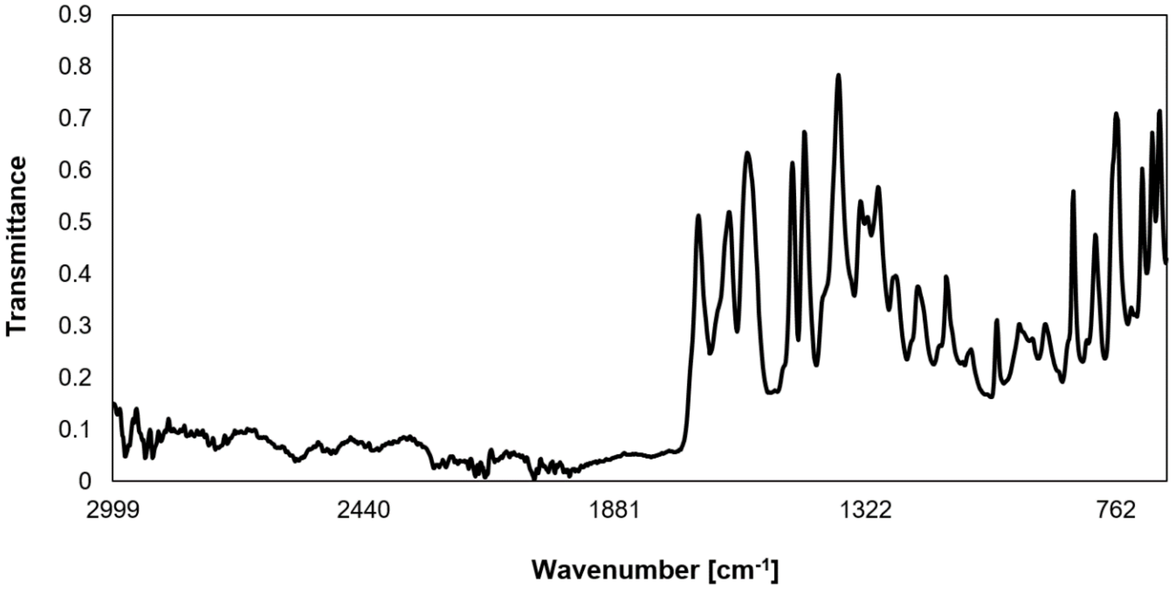

Figure S6. **^{1}H NMR spectrum of *N*-buthylonicotinamide salicylate (2).**

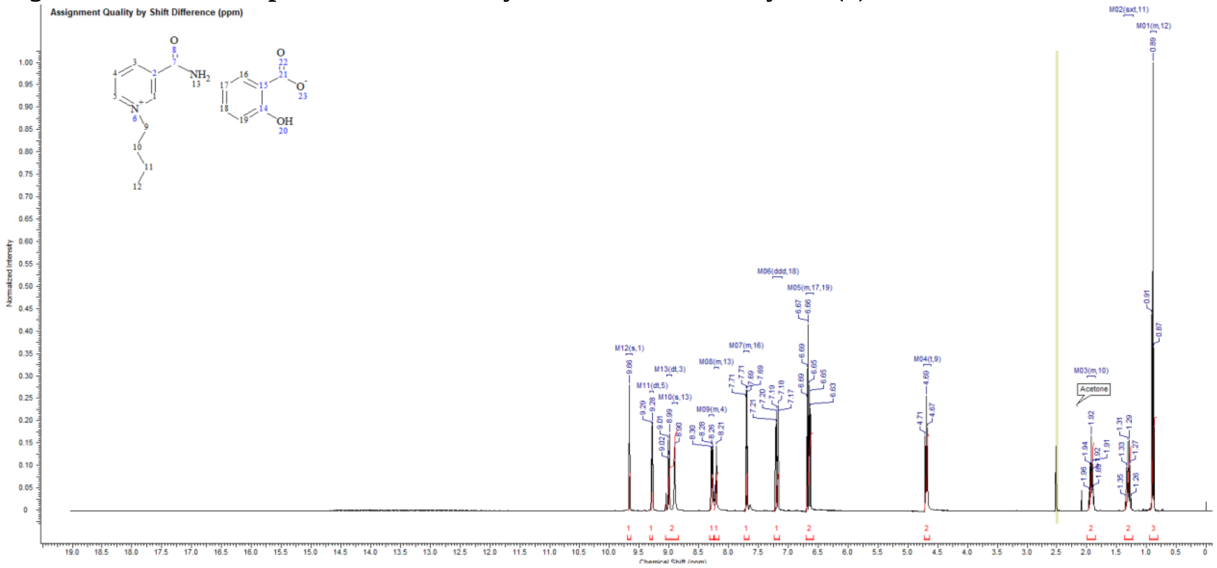

Figure S7. **^{13}C NMR spectrum of *N*-buthylonicotinamide salicylate (2).**

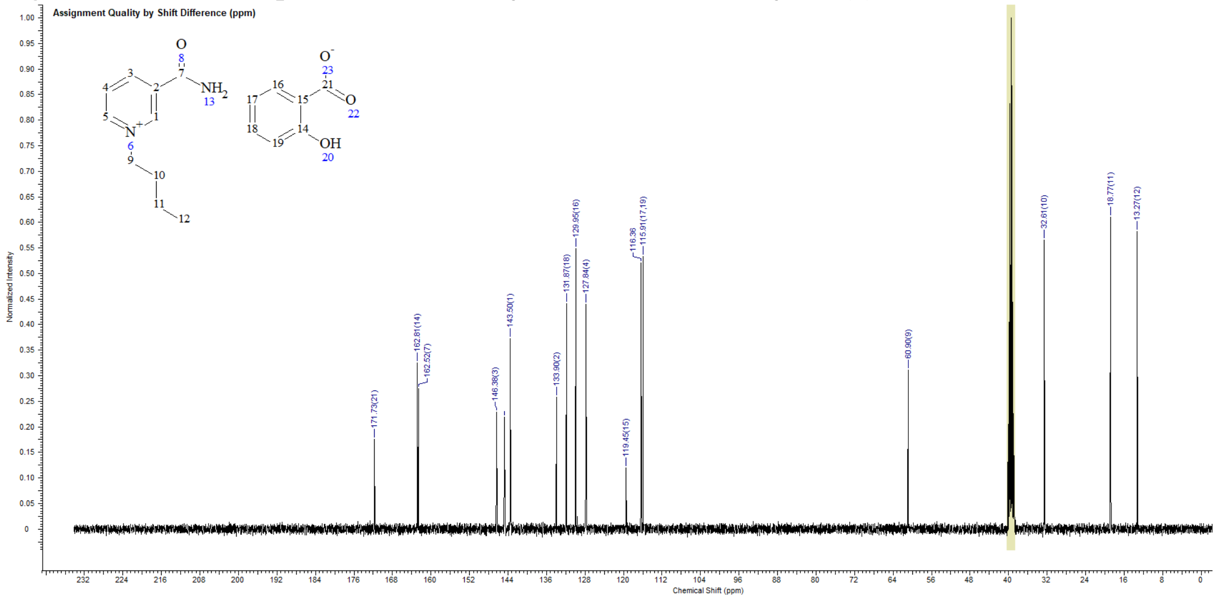

Figure S8. **UV spectrum of *N*-buthylonicotinamide salicylate (2).**

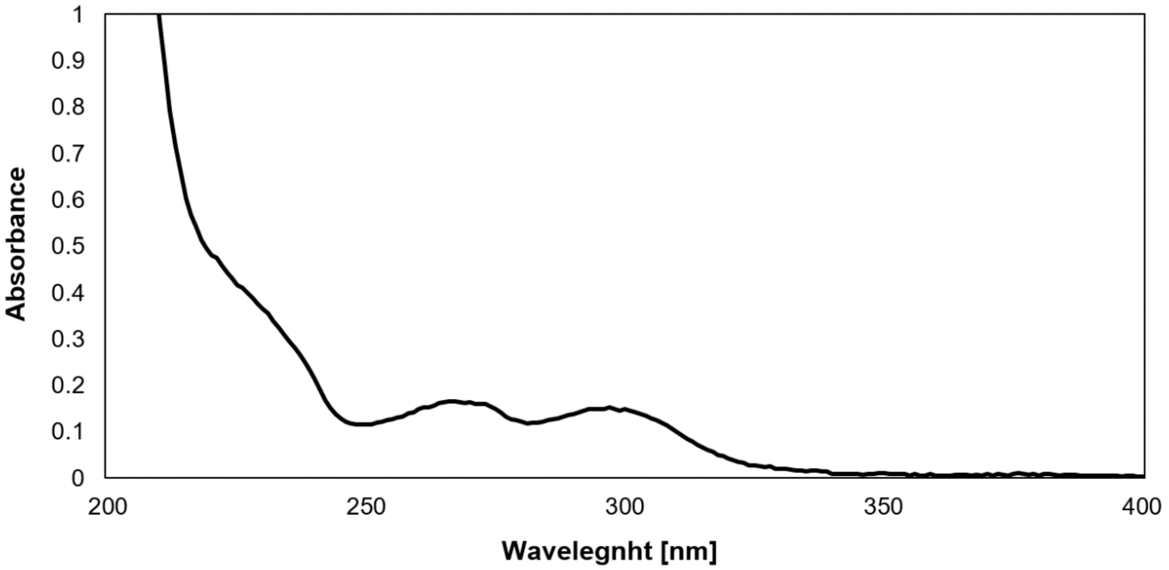

Figure S9. **FT-IR spectrum of *N*-buthylonicotinamide salicylate (2).**

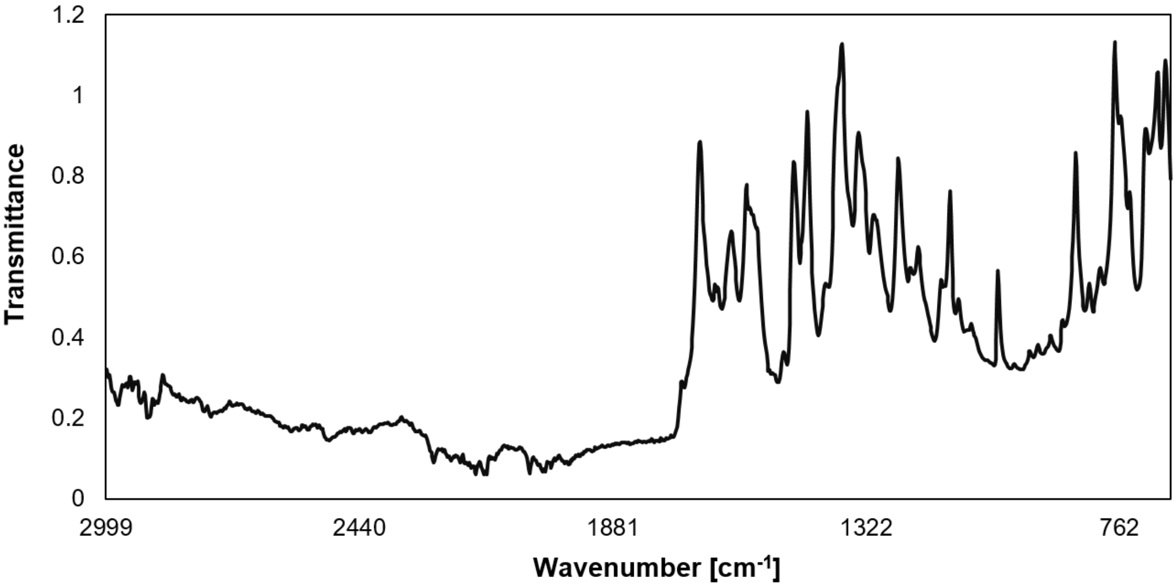

Figure S10. **^{1}H NMR spectrum of *N*-hexylonicotinamide salicylate (3).**

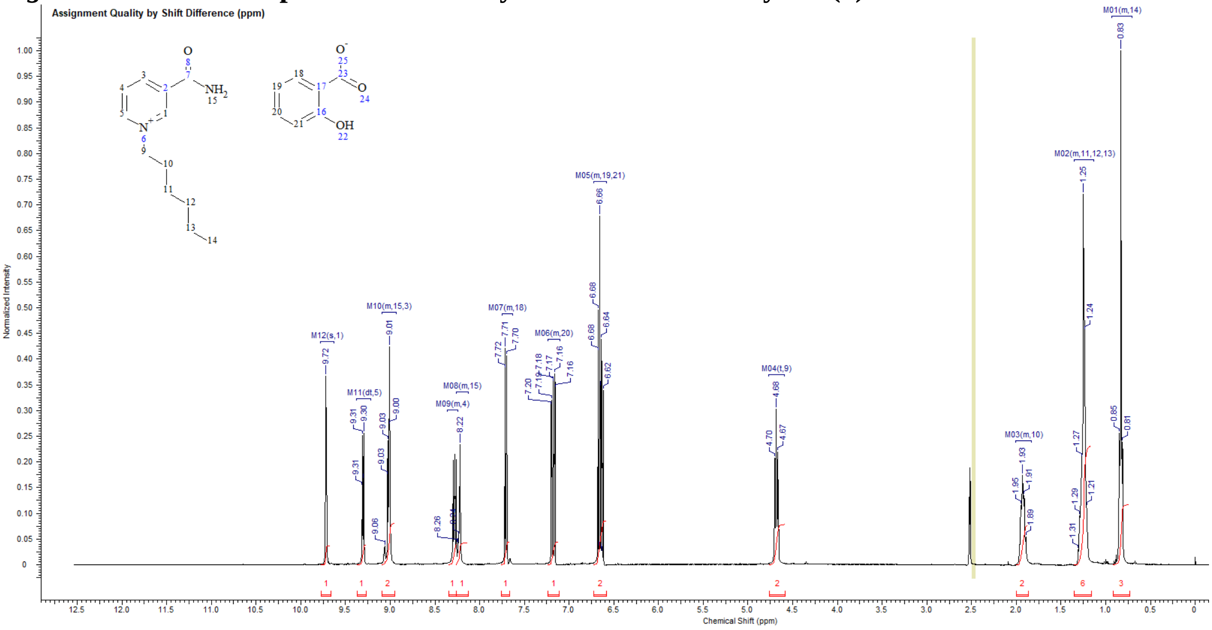

Figure S11. **^{13}C NMR spectrum of *N*-hexylonicotinamide salicylate (3).**

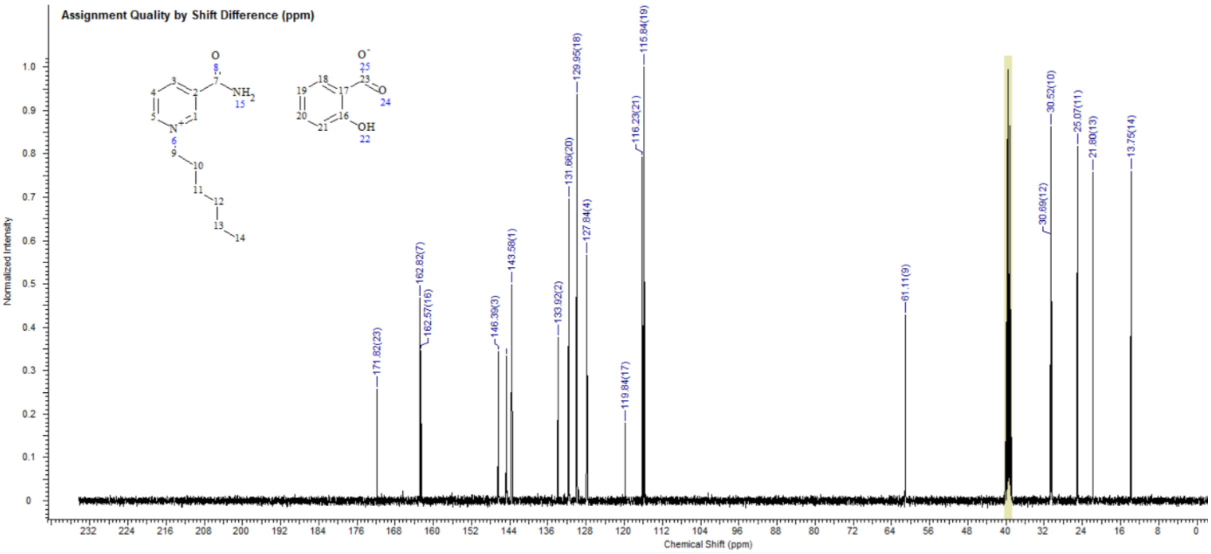

Figure S12. **UV spectrum of *N*-hexylonicotinamide salicylate (3).**

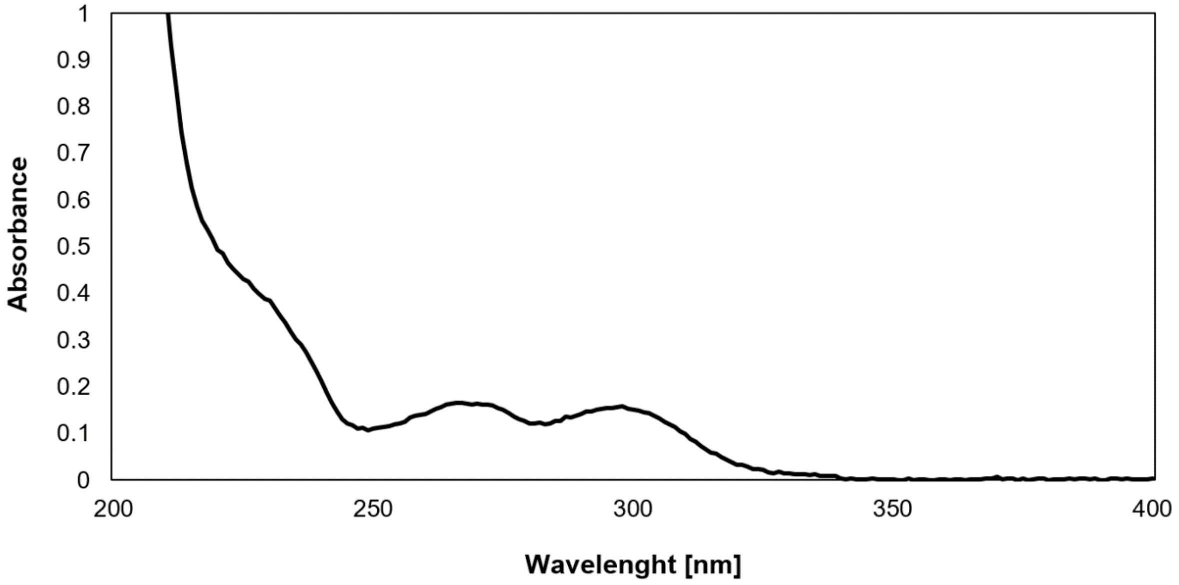

Figure S13. **FT-IR spectrum of *N*-hexylonicotinamide salicylate (3).**

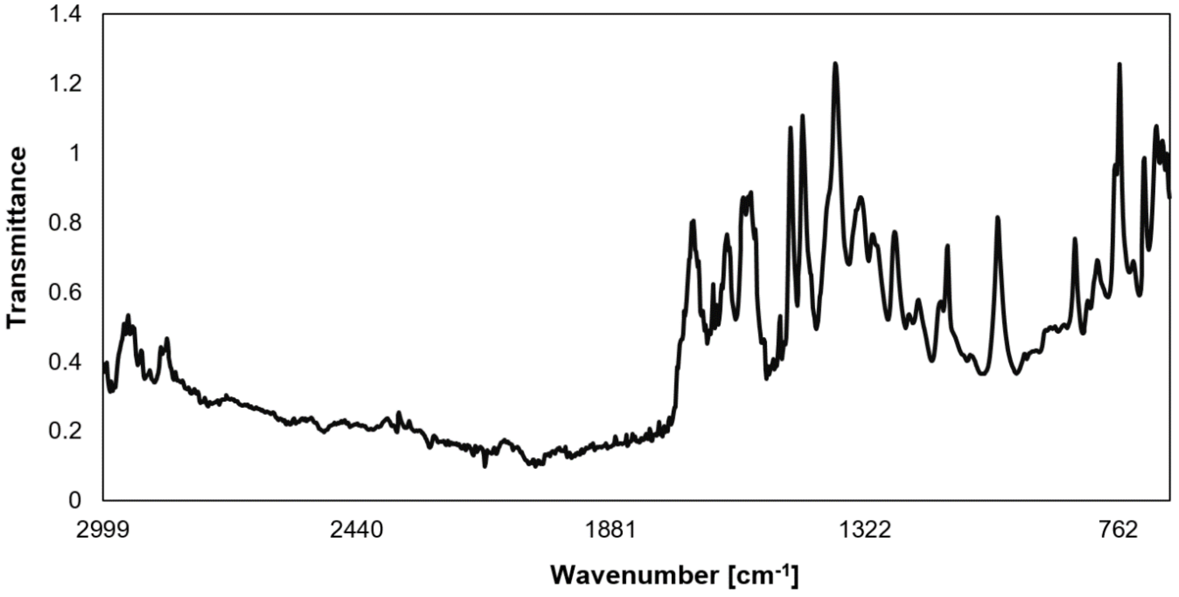

Figure S14. **^{1}H NMR spectrum of *N*-octylylonicotinamide salicylate (4).**

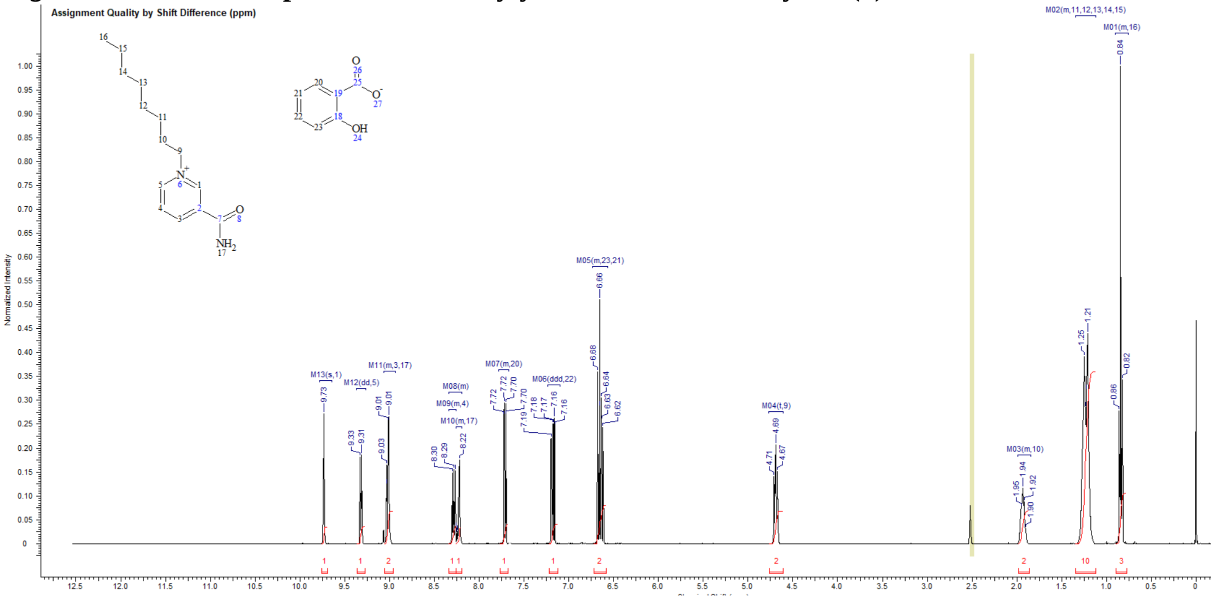

Figure S15. **^{13}C NMR spectrum of *N*-octylonicotinamide salicylate (4).**

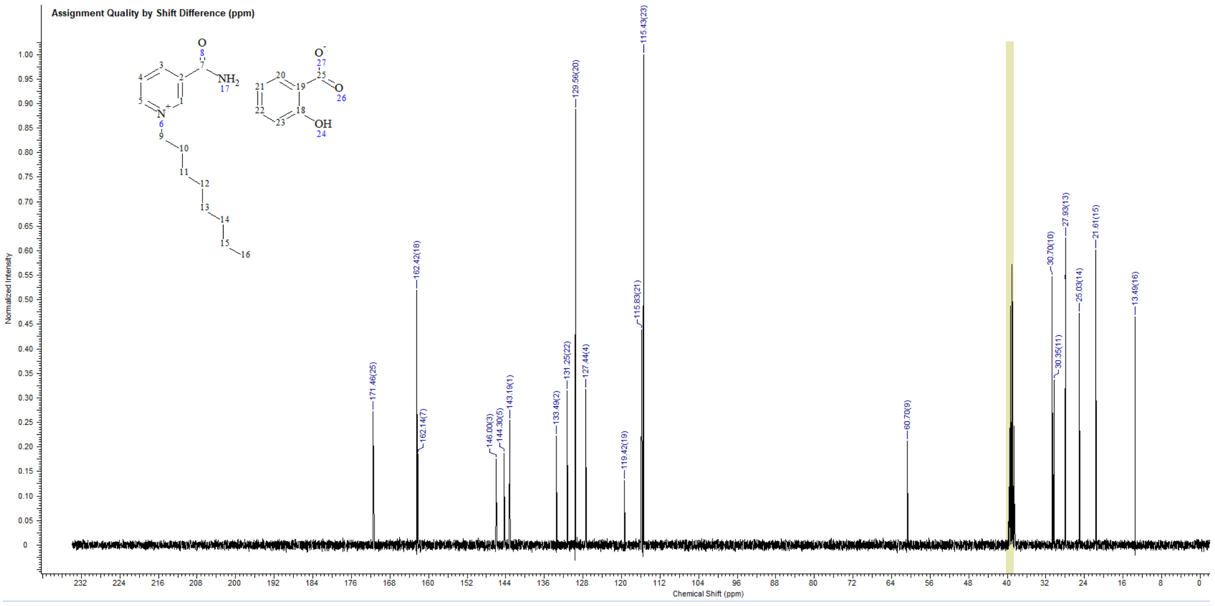

Figure S16. **UV spectrum of *N*-octylonicotinamide salicylate (4).**

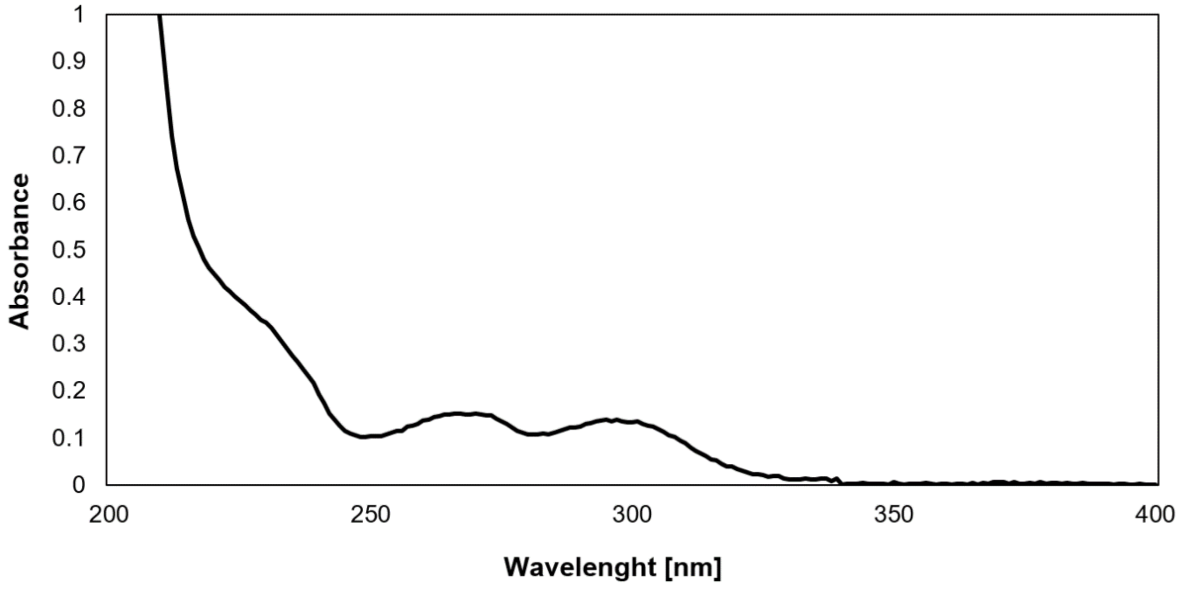

Figure S17. **FT-IR spectrum of *N*-octylonicotinamide salicylate (4).**

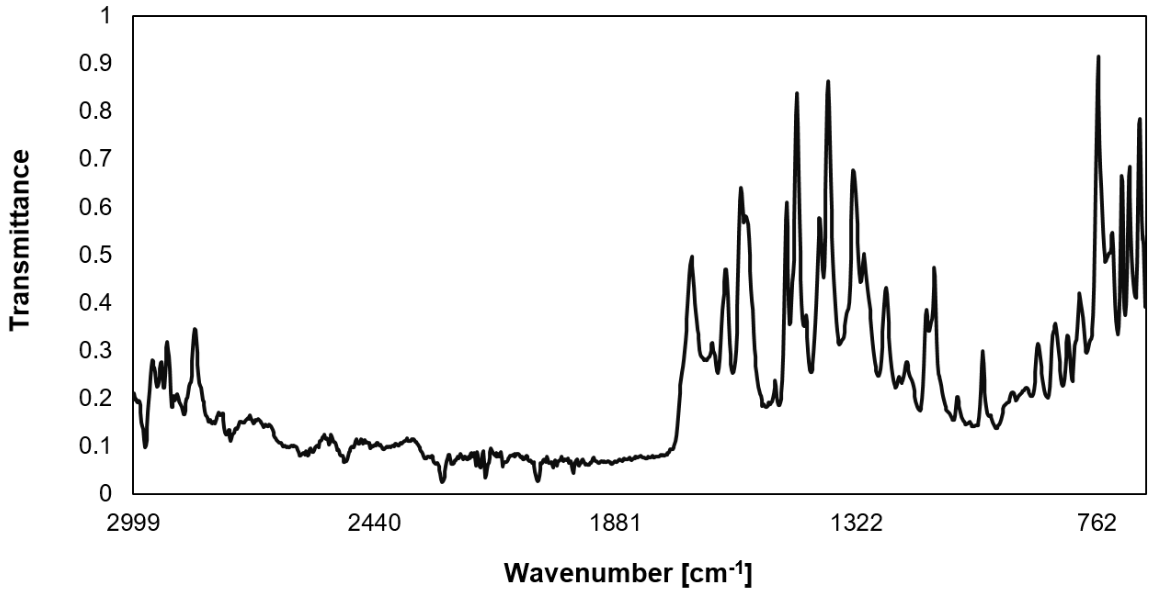

Figure S18. **^{1}H NMR spectrum of *N*-decylonicotinamide salicylate (5).**

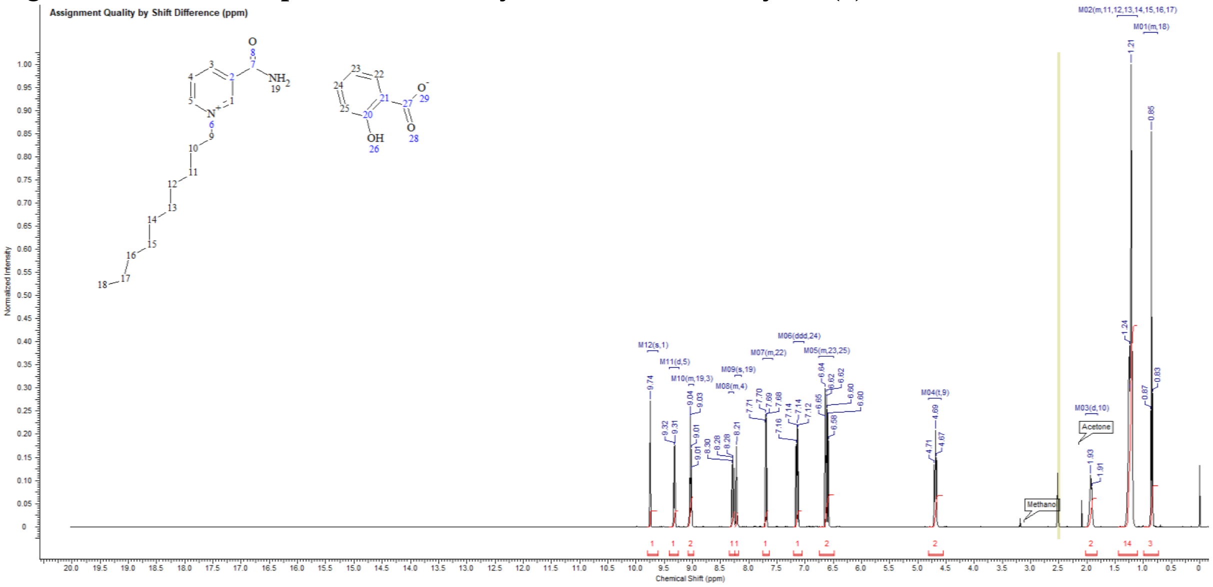

Figure S19. **^{13}C NMR spectrum of *N*-decylonicotinamide salicylate (5).**

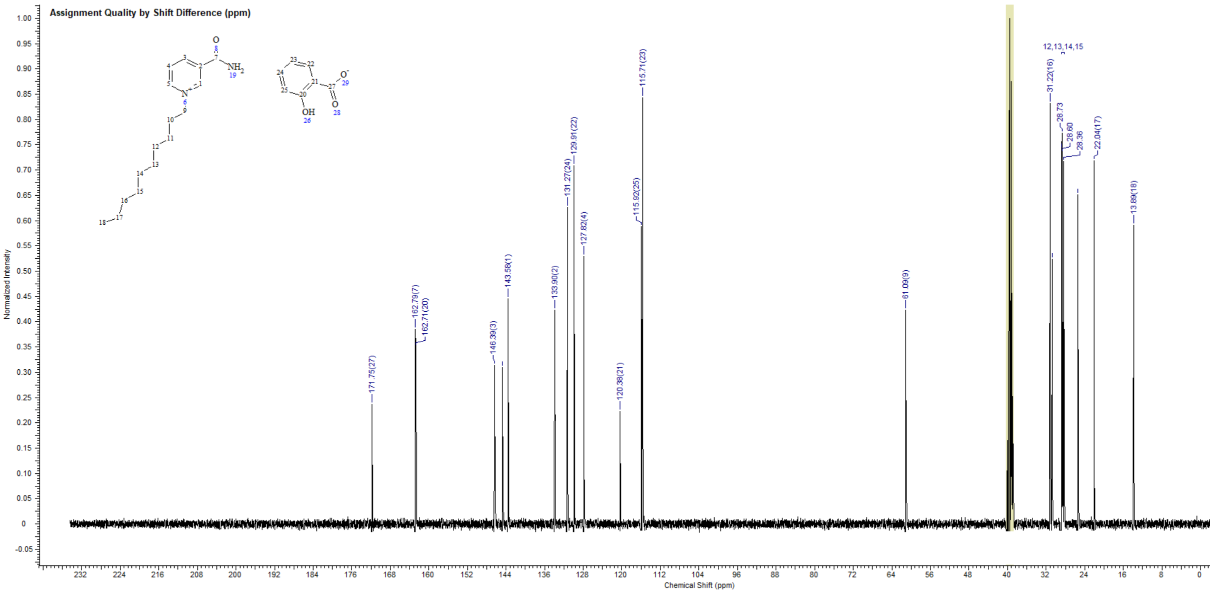

Figure S20. **UV spectrum of *N*-decylonicotinamide salicylate (5).**

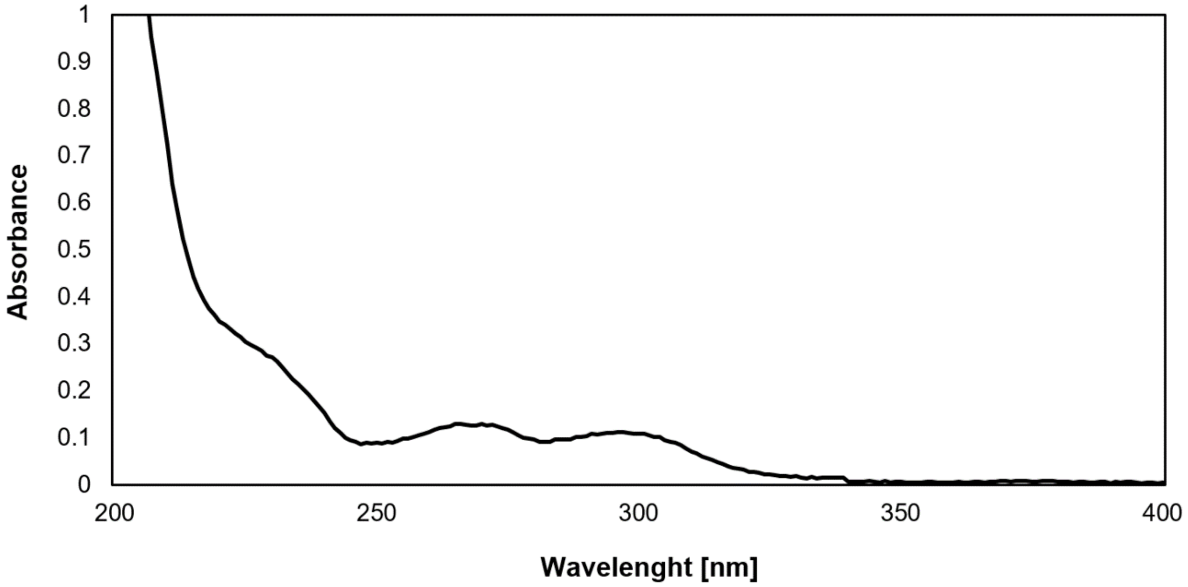

Figure S21. **FT-IR spectrum of *N*-decylonicotinamide salicylate (5).**

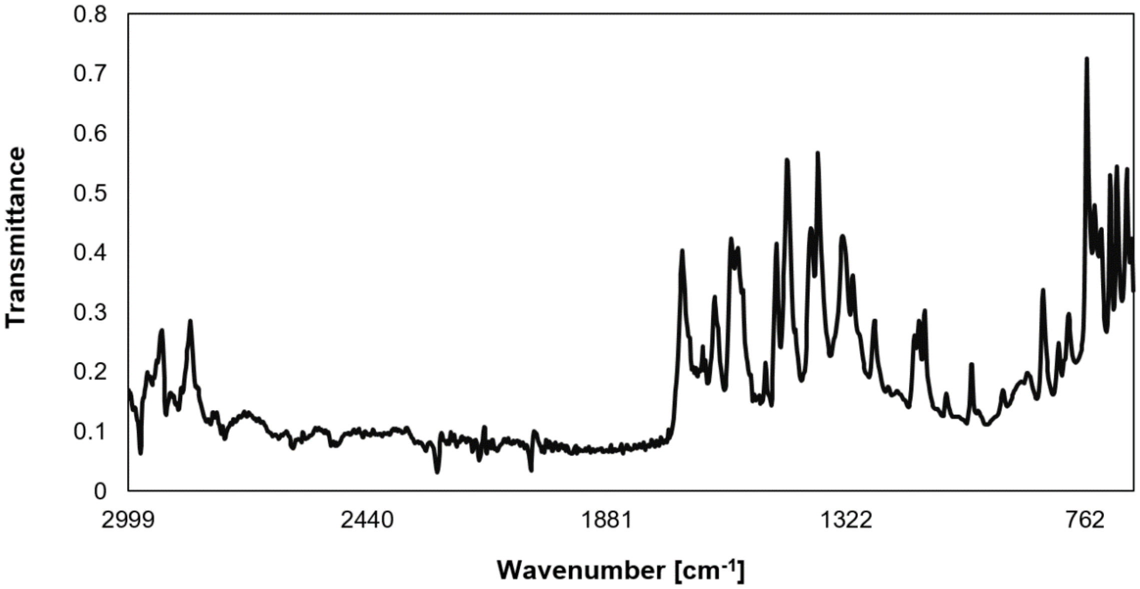

Figure S22. **^{1}H NMR spectrum of *N*-dodecylonicotinamide salicylate (6).**

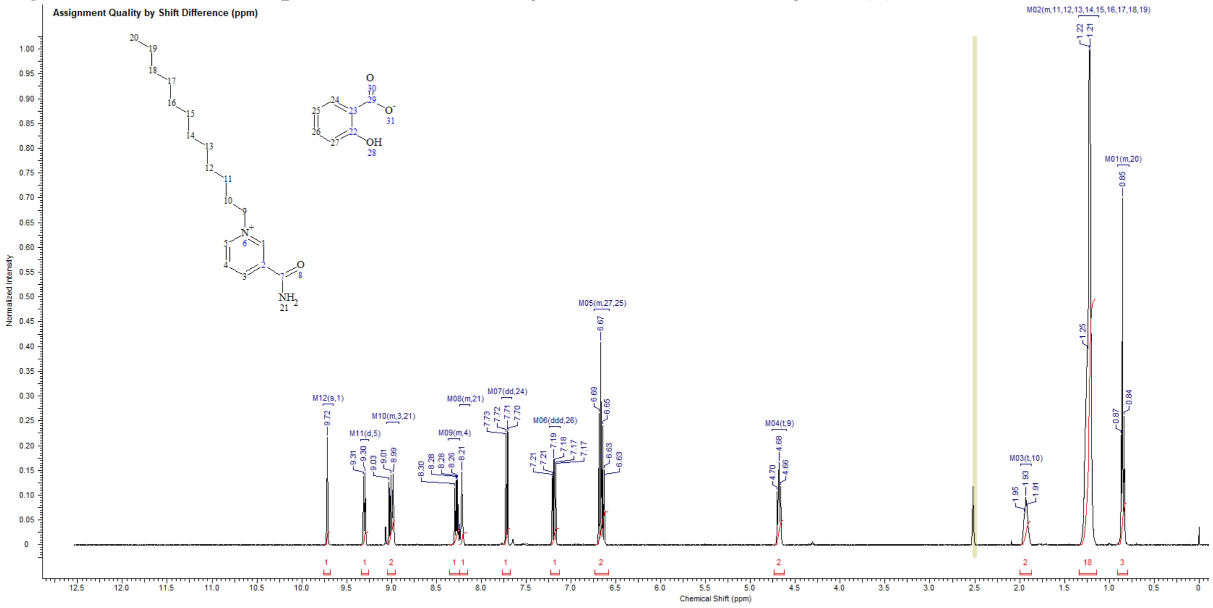

Figure S23. **^{13}C NMR spectrum of *N*-dodecylonicotinamide salicylate (6).**

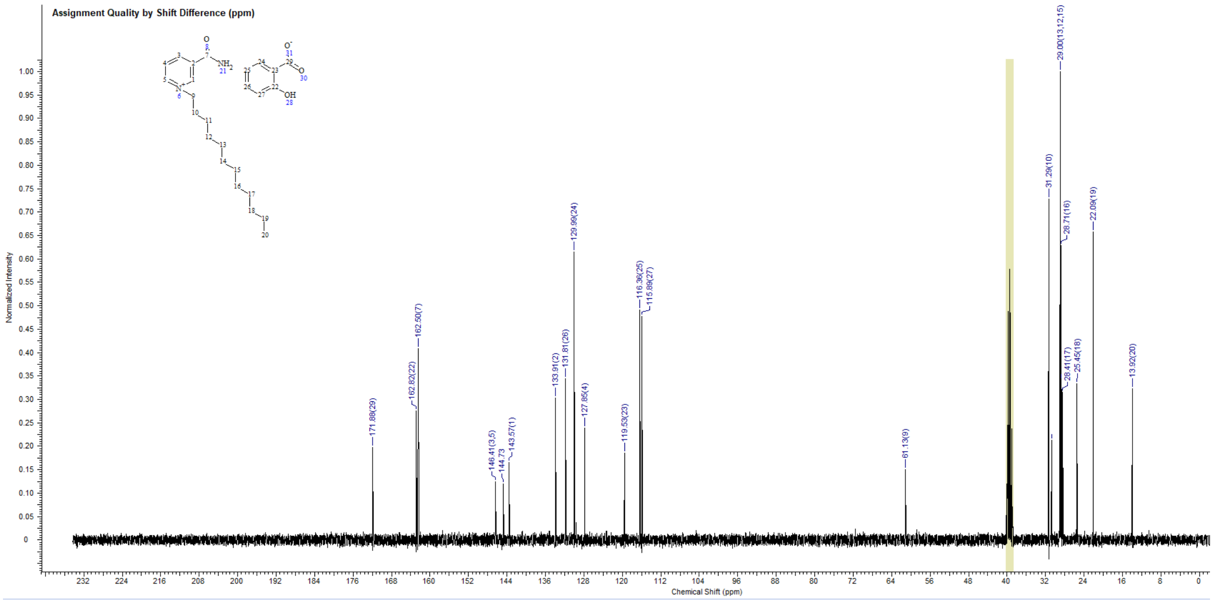

Figure S24. **UV spectrum of *N*-dodecylonicotinamide salicylate (6).**

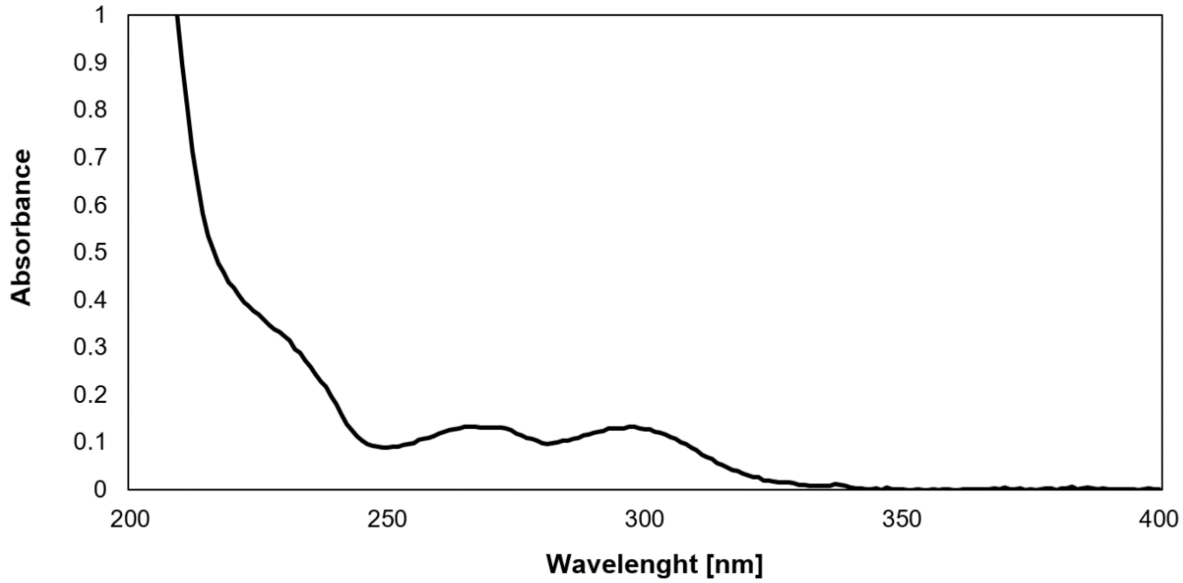

Figure S25. **FT-IR spectrum of *N*-dodecylonicotinamide salicylate (6).**

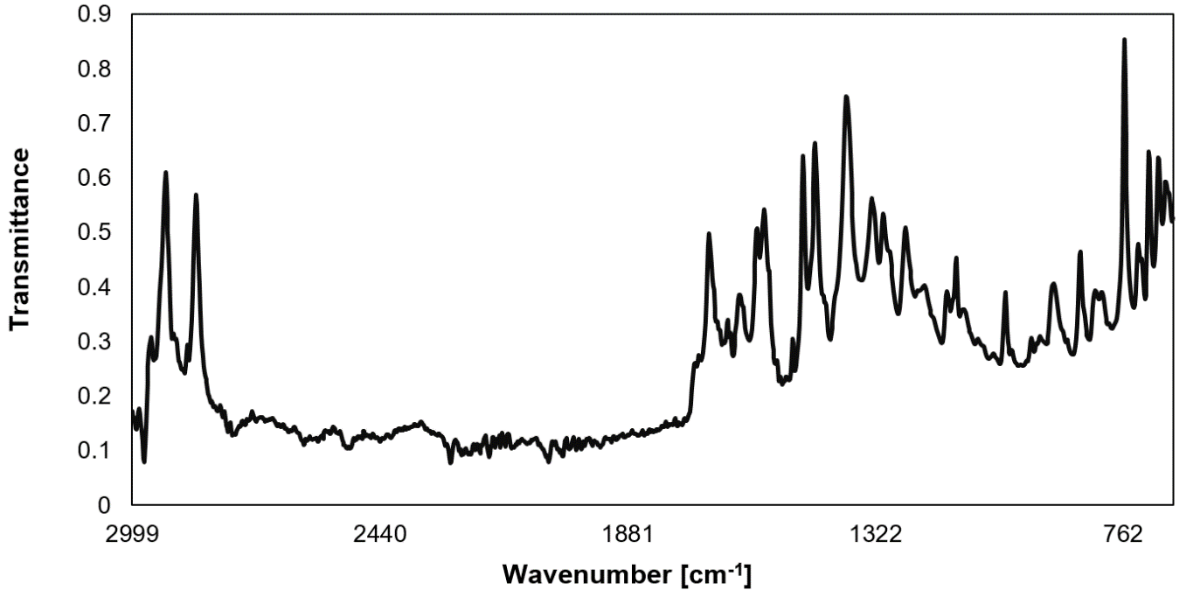

Figure S26. **^{1}H NMR spectrum of *N*-tetradecylonicotinamide salicylate (7).**

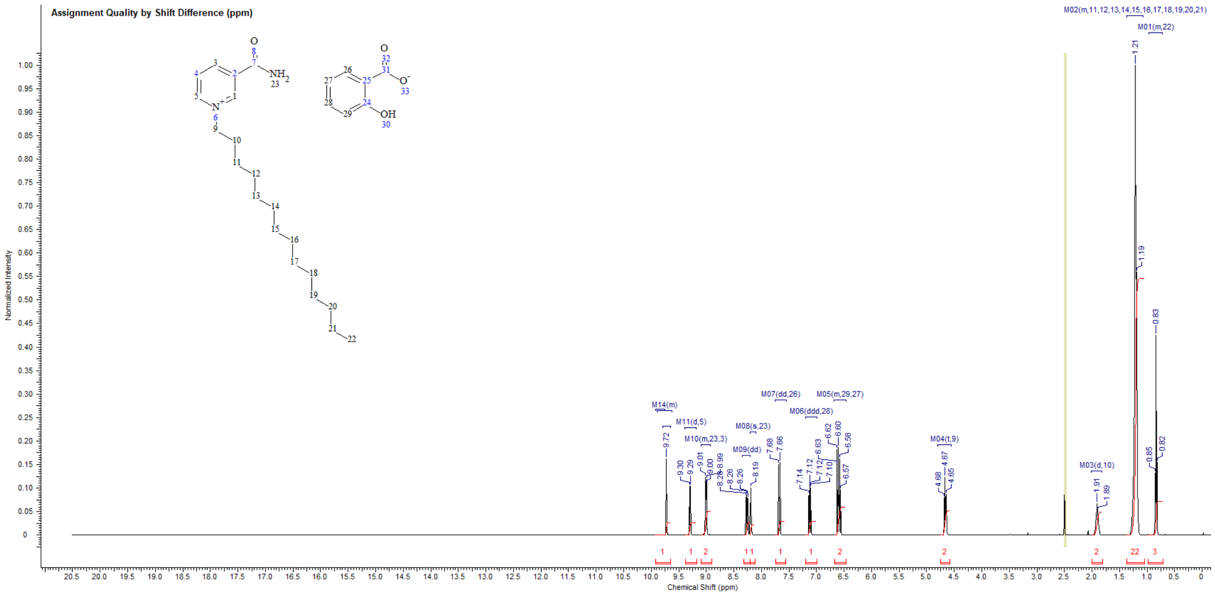

Figure S27. **^{13}C NMR spectrum of *N*-tetradecylonicotinamide salicylate (7).**

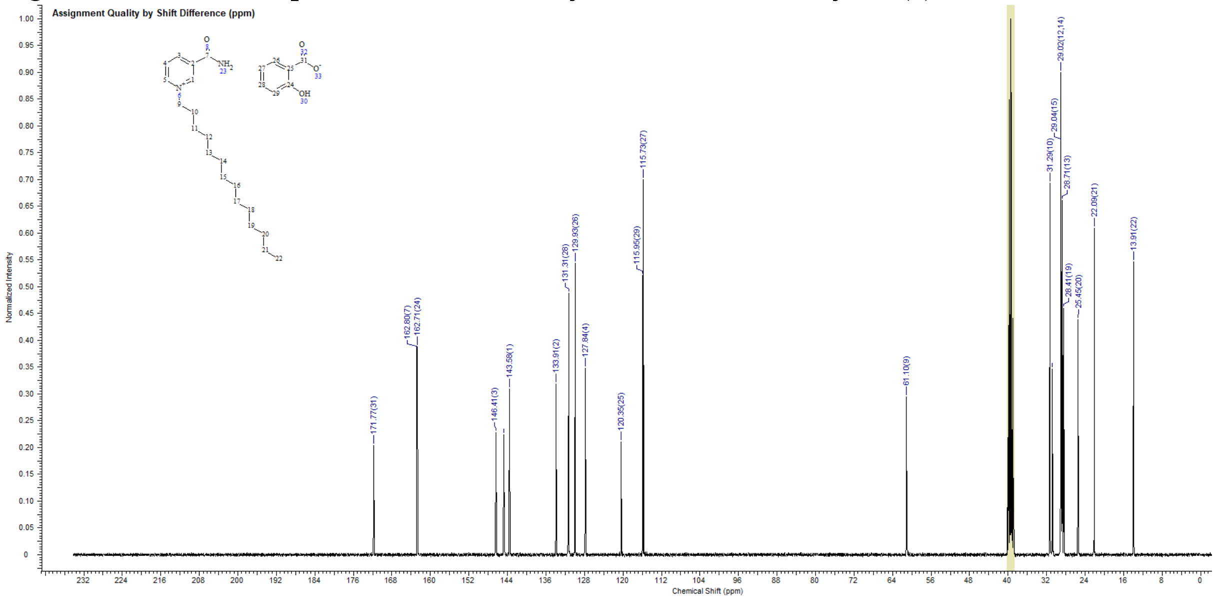

Figure S28. **UV spectrum of *N*-tetradecylonicotinamide salicylate (7).**

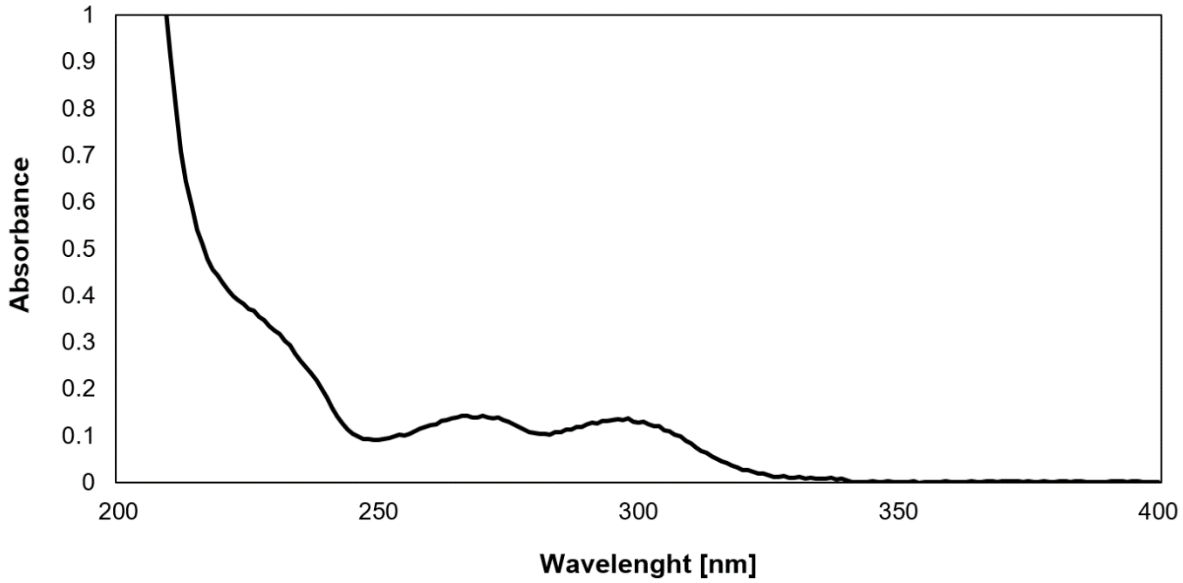

Figure S29. **FT-IR spectrum of *N*-tetradecylonicotinamide salicylate (7).**

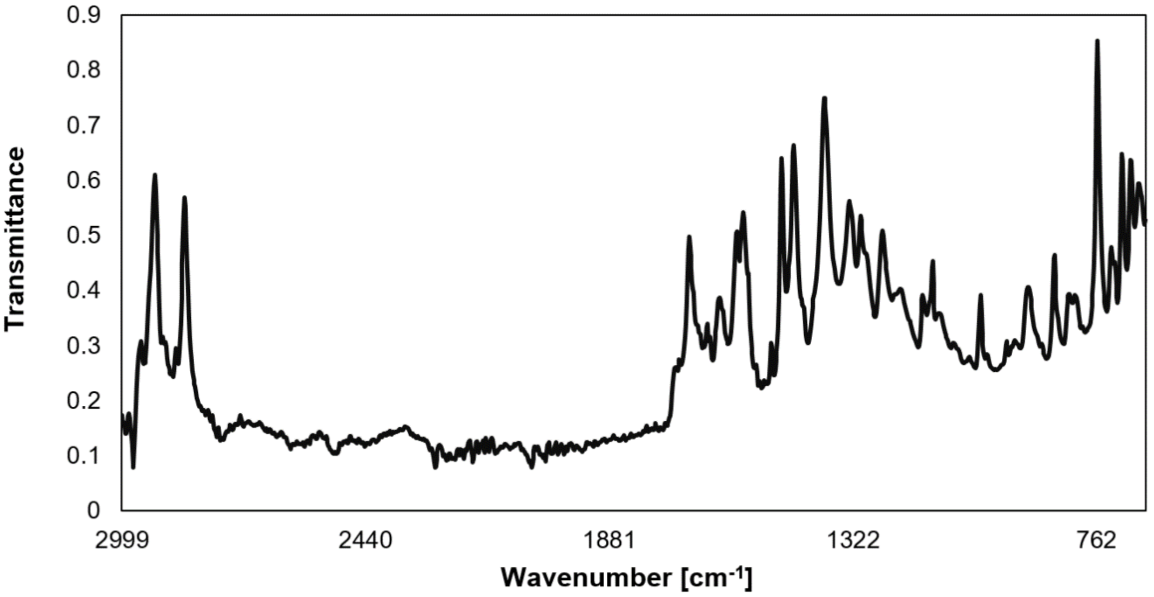

Figure S30. **^{1}H NMR spectrum of cocrystal of nicotinamide and salicylic acid ([NA][SAL]).**

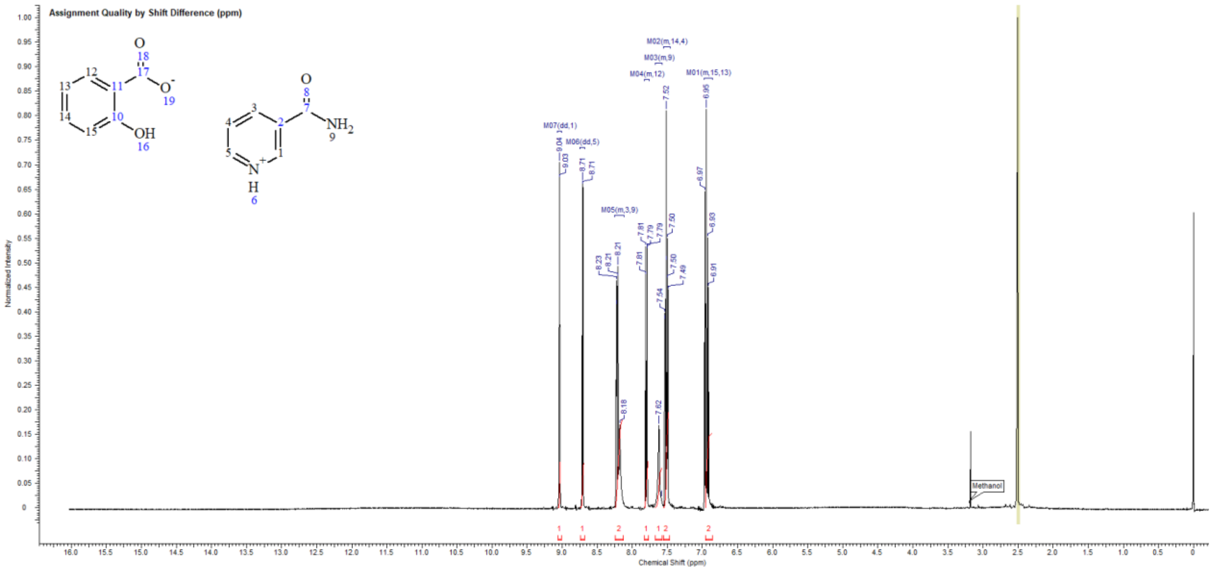

Figure S31. **^{13}C NMR spectrum of cocrystal of nicotinamide and salicylic acid ([NA][SAL]).**

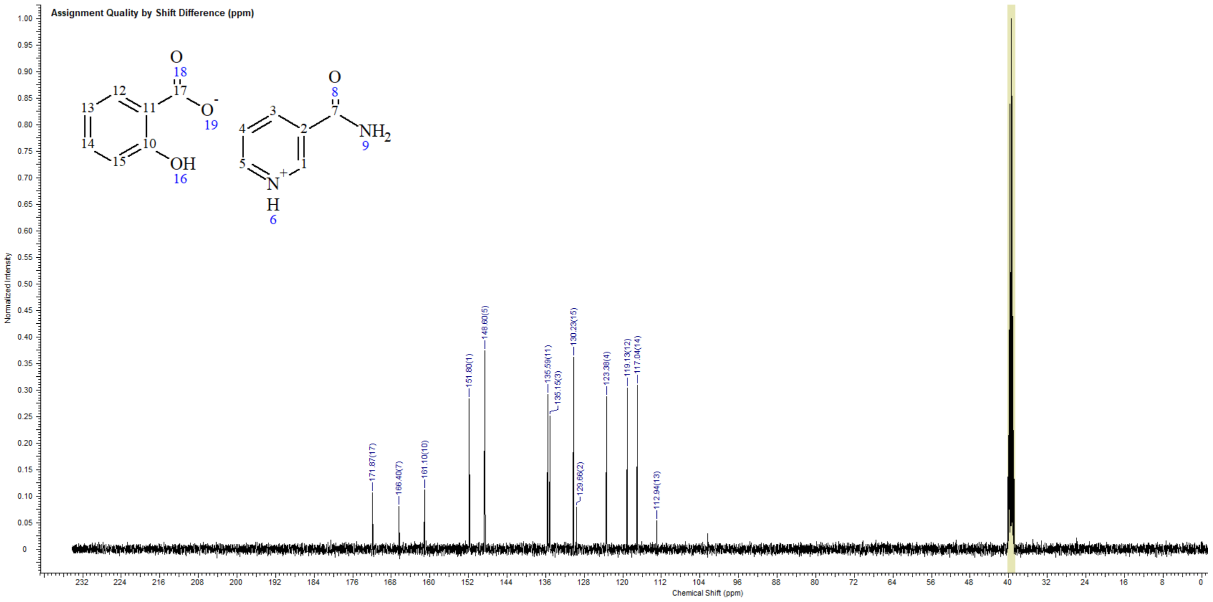

Figure S32. UV spectrum of **cocrystal of nicotinamide and salicylic acid ([NA][SAL]).**

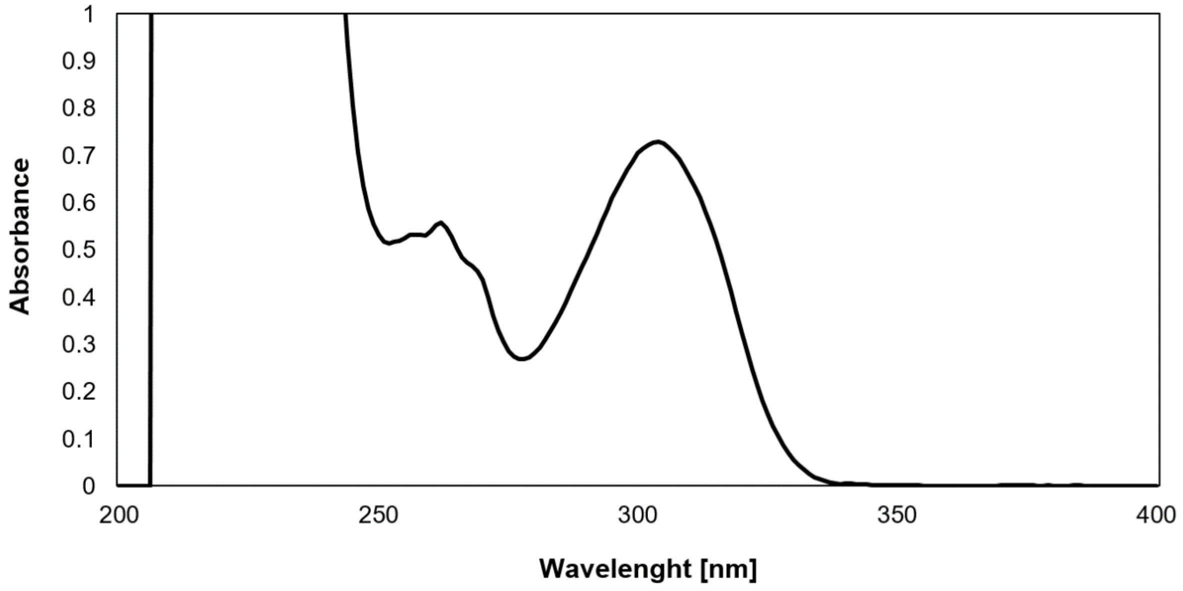

Figure S32. **FT-IR spectrum of cocrystal of nicotinamide and salicylic acid ([NA][SAL]).**

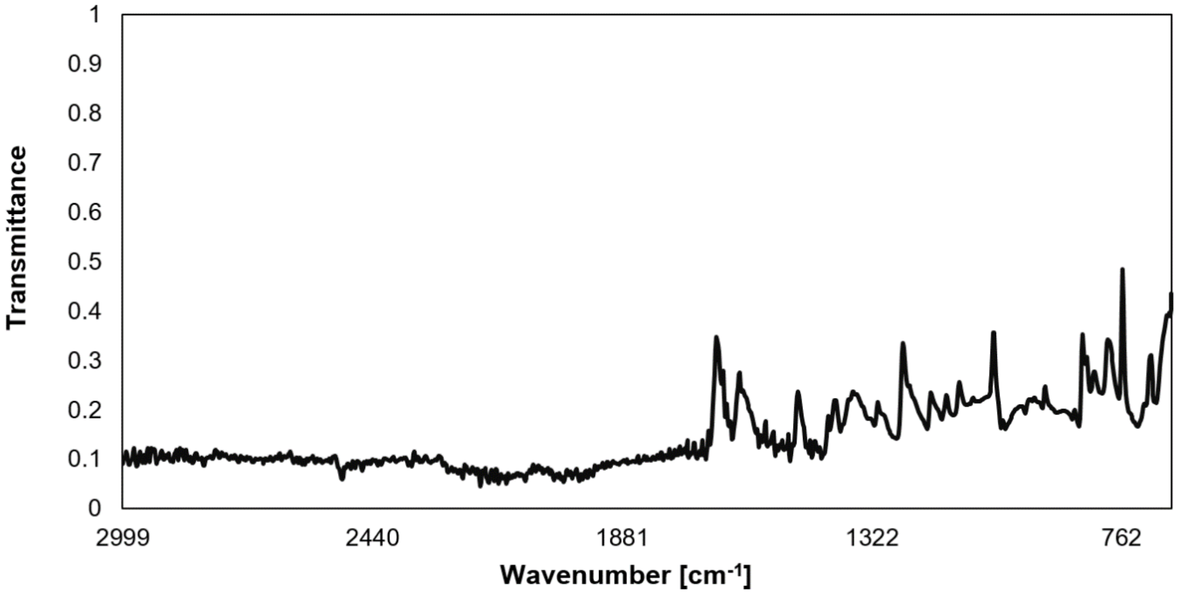

Figure S34. **^{1}H NMR spectrum of salicylic acid (SAL).**

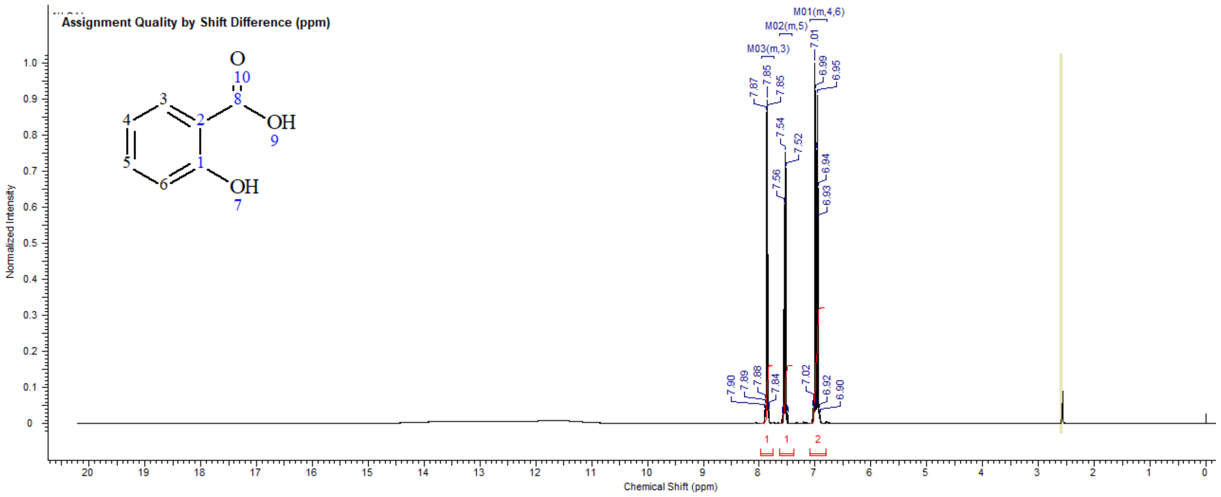

Figure S35. **^{13}C NMR spectrum of salicylic acid (SAL).**

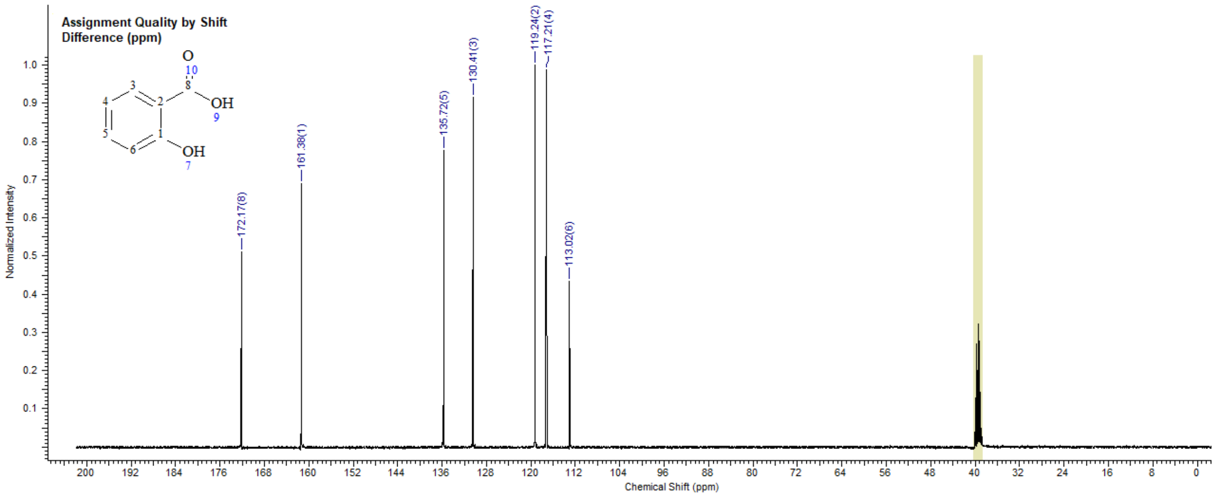

Figure S36. **UV spectrum of salicylic acid (SAL).**

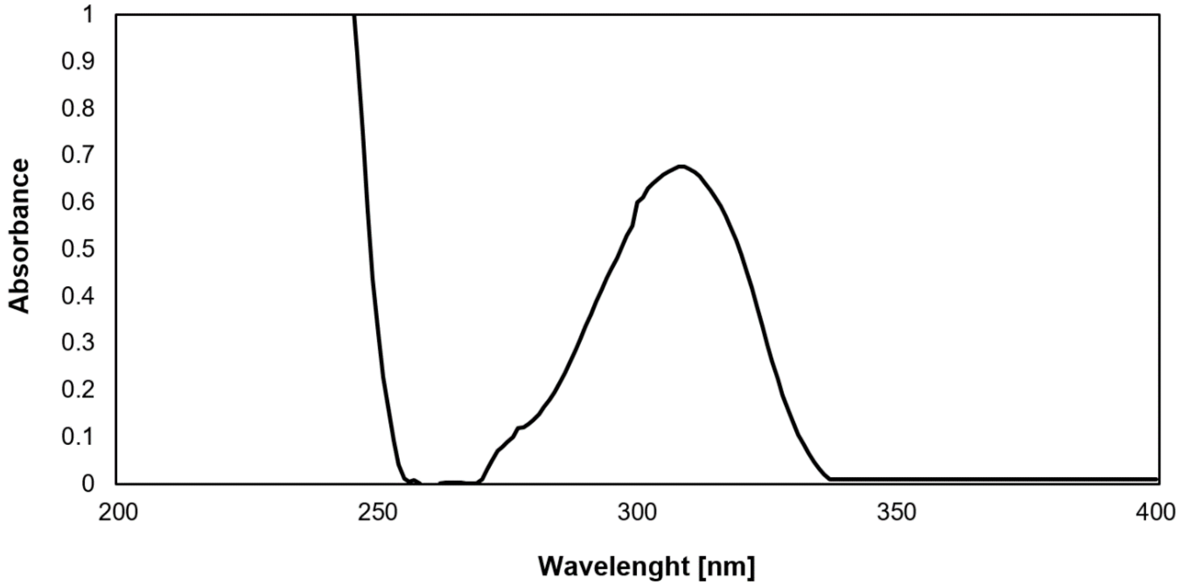

Figure S37. **FT-IR spectrum of salicylic acid (SAL).**

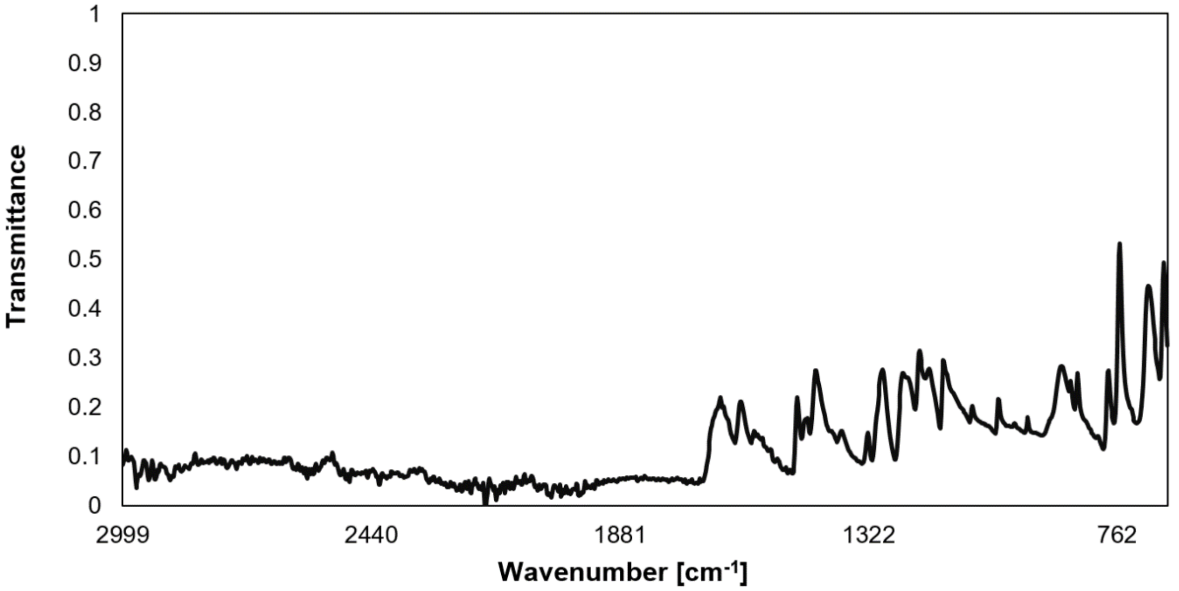

Figure S38. **^{1}H NMR spectrum of nicotinamide (NA).**

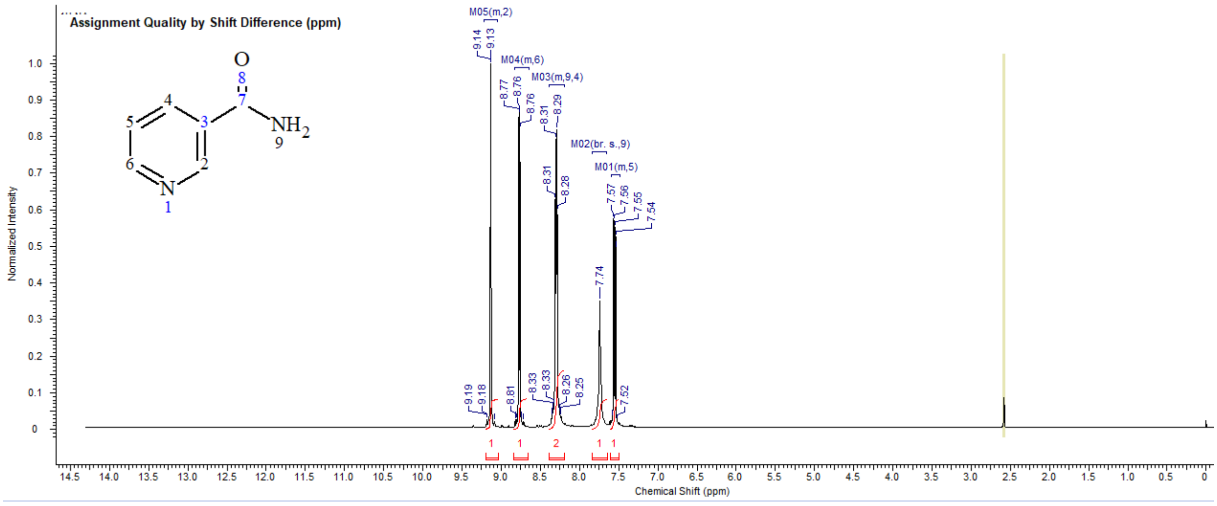

Figure S39. **^{13}C NMR spectrum of nicotinamide (NA).**

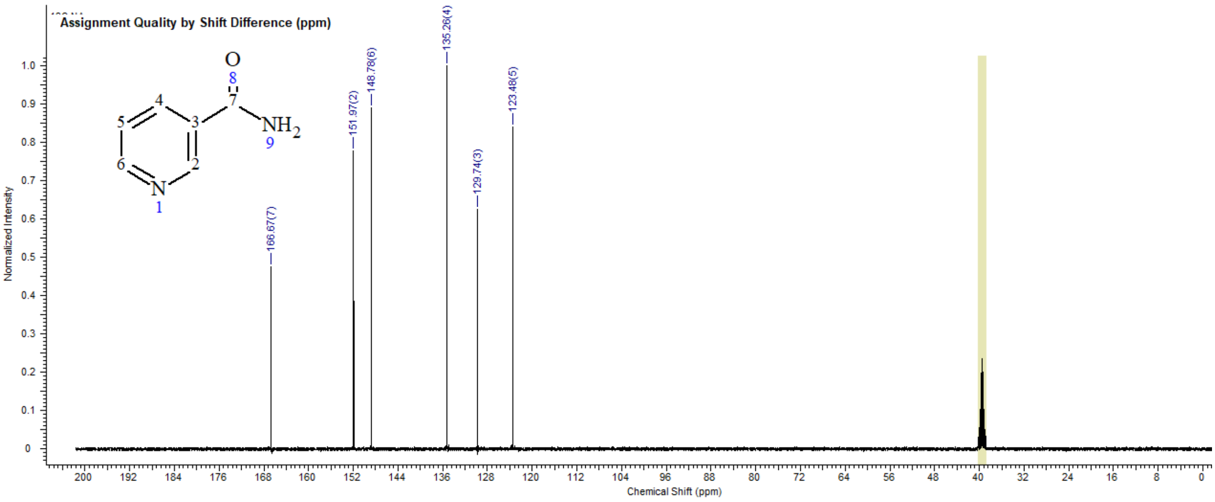

Figure S40. **UV spectrum of nicotinamide (NA).**

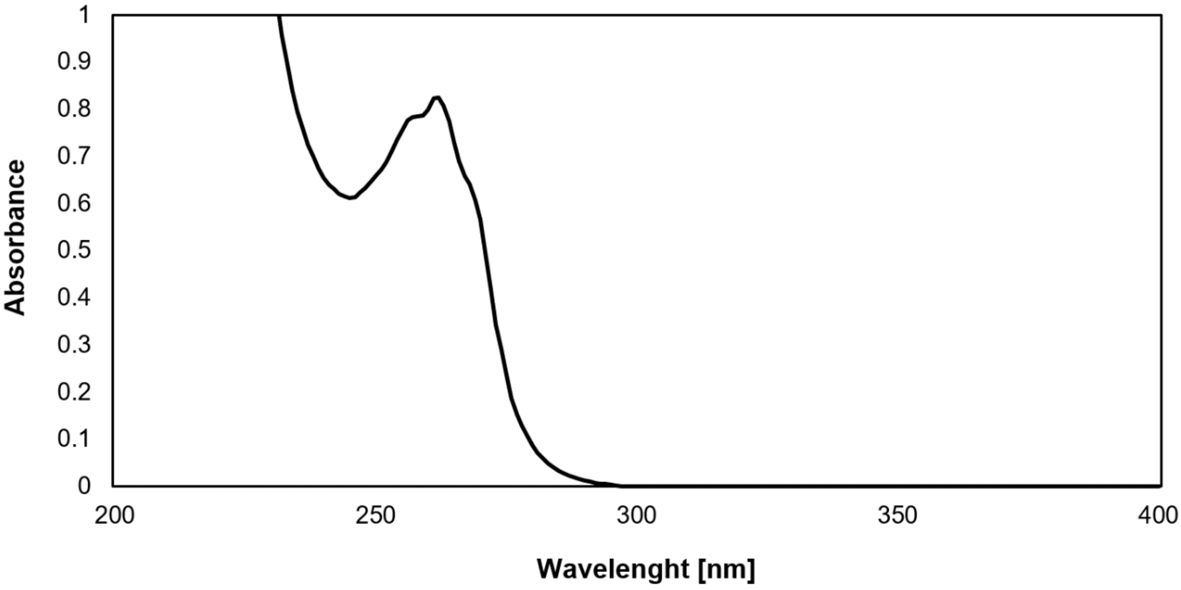

Figure S41. **FT-IR spectrum of nicotinamide (NA).**

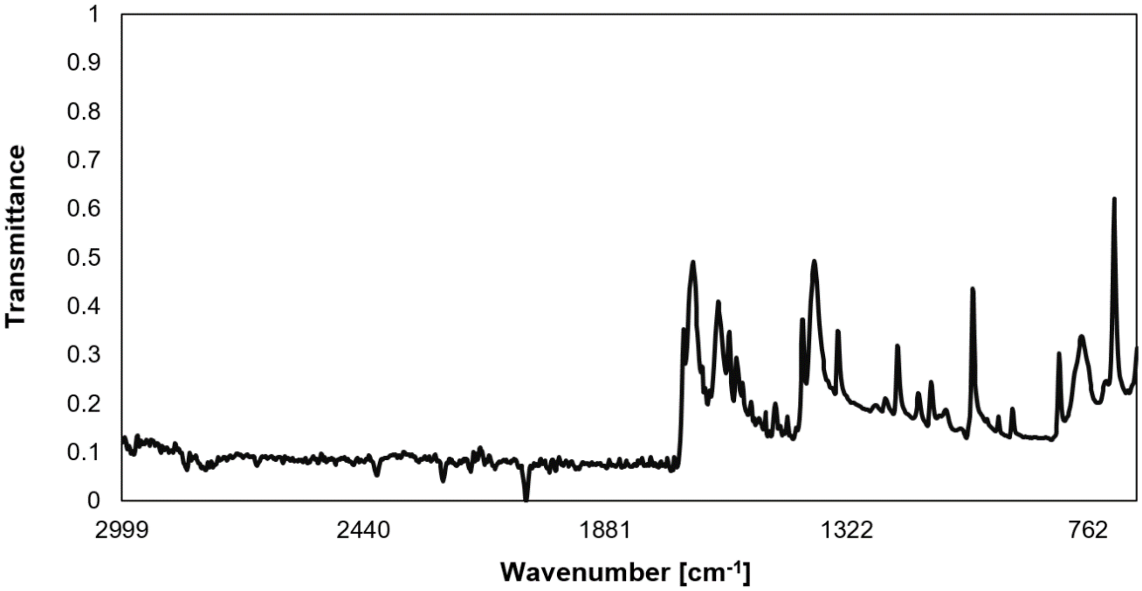

Figure S42. **^{1}H NMR comparison of *N*-decylnicotinamide salicylate (5) and reference substances: cocrystal of nicotinamide and salicylic acid ([NA][SAL]), nicotinamide (NA) and salicylic acid (SAL).**

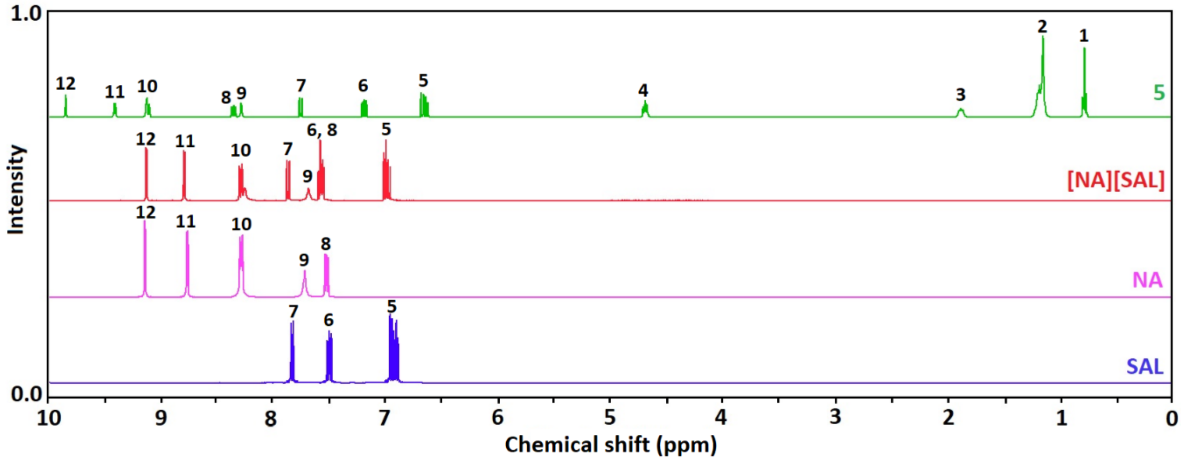

NOTE: In the ^{1}H NMR spectra of *N*-decylnicotinamide salicylate (**5**) and reference substances: nicotinamide salicylate ([**NA**][**SAL**]), nicotinamide (**NA**) and salicylic acid (**SAL**) we can distinguish that the signals on the spectrum of compound **5** shift relative to the substrates, confirming its ionic nature and absence of ionic bonds in the structure of the cocrystal. It is important to note the appearance of signals from the alkyl chain (1–4) in the range from 0.8 to 4.7 ppm, causing significant changes in the chemical shift values of the compound. Signals from **NA** (8–12) occur between 7.5 and 9.7 ppm, with notable shifts in *N*-decylnicotinamide salicylate toward higher values of chemical shifts and differences in values of up to 0.7 ppm, comparing with pure **NA**. The hydrogen moiety of **SAL** (5–7) is apparent in the range 6.6–7.7 ppm and move towards lower values in **5**, thus deviating from those observed for the starting acid. Interestingly, no differences can be distinguished in the appearance of peaks on the ^{1}H NMR spectrum of [**NA**][**SAL**] compared to its components that interact only through hydrogen bonds. The absence of any proton transfer, results in a summed **NA** and **SAL** ^{1}H NMR spectrum.

Table S1. **Water and bromide ions content in analyzed compounds.**

Compound	Water content [%]	Bromide ions content [ppm]
**1**	2.203	6842
**2**	2.582	8491
**3**	0.539	4570
**4**	0.808	3263
**5**	0.812	2905
**6**	0.663	7536
**7**	1.694	8249
**[NA][SAL]**	1.124	-
**NA**	0.779	-
**SAL**	0.259	-

Table S2. **Green Chemistry Metrics of *N*-alkylnicotinamide salicylates (1-7).**

Compound	Atom economy [%]	Percentage yield [%]	Reaction mass efficiency [%]	Environmental factor
**1**	70.78	90.25	63.88	0.43
**2**	72.67	85.44	62.09	0.42
**3**	74.32	85.50	63.54	0.38
**4**	75.79	88.27	66.90	0.35
**5**	77.09	78.40	60.44	0.37
**6**	78.27	82.32	64.43	0.33
**7**	79.33	87.22	69.19	0.29

Table S3. **Molecular mass of analyzed compounds.**

Compound	Molecular weight [Da]
**1**	288.30
**2**	316.36
**3**	344.41
**4**	372.47
**5**	400.52
**6**	428.57
**7**	456.63
**[NA][SAL]**	260.25
**NA**	122.13
**SAL**	138.12

Table S4. **Octanol-water partition coefficient (log K_{ow}) determined for analyzed compounds.**

Compound	log K_{ow}
**1**	-1.15
**2**	-0.70
**3**	-0.18
**4**	0.07
**5**	0.59
**6**	0.60
**7**	1.34
**[NA][SAL]**	-0.35/0.93[a]
**NA**	-0.66
**SAL**	1.82

[a] values given separately for part of nicotinamide **(NA)** and subsequent for part of salicylic acid **(SAL)**.

Table S5. **Skin permeability coefficients for *N*-alkylnicotinamide salicylates (1–7) and reference substances.**

Compound	Kp (cm/h)		
	**Frasch ($\cdot 10^7$)**	**Potts and Guy ($\cdot 10^6$)**	**Modified Robinson ($\cdot 10^5$)**
**1**	3.58	4.84	1.77
**2**	5.09	6.81	2.03
**3**	8.76	10.7	2.60
**4**	8.73	10.9	2.65
**5**	16.9	17.2	3.64
**6**	9.99	11.8	2.93
**7**	39.5	26.7	5.43
**[NA][SAL] – NA**<br>**[NA][SAL] – SAL**	32.5<br>618	26.5<br>215	4.74<br>23.8
**NA**	268	111	21.2
**SAL**	65 300	5 120	474

[a] values given separately for part of nicotinamide (**NA**) – above and for part of salicylic acid (**SAL**) – below.

Table S6. **Solubility in PBS measured for *N*-alkylnicotinamide salicylates (1-7) and reference substances: cocrystal of nicotinamide and salicylic acid ([NA][SAL]), nicotinamide (NA) and salicylic acid (SAL).**

Compound	PBS [ml]	Class
**1**	<1	Very Soluble
**2**	<1	Very Soluble
**3**	<1	Very Soluble
**4**	10-30	Soluble
**5**	100-1000	Slightly Soluble
**6**	1000-10 000	Very Slightly Soluble
**7**	1000-10 000	Very Slightly Soluble
**[NA][SAL]**	30-100	Sparingly Soluble
**[NA]**	<1	Very Soluble
**[SAL]**	100-1000	Slightly Soluble

Table S7. **Contact angle (°) of *N*-alkylnicotinamide salicylates (1-7) and reference substances: cocrystal of nicotinamide and salicylic acid ([NA][SAL]), nicotinamide (NA) and salicylic acid (SAL) in selected concentrations.**

**Compound**	**Concentration [%]**				
	**0.01**	**0.03**	**0.25**	**0.50**	**1.00**
**1**	-	-	112.1	110.3	109.7
**2**	-	-	102.7	104.3	103.9
**3**	-	-	107.0	103.4	98.51
**4**	-	-	94.1	79.0	61.2
**5**	-	-	50.7	-[a]	-[a]
**6**	-	99.3[b]	-	-	-
**7**	106.1[b]	-	-	-	-
**[NA][SAL]**	-	-	110.5	108.3	99.9
**NA**	-	-	112.7	110.7	110.6
**SAL**	-	-	104.9	-[a]	-[a]

[a] due to the limited solubility of the substance in water, it was not possible to perform the test; [b] the concentrations for 6 and 7 were selected on the basis of their effective dose estimated after analysis of MIC/MBC/MFC tests

Figure S43. **Solutions prepared to test contact angle (°) of the analyzed substances: *N*-alkylnicotinamide salicylates (1-7), cocrystal of nicotinamide and salicylic acid ([NA][SAL]), nicotinamide (NA) and salicylic acid (SAL) in selected concentrations.**

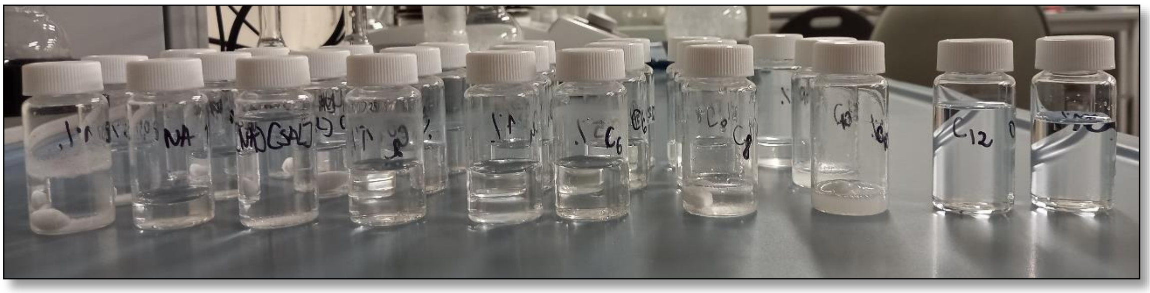

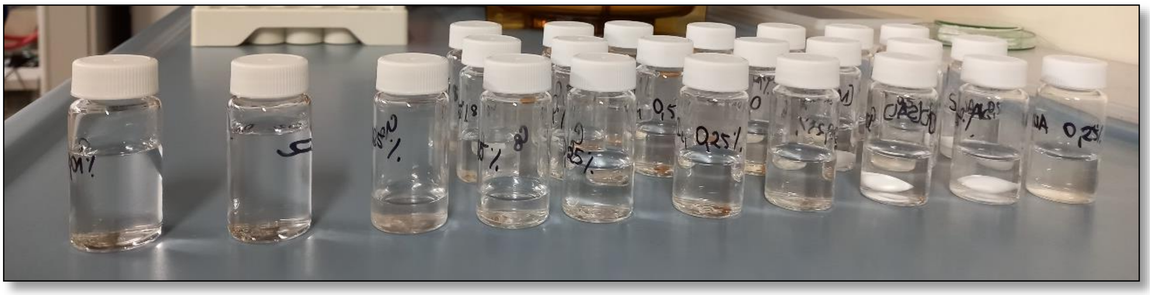

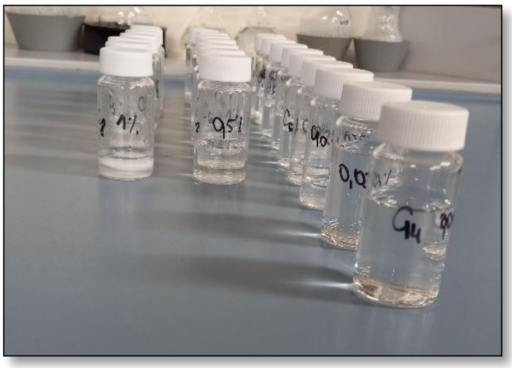

Table S8. **Binding energies and inhibition constant of ligands docked to cyclooxygenase-2 (PDB ID: 1CX2) enzyme.**

Ligand		RMSD	Binding energy [Kcal/mol]	Inhibition constant (Ki)	No of H-bonds (ligand-protein) PLIP
**1**	**[C_2NA]**	32.65	-4.44	559 µM	2
**2**	**[C_4NA]**	33.74	-5.10	182 µM	2
**3**	**[C_6NA]**	34.15	-6.05	36.5 µM	2
**4**	**[C_8NA]**	34.32	-6.72	11.9 µM	4
**5**	**[C_{10}NA]**	35.16	-6.95	8.10 µM	2
**6**	**[C_{12}NA]**	35.37	-7.46	3.39 µM	4
**7**	**[C_{14}NA]**	36.32	-6.37	21.4 µM	2
**INDO**	**Indometacine**	34.17	-8.25	892 nM	2
**IBU**	**Ibuprofen**	32.76	-6.27	25.5 µM	1
**ASA**	**Aspirine**	34.91	-5.18	160 µM	2
**NA**	**Nicotinamide**	32.34	-4.59	428 µM	4
**SAL**	**Salicylic acid**	32.3	-4.34	658 µM	4

Figure S44. **Docked pose of S58 *(cocrystalyzed ligand*, binding energy -11.55 kcal/mol) with the active site region of cyclooxygenase-2 (PDB ID: 1CX2) enzyme. Hydrogen bonds were indicated by blue lines, hydrophobic interactions by grey dotted lines. The ligand was highlighted in orange.**

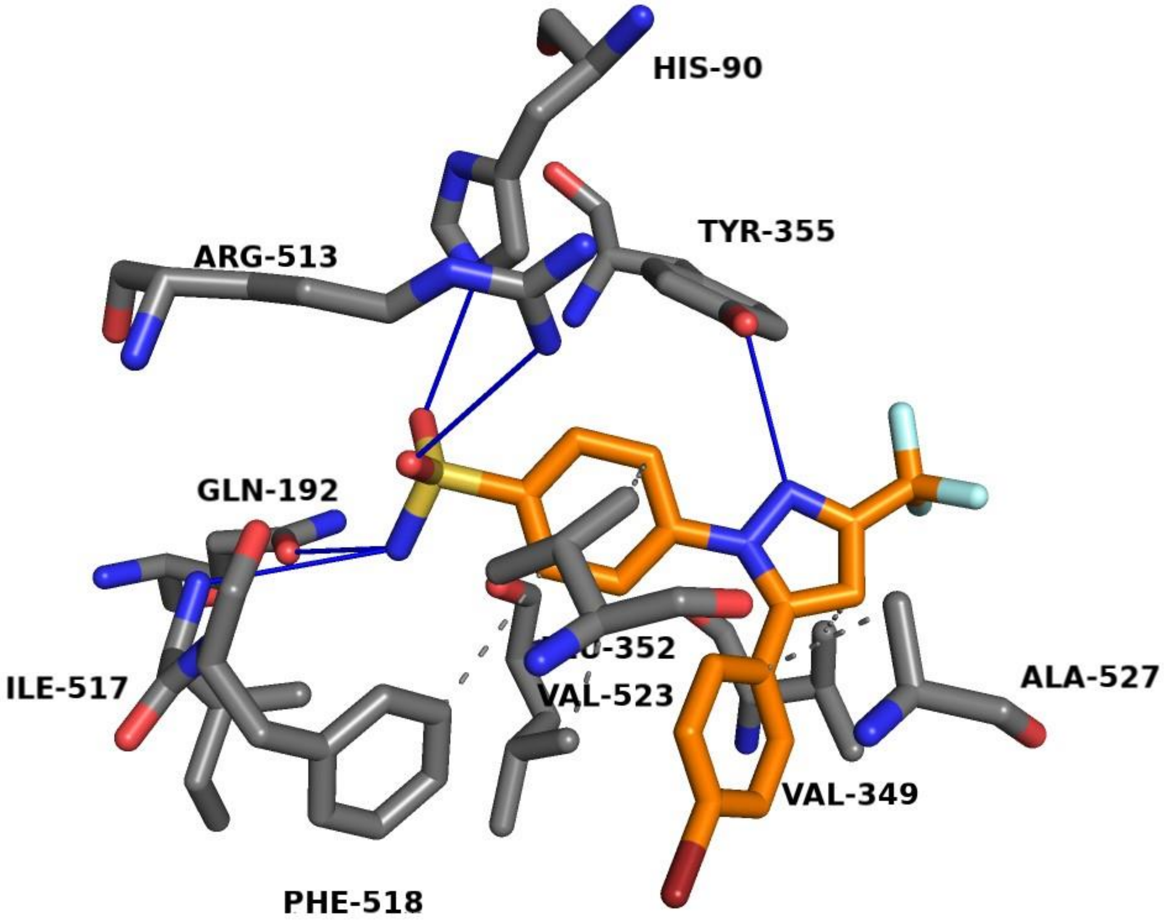

***Figure S45*.** **Docked pose of indometacine (binding energy -8.25 kcal/mol) with the active site region of cyclooxygenase-2 (PDB ID: 1CX2) enzyme. Hydrogen bonds were indicated by blue lines, hydrophobic interactions by grey dotted lines. The ligand was highlighted in orange.**

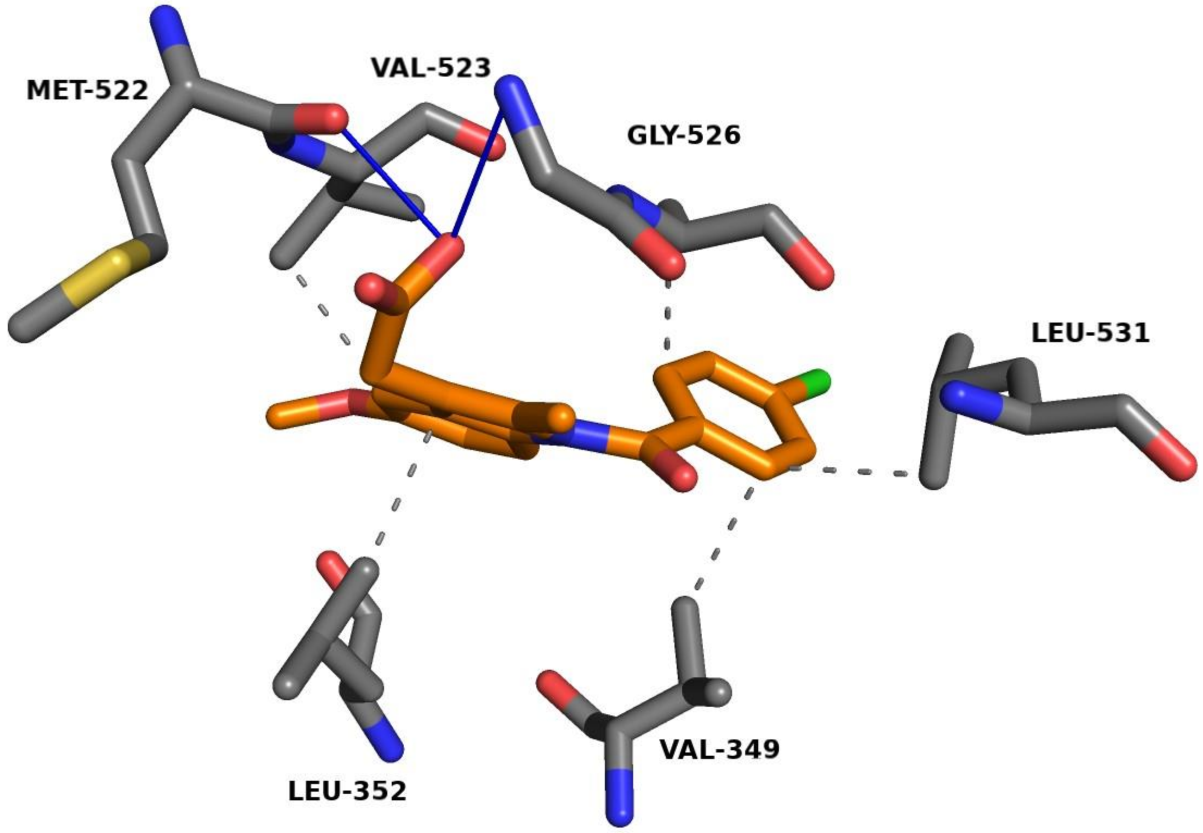

Figure S46. **Docked pose of ibuprofen (binding energy -6.27 kcal/mol) with the active site region of cyclooxygenase-2 (PDB ID: 1CX2) enzyme. Hydrogen bonds were indicated by blue lines, hydrophobic interactions by grey dotted lines. The ligand was highlighted in orange.**

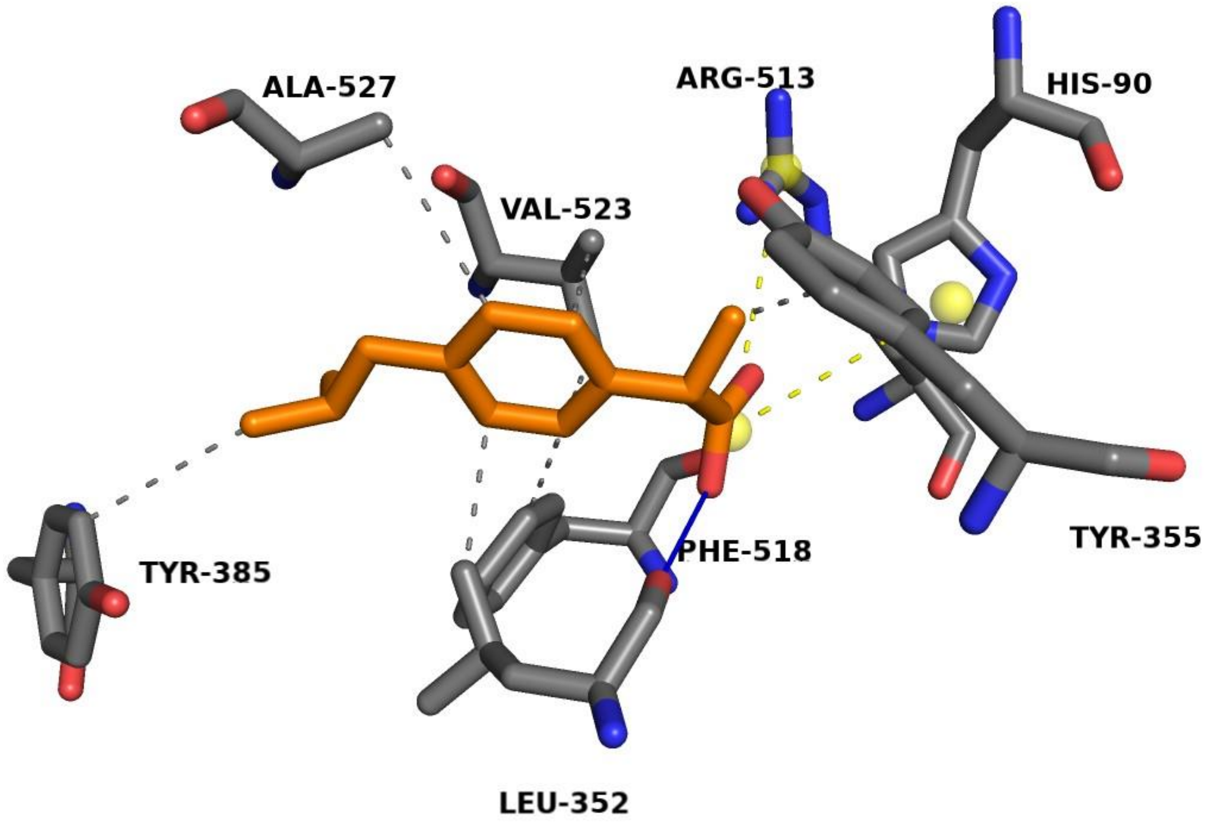

Figure S47. **Docked pose of aspirine (binding energy -5.18 kcal/mol) with the active site region of cyclooxygenase-2 (PDB ID: 1CX2) enzyme. Hydrogen bonds were indicated by blue lines, hydrophobic interactions by grey dotted lines. The ligand was highlighted in orange.**

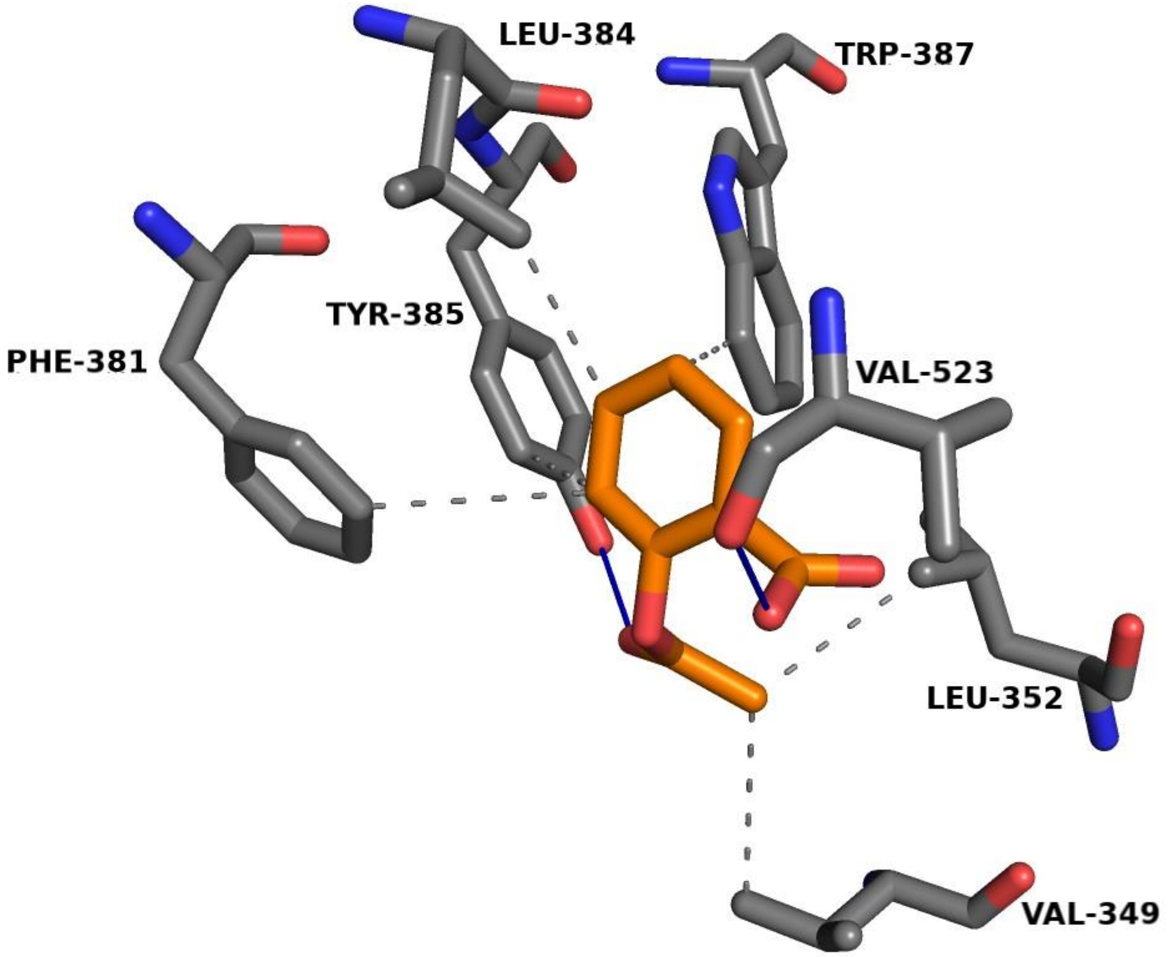

Figure S48. **Docked pose of salicylic acid (SAL, binding energy -4.34 kcal/mol) with the active site region of cyclooxygenase-2 (PDB ID: 1CX2) enzyme. Hydrogen bonds were indicated by blue lines, hydrophobic interactions by grey dotted lines. The ligand was highlighted in orange.**

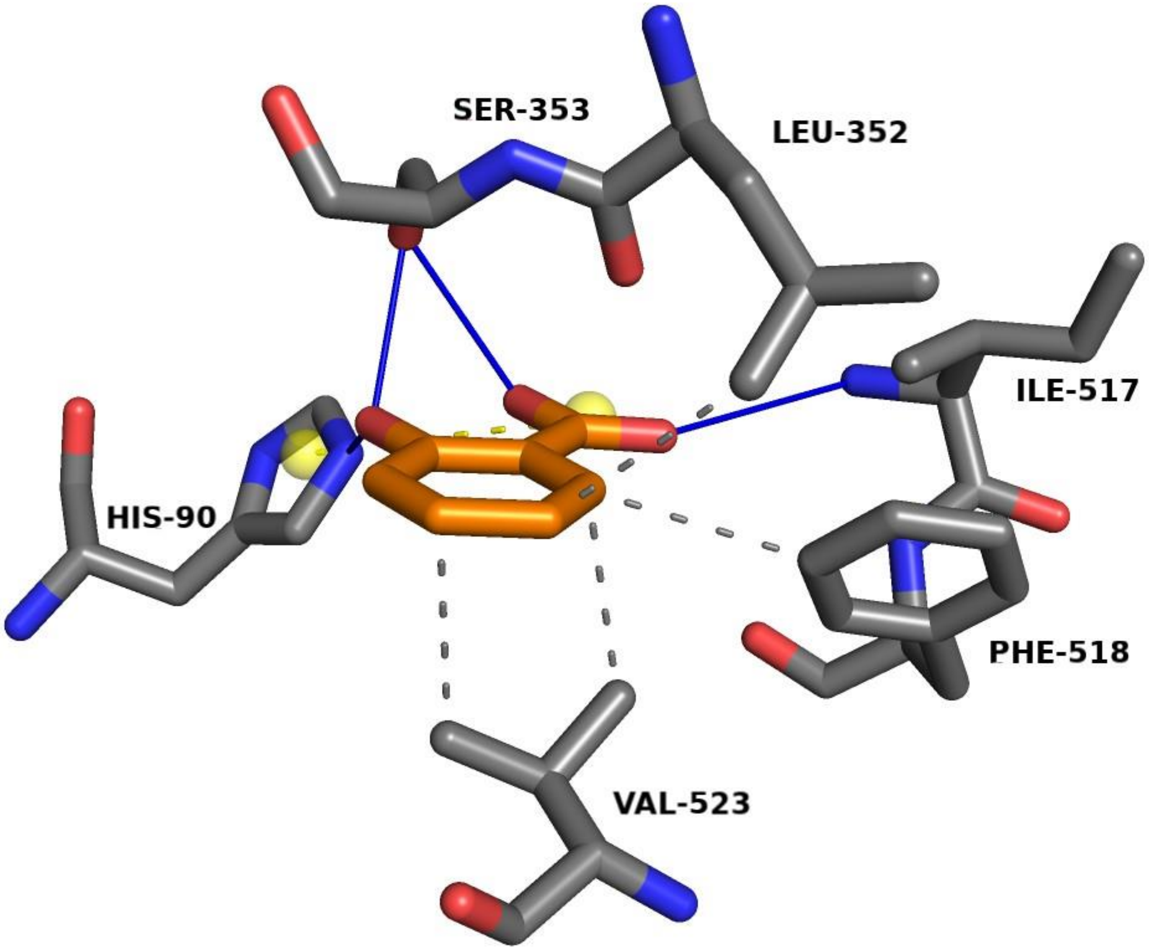

Figure S49. **Docked pose of nicotinamide (NA, binding energy -4.59 kcal/mol) with the active site region of cyclooxygenase-2 (PDB ID: 1CX2) enzyme. Hydrogen bonds were indicated by blue lines, hydrophobic interactions by grey dotted lines. The ligand was highlighted in orange.**

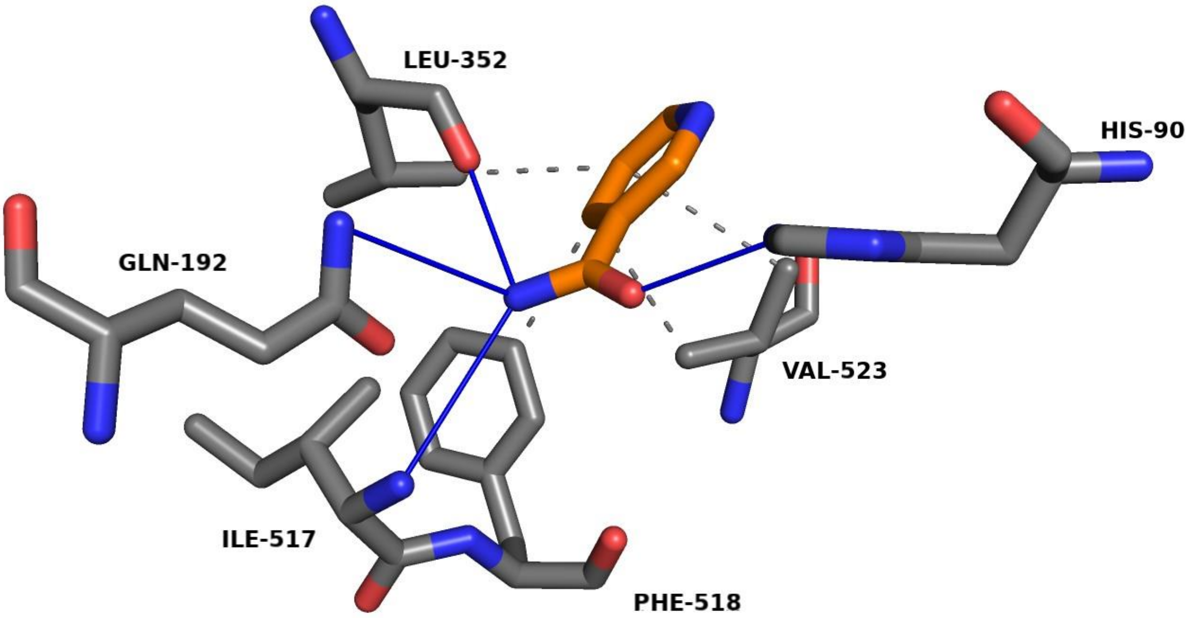

Figure S50. **Docked pose of *N*-ethylnicotinamide cation (1, binding energy -4.44 kcal/mol) with the active site region of cyclooxygenase-2 (PDB ID: 1CX2) enzyme. Hydrogen bonds were indicated by blue lines, hydrophobic interactions by grey dotted lines. The ligand was highlighted in orange.**

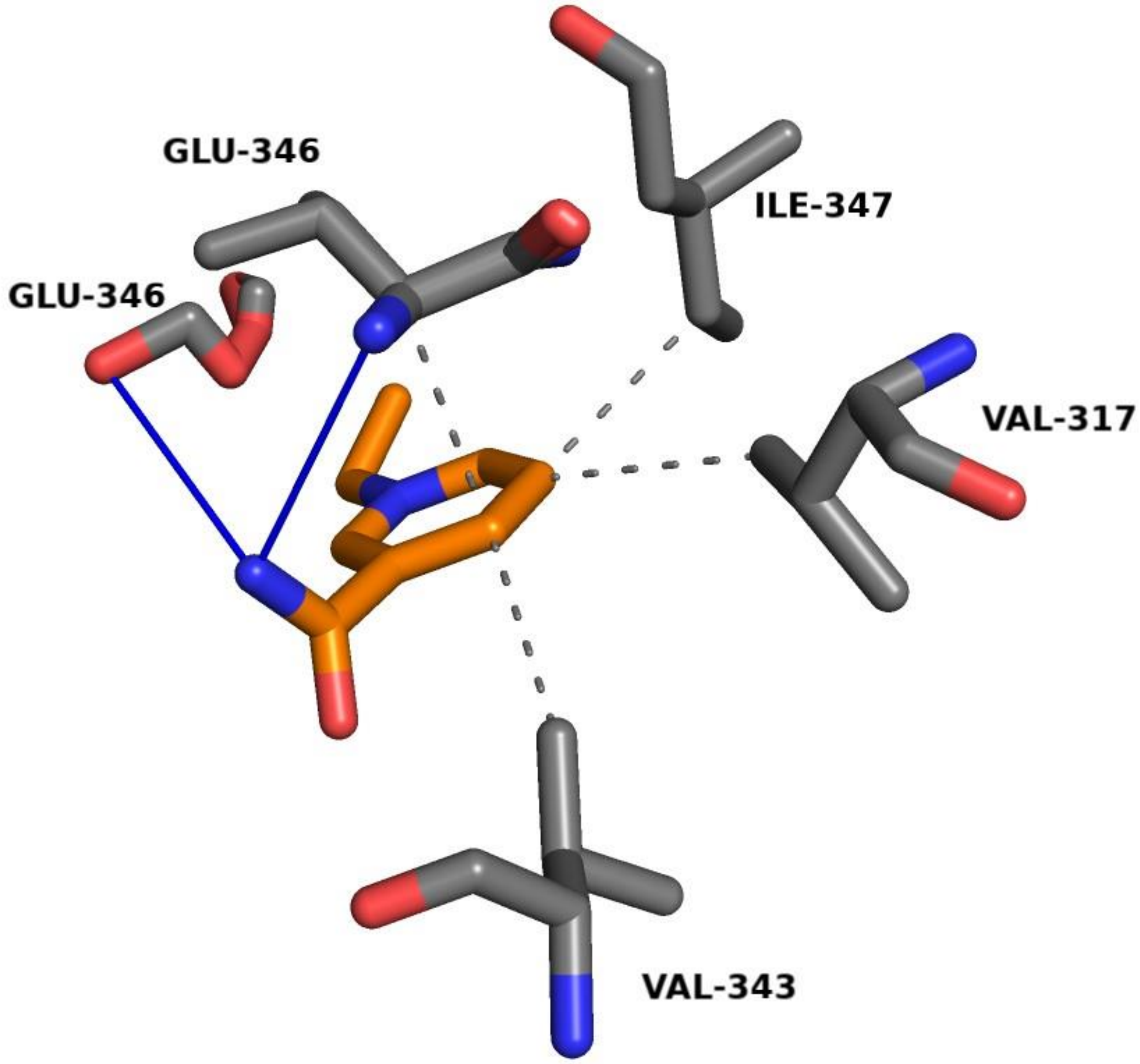

Figure S51. **Docked pose of *N*-butylnicotinamide cation (2, binding energy -5.10 kcal/mol) with the active site region of cyclooxygenase-2 (PDB ID: 1CX2) enzyme. Hydrogen bonds were indicated by blue lines, hydrophobic interactions by grey dotted lines. The ligand was highlighted in orange.**

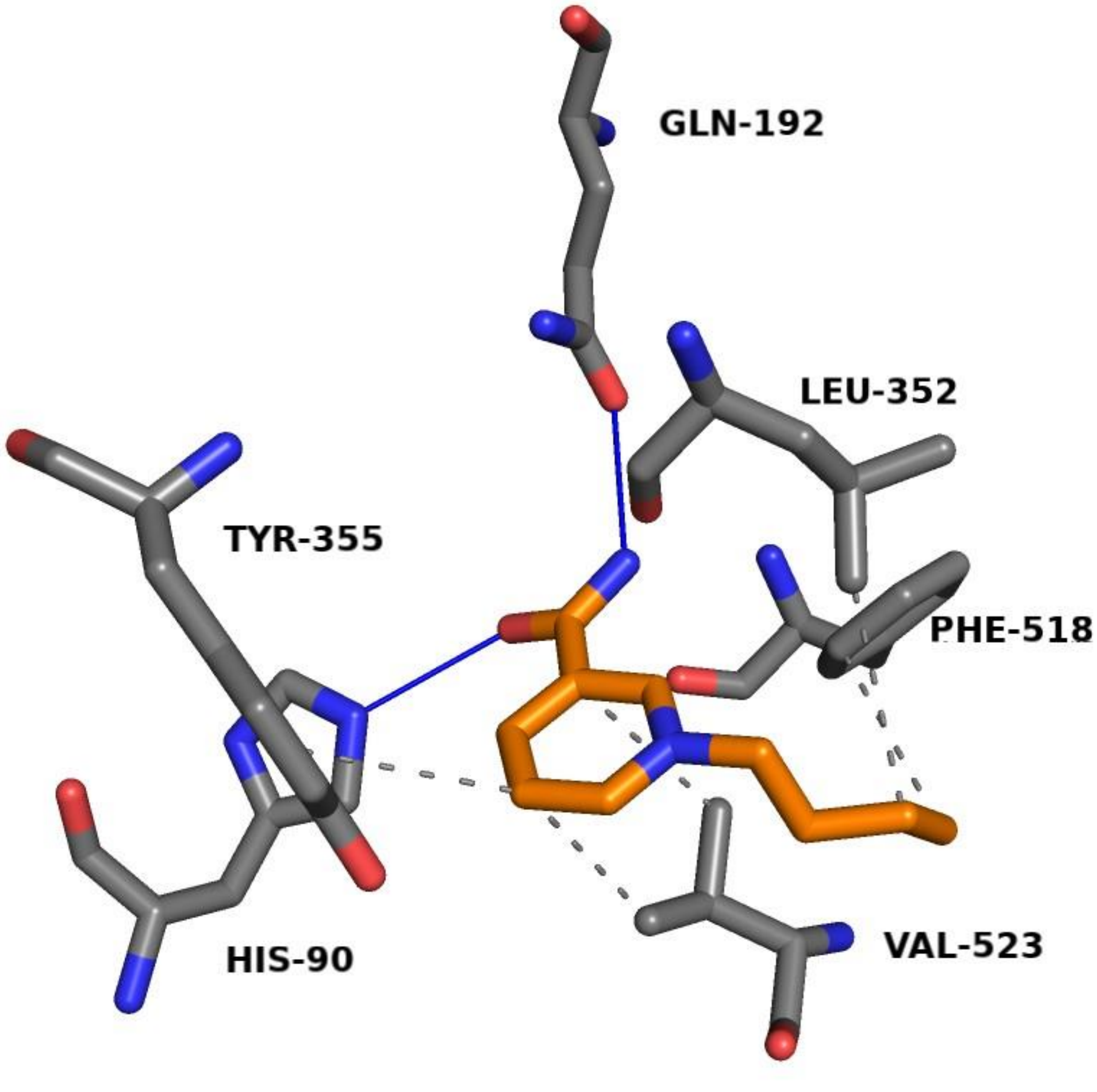

Figure S52. **Docked pose of *N*-hexylnicotinamid*e* cation (3, binding energy -6.05 kcal/mol) with the active site region of cyclooxygenase-2 (PDB ID: 1CX2) enzyme. Hydrogen bonds were indicated by blue lines, hydrophobic interactions by grey dotted lines. The ligand was highlighted in orange.**

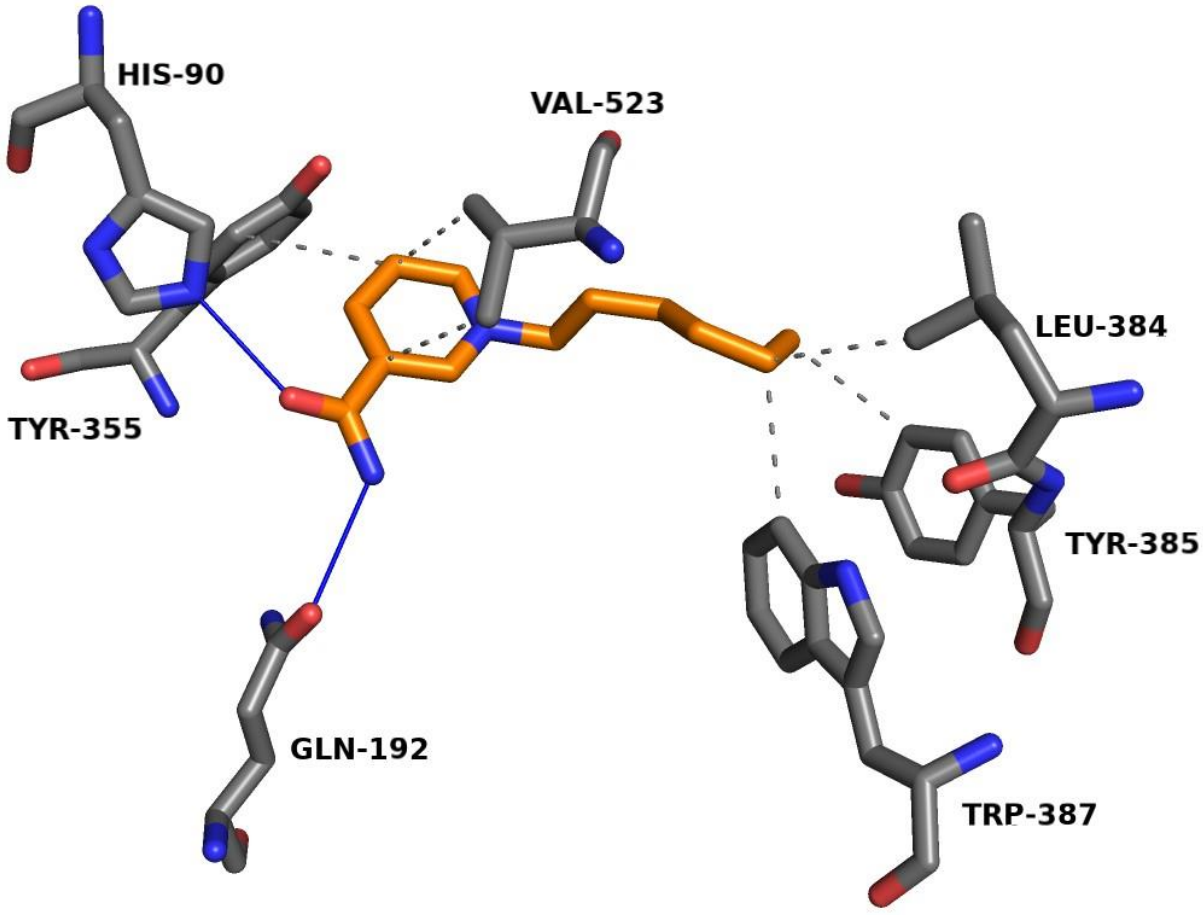

Figure S53. **Docked pose of *N*-octylnicotinamide cation (4, binding energy -6.72 kcal/mol) with the active site region of cyclooxygenase-2 (PDB ID: 1CX2) enzyme. Hydrogen bonds were indicated by blue lines, hydrophobic interactions by grey dotted lines. The ligand was highlighted in orange.**

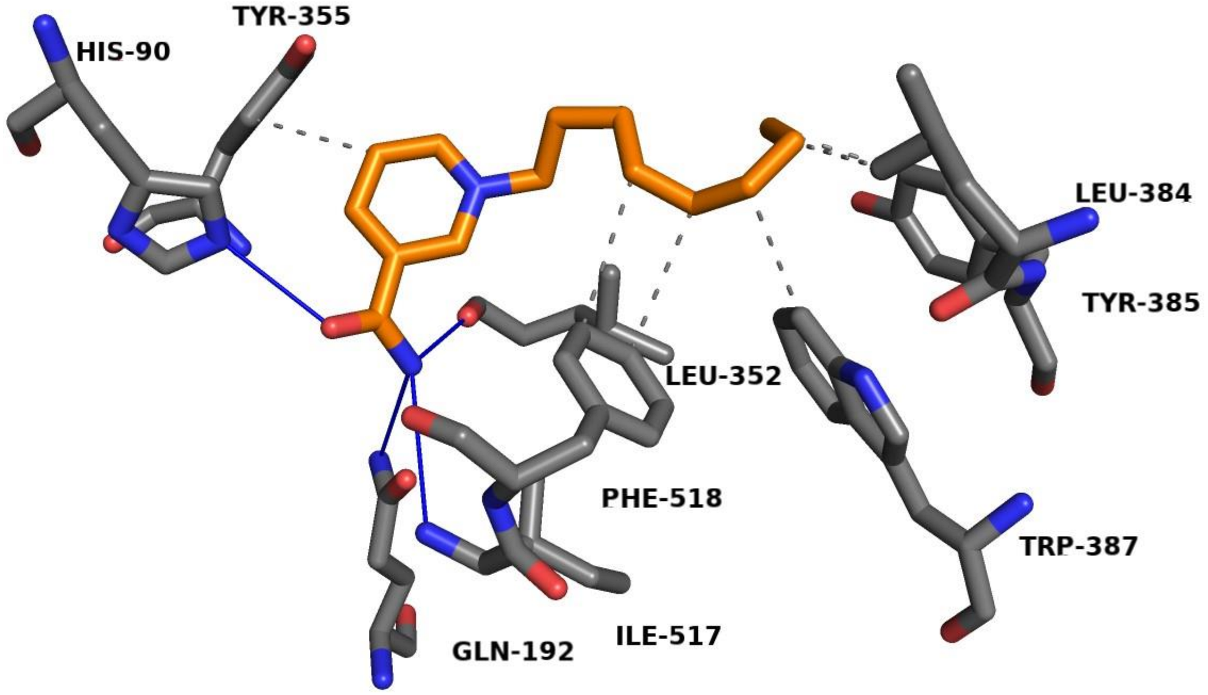

Figure S54. **Docked pose of *N*-decylnicotinamide cation (5, binding energy -6.95 kcal/mol) with the active site region of cyclooxygenase-2 (PDB ID: 1CX2) enzyme. Hydrogen bonds were indicated by blue lines, hydrophobic interactions by grey dotted lines. The ligand was highlighted in orange.**

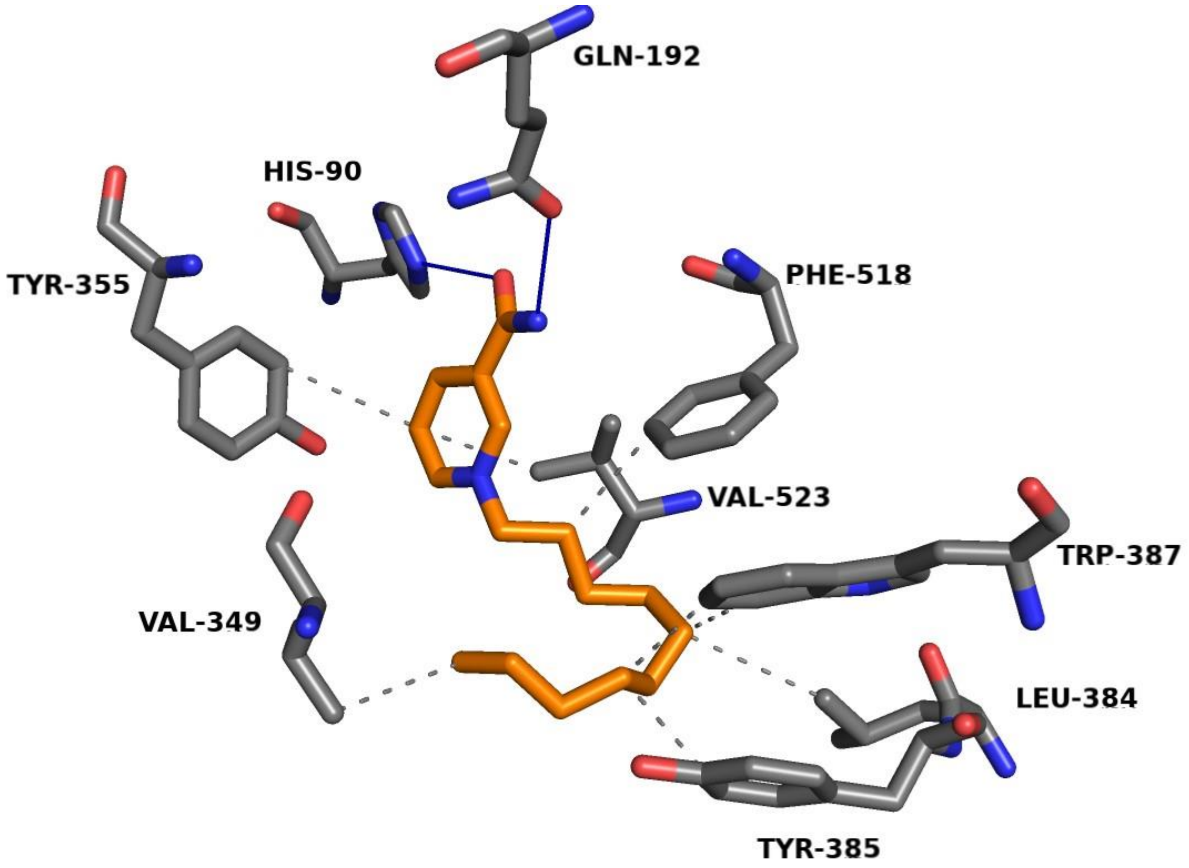

Figure S55. **Docked pose of *N*-dodecylnicotinamide cation (6, binding energy -7.46 kcal/mol) with the active site region of cyclooxygenase-2 (PDB ID: 1CX2) enzyme. Hydrogen bonds were indicated by blue lines, hydrophobic interactions by grey dotted lines. The ligand was highlighted in orange.**

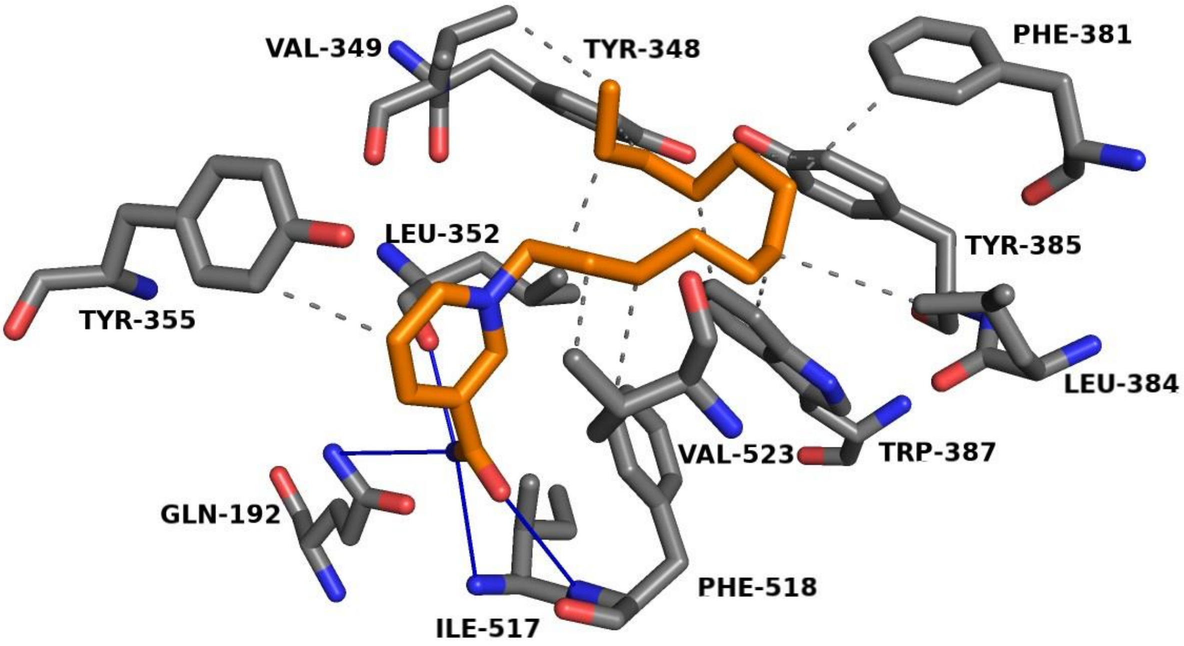

Figure S56. **Docked pose of *N*-tetradecylnicotinamide cation (7, binding energy -7.46 kcal/mol) with the active site region of cyclooxygenase-2 (PDB ID: 1CX2) enzyme. Hydrogen bonds were indicated by blue lines, hydrophobic interactions by grey dotted lines. The ligand was highlighted in orange.**

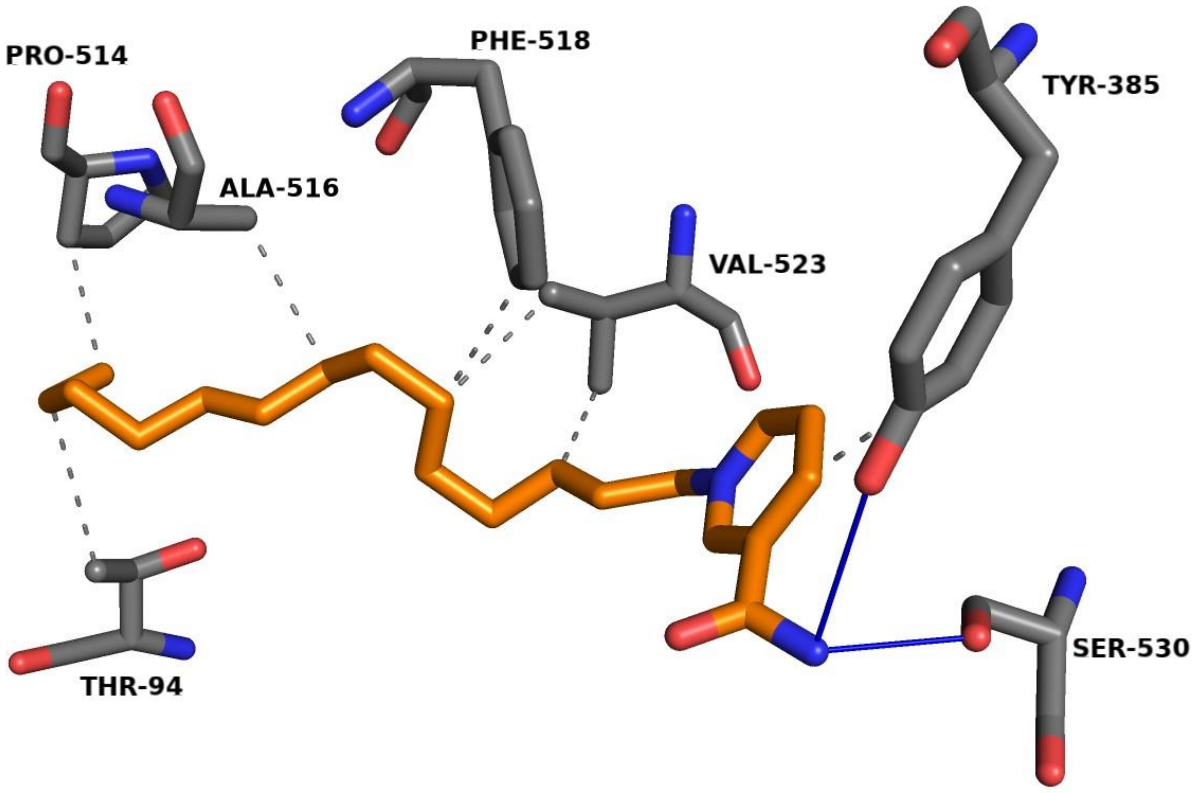

Table S9. **Interactions of S58 with residues according to PLIP.**

Hydrophobic Interactions

No	Residue	AA	Distance	Ligand Atom	Protein Atom
**1**	349A	VAL	3.69	5447	3143
**2**	352A	LEU	3.47	5460	3178
**3**	518A	PHE	3.62	5461	4857
**4**	523A	VAL	3.46	5461	4900
**5**	523A	VAL	3.60	5464	4899
**6**	527A	ALA	3.62	5452	4930

Hydrogen Bonds

No	Residue	AA	Distance H-A	Distance D-A	Donor Angle	Protein donor?	Side chain	Donor Atom	Acceptor Atom
**1**	90A	HIS	2.14	3.05	152.61	yes	yes	554 [Npl]	5467 [O2]
**2**	192A	GLN	2.09	3.05	156.05	no	yes	5468 [N3]	1580 [O2]
**3**	355A	TYR	2.82	3.31	111.95	yes	yes	3204 [O3]	5449 [Nar]
**4**	513A	ARG	2.89	3.88	171.28	yes	yes	4810 [Ng+]	5466 [O2]
**5**	517A	ILE	2.96	3.86	153.62	yes	no	4840 [Nam]	5468 [N3]

Table S10. **Interactions of indometacine with residues according to PLIP.**

Hydrophobic Interactions					
**No**	**Residue**	**AA**	**Distance**	**Ligand Atom**	**Protein Atom**
**1**	349A	VAL	3.08	5460	3143
**2**	352A	LEU	3.19	5458	3178
**3**	523A	VAL	3.05	5457	4900
**4**	527A	ALA	3.46	5465	4930
**5**	531A	LEU	3.13	5460	4966

Hydrogen Bonds									
**No**	**Residue**	**AA**	**Distance H-A**	**Distance D-A**	**Donor Angle**	**Protein donor?**	**Side chain**	**Donor Atom**	**Acceptor Atom**
**1**	522A	MET	1.85	2.79	157.39	no	no	5471 [O.co2]	4888 [O2]
**2**	526A	GLY	2.77	3.13	102.26	yes	no	4921 [Nam]	5471 [O.co2]

***Table S11*. Interactions of salicylic acid with residues according to PLIP.**

Hydrophobic Interactions					
**No**	**Residue**	**AA**	**Distance**	**Ligand Atom**	**Protein Atom**
**1**	352A	LEU	3.37	5447	3178
**2**	518A	PHE	3.23	5448	4857
**3**	523A	VAL	3.16	5448	4900
**4**	523A	VAL	3.54	5452	4899

Hydrogen Bonds									
**No**	**Residue**	**AA**	**Distance H-A**	**Distance D-A**	**Donor Angle**	**Protein donor?**	**Side chain**	**Donor Atom**	**Acceptor Atom**
**1**	90A	HIS	2.12	2.82	126.08	yes	yes	554 [Npl]	5457 [O3]
**2**	353A	SER	2.09	3.07	173.67	no	no	5455 [O.co2]	3183 [O2]
**3**	353A	SER	1.87	2.80	156.19	no	no	5457 [O3]	3183 [O2]
**4**	517A	ILE	3.13	3.99	146.83	yes	no	4840 [Nam]	5454 [O.co2]

Salt Bridges						
**No**	**Residue**	**AA**	**Distance**	**Protein positive?**	**Ligand Group**	**Ligand Atoms**
**1**	90A	HIS	5.06	yes	Carboxylate	5454, 5455

***Table S12*. Interactions of ibuprofen (IBU) with residues according to PLIP.**

Hydrophobic Interactions					
**No**	**Residue**	**AA**	**Distance**	**Ligand Atom**	**Protein Atom**
**1**	352A	LEU	3.07	5449	3178
**2**	355A	TYR	3.37	5454	3201
**3**	385A	TYR	3.78	5462	3531
**4**	518A	PHE	3.19	5448	4857
**5**	523A	VAL	3.03	5448	4900
**6**	523A	VAL	3.27	5452	4899
**7**	527A	ALA	3.85	5451	4930

Hydrogen Bonds									
**No**	**Residue**	**AA**	**Distance H-A**	**Distance D-A**	**Donor Angle**	**Protein donor?**	**Side chain**	**Donor Atom**	**Acceptor Atom**
**1**	352A	LEU	1.92	2.84	157.09	no	no	5457 [O.co2]	3174 [O2]

Salt Bridges						
**No**	**Residue**	**AA**	**Distance**	**Protein positive?**	**Ligand Group**	**Ligand Atoms**
**1**	90A	HIS	4.73	yes	Carboxylate	5456, 5457
**2**	513A	ARG	5.32	yes	Carboxylate	5456, 5457

***Table S13*. Interactions of acetylsalicylic acid (SAL) with residues according to PLIP.**

Hydrophobic Interactions					
**No**	**Residue**	**AA**	**Distance**	**Ligand Atom**	**Protein Atom**
**1**	349A	VAL	3.91	5459	3144
**2**	352A	LEU	3.51	5459	3178
**3**	381A	PHE	3.91	5451	3489
**4**	384A	LEU	3.12	5452	3519
**5**	385A	TYR	3.43	5451	3531
**6**	387A	TRP	3.54	5447	3560

Hydrogen Bonds									
**No**	**Residue**	**AA**	**Distance H-A**	**Distance D-A**	**Donor Angle**	**Protein donor?**	**Side chain**	**Donor Atom**	**Acceptor Atom**
**1**	385A	TYR	2.11	2.89	138.09	yes	yes	3533 [O3]	5460 [O2]
**2**	523A	VAL	1.86	2.81	161.55	no	no	5455 [O.co2]	4897 [O2]

Table S14. **Interactions of nicotinamide (NA) with residues according to PLIP.**

**Hydrophobic Interactions**					
**No**	**Residue**	**AA**	**Distance**	**Ligand Atom**	**Protein Atom**
**1**	352A	LEU	3.62	5447	3178
**2**	518A	PHE	3.57	5448	4857
**3**	523A	VAL	3.43	5448	4900
**4**	523A	VAL	3.77	5447	4899

**Hydrogen Bonds**									
**No**	**Residue**	**AA**	**Distance H-A**	**Distance D-A**	**Donor Angle**	**Protein donor?**	**Side chain**	**Donor Atom**	**Acceptor Atom**
**1**	90A	HIS	2.05	2.89	141.97	yes	yes	554 [Npl]	5454 [O2]
**2**	192A	GLN	3.27	3.87	121.68	yes	yes	1581 [Nam]	5455 [Nam]
**3**	352A	LEU	2.10	2.85	128.91	no	no	5455 [Nam]	3174 [O2]
**4**	517A	ILE	2.93	3.87	161.96	yes	no	4840 [Nam]	5455 [Nam]

Table S15. **Interactions of *N*-ethylnicotinamide cation with residues according to PLIP.**

Hydrophobic Interactions					
**No**	**Residue**	**Type**	**Distance**	**Ligand Atom**	**Receptor Atom**
**1**	317A	VAL	3.74	2403	866
**2**	343A	VAL	3.80	2404	1083
**3**	346A	GLU	3.57	2404	1106
**4**	347A	ILE	3.24	2403	1128

Hydrogen Bonds									
**No**	**Residue**	**Type**	**Distance H-A**	**Distance D-A**	**Donor Angle**	**Receptor donor?**	**Side chain**	**Donor Atom**	**Acceptor Atom**
**1**	346A	GLU	2.94	3.65	127.97	yes	no	1103 [N3]	2411 [Nam]
**2**	346A	GLU	2.85	3.66	136.89	no	yes	2411 [Nam]	1119 [O3]

Table S16. **Interactions of *N*-butylnicotinamide cation with residues according to PLIP.**

Hydrophobic Interactions					
**No**	**Residue**	**AA**	**Distance**	**Ligand Atom**	**Protein Atom**
**1**	352A	LEU	3.81	5460	3178
**2**	355A	TYR	3.88	5447	3201
**3**	518A	PHE	3.76	5461	4857
**4**	523A	VAL	3.73	5449	4900
**5**	523A	VAL	3.49	5447	4899

Hydrogen Bonds									
**No**	**Residue**	**AA**	**Distance H-A**	**Distance D-A**	**Donor Angle**	**Protein donor?**	**Side chain**	**Donor Atom**	**Acceptor Atom**
**1**	90A	HIS	2.17	3.02	144.18			554 [Npl]	5454 [O2]
**2**	192A	GLN	2.24	3.10	141.10			5455 [Nam]	1580 [O2]

***Table S17*. Interactions of *N*-hexylnicotinamide cation with residues according to PLIP.**

Hydrophobic Interactions					
**No**	**Residue**	**AA**	**Distance**	**Ligand Atom**	**Protein Atom**
**1**	355A	TYR	3.94	5447	3201
**2**	384A	LEU	3.61	5462	3519
**3**	385A	TYR	3.52	5463	3531
**4**	387A	TRP	3.42	5462	3560
**5**	523A	VAL	3.75	5449	4900
**6**	523A	VAL	3.61	5447	4899

Hydrogen Bonds									
**No**	**Residue**	**AA**	**Distance H-A**	**Distance D-A**	**Donor Angle**	**Protein donor?**	**Side chain**	**Donor Atom**	**Acceptor Atom**
**1**	90A	HIS	2.07	2.95	147.97	yes	Yes	554 [Npl]	5454 [O2]
**2**	192A	GLN	2.30	3.14	139.79	no	yes	5455 [Nam]	1580 [O2]

***Table S18*. Interactions of *N*-octylnicotinamide cation with residues according to PLIP.**

Hydrophobic Interactions					
**No**	**Residue**	**AA**	**Distance**	**Ligand Atom**	**Protein Atom**
**1**	355A	TYR	3.63	5447	3201
**2**	384A	LEU	3.33	5464	3519
**3**	385A	TYR	3.37	5465	3531
**4**	387A	TRP	3.45	5463	3560
**5**	518A	PHE	3.68	5461	4857
**6**	518A	PHE	3.66	5462	4859

Hydrogen Bonds									
**No**	**Residue**	**AA**	**Distance H-A**	**Distance D-A**	**Donor Angle**	**Protein donor?**	**Side chain**	**Donor Atom**	**Acceptor Atom**
**1**	90A	HIS	2.41	3.15	131.76	yes	yes	554 [Npl]	5454 [O2]
**2**	192A	GLN	3.30	3.91	122.27	yes	yes	1581 [Nam]	5455 [Nam]
**3**	352A	LEU	2.05	2.84	133.65	no	no	5455 [Nam]	3174 [O2]
**4**	517A	ILE	2.97	3.91	161.47	yes	no	4840 [Nam]	5455 [Nam]

***Table S19*. Interactions of *N*-decylnicotinamide cation with residues according to PLIP.**

Hydrophobic Interactions					
**No**	**Residue**	**AA**	**Distance**	**Ligand Atom**	**Protein Atom**
**1**	349A	VAL	3.70	5467	3144
**2**	355A	TYR	3.80	5447	3201
**3**	384A	LEU	3.84	5462	3519
**4**	385A	TYR	3.37	5464	3531
**5**	387A	TRP	3.53	5462	3560
**6**	387A	TRP	3.37	5464	3562
**7**	518A	PHE	3.43	5459	4857
**8**	523A	VAL	3.53	5447	4899

Hydrogen Bonds									
**No**	**Residue**	**AA**	**Distance H-A**	**Distance D-A**	**Donor Angle**	**Protein donor?**	**Side chain**	**Donor Atom**	**Acceptor Atom**
**1**	90A	HIS	2.40	3.24	143.28	yes	yes	554 [Npl]	5454 [O2]
**2**	192A	GLN	2.25	3.12	142.64	no	yes	5455 [Nam]	1580 [O2]

***Table S20*. Interactions of *N*-dodecylnicotinamide cation with residues according to PLIP.**

Hydrophobic Interactions					
**No**	**Residue**	**AA**	**Distance**	**Ligand Atom**	**Protein Atom**
**1**	348A	TYR	3.66	5467	3132
**2**	349A	VAL	3.45	5469	3144
**3**	352A	LEU	3.41	5468	3178
**4**	355A	TYR	3.56	5448	3201
**5**	381A	PHE	3.48	5464	3489
**6**	384A	LEU	3.69	5463	3519
**7**	385A	TYR	3.26	5465	3531
**8**	387A	TRP	3.56	5463	3560
**9**	387A	TRP	3.33	5466	3562
**10**	518A	PHE	3.89	5460	4857
**11**	523A	VAL	3.09	5459	4899

Hydrogen Bonds									
**No**	**Residue**	**AA**	**Distance H-A**	**Distance D-A**	**Donor Angle**	**Protein donor?**	**Side chain**	**Donor Atom**	**Acceptor Atom**
**1**	192A	GLN	3.29	3.85	117.98	yes	yes	1581 [Nam]	5456 [Nam]
**2**	352A	LEU	1.91	2.74	137.83	no	no	5456 [Nam]	3174 [O2]
**3**	517A	ILE	2.69	3.63	161.84	yes	no	4840 [Nam]	5456 [Nam]
**4**	518A	PHE	3.00	3.88	149.53	yes	no	4849 [Nam]	5455 [O2]

Table S21. **Interactions of *N*-tetradecylnicotinamide cation with residues according to PLIP.**

Hydrophobic Interactions					
**No**	**Residue**	**AA**	**Distance**	**Ligand Atom**	**Protein Atom**
**1**	94A	THR	3.90	5470	596
**2**	385A	TYR	3.57	5450	3531
**3**	514A	PRO	3.40	5471	4822
**4**	516A	ALA	3.39	5465	4838
**5**	518A	PHE	3.49	5463	4857
**6**	523A	VAL	3.24	5463	4900
**7**	523A	VAL	2.90	5460	4899

Hydrogen Bonds									
**No**	**Residue**	**AA**	**Distance H-A**	**Distance D-A**	**Donor Angle**	**Protein donor?**	**Side chain**	**Donor Atom**	**Acceptor Atom**
**1**	385A	TYR	2.87	3.52	126.61	yes	yes	3533 [O3]	5457 [Nam]
**2**	530A	SER	1.93	2.88	156.69	no	yes	5457 [Nam]	4956 [O3]
